# Heat capacity estimation of complex materials for energy technologies

**DOI:** 10.1016/j.joule.2025.102054

**Published:** 2025-08-20

**Authors:** Elana J. Cope, Joana Bustamante, Zöe M. Johnson, Alicia Lancaster, Ramya Gurunathan, Janine George, Matthias T. Agne

**Affiliations:** 1 Department of Chemistry and Biochemistry, University of Oregon, Eugene, OR, USA; 2 Federal Institute for Materials Research and Testing (BAM), 12205 Berlin, Germany; 3 Independent Contributor, Durham, NC, USA; 4 Institute of Condensed Matter Theory and Solid-State Optics, Friedrich Schiller University, 07743 Jena, Germany; 5 Materials Science Institute, University of Oregon, Eugene, OR, USA

**Keywords:** heat capacity, thermal conductivity, phase transition, energy materials, batteries, thermoelectrics, thermal properties, phonons

## Abstract

Heat capacity, which directly relates to free energy changes and thermal transport, is fundamental to modern engineering design. Even though current computational technology provides a detailed picture of atomic vibrations, the Debye and Dulong-Petit models are still widely utilized despite being prone to lower accuracy. Modern considerations of vibrational states, anharmonicity, electronic carriers, and phase transformations could improve estimates. Herein, the physics-based vibrational + dilation + electronic (VDE) model incorporates a user-provided phonon density of states, a phonon pressure-based dilation term, and an electronic component. Phonon density of states from analytical, machine-learned, and first-principles methods are compared, thus highlighting the advantages of machine-learned technology. Heat capacity estimates for 38 diverse materials are often within 5% of experimental values between 200 and 600 K. Detailed temperature-dependent investigations are carried out for several materials, including $\text{LiCo}O_{2}$, ZIF-8, ${\text{Mg}}_{\text{3}}{\text{Sb}}_{\text{2}}$, polyvinyl chloride (PVC), and amorphous silicon. $Cu_{2}$Se is modeled through its phase transition, which further demonstrates the model’s capabilities to enable engineering design and sophisticated analysis.

## Context & scale

The control of heat in energy materials is one of the greatest current engineering challenges. Accurate estimations of heat capacity are key in creating and using materials safely and efficiently. Current models for heat capacity are often limited due to crude estimations of the phonon density of states, which is a key component of the thermodynamic definition of heat capacity. Utilization of a more detailed phonon density of states, which can easily be obtained from machine-learned algorithms, combined with dilation and electronic contributions, yields heat capacity estimations that are 29% better than the widely utilized Debye model and are comparable to state-of-the-art quantum mechanical calculations. The framework and necessary tools for heat capacity estimations demonstrated herein can be built into more detailed models and analyses, such as high-throughput characterization, transport models, or other thermodynamic assessments. Consequently, the so-called vibrational + dilation + electronic (VDE) model of heat capacity developed in this work better enables the informed design of next-generation devices.

## Graphical abstract

**Figure:**
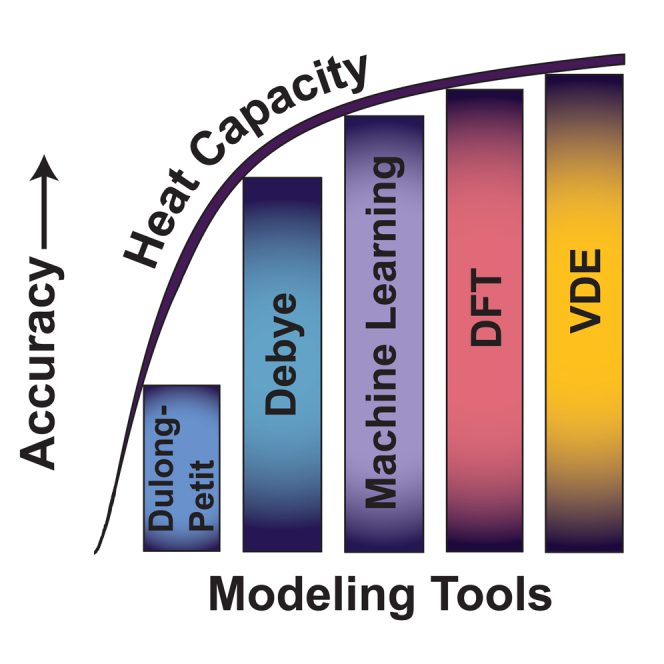

## Teaser

Estimates of heat capacity are vital to a wide range of engineering applications. From a comprehensive understanding of heat capacity, a physics-based model is presented that incorporates analytical, machine-learned, and first-principles methods to accurately describe experimental values. Using different assessments of the vibrational density of states, a novel dilation term, and an electronic component, the so-called VDE model provides accessible estimates of heat capacity within ∼5% across a wide temperature range.

## Introduction

Thermal management is the crux of many modern engineering design problems. Effective control over the spatial temperature profile in electronic and chemical systems is often a limiting factor for device operation and performance.^1^^,^^2^ Consequences of failure in thermal management range from inefficient energy usage at best to dangerous catastrophes at worst. For example, inclusion of materials with mismatched thermal expansion coefficients could cause the thermal barrier coatings on turbine blades to crack^3^ or semiconductor interfaces of a thermoelectric module to fail.^4^ Catastrophic failure of lithium-ion batteries occurs when temperatures rise high enough to promote self-propagating reactions,^5^^,^^6^^,^^7^^,^^8^ a process named as thermal runaway. Microprocessors,^9^ latent heat storage systems,^10^ and quantum technologies^11^ benefit from fast heat transport, whereas refractory processes^12^ and industrial chemical reactions often benefit from heat retention. Considerations of the temperature dependence of chemical stability, e.g., for organic and perovskite materials used in optoelectronic and photovoltaic devices,^13^^,^^14^ and the optimization of operating temperatures, e.g., for metal-organic frameworks (MOFs) used in carbon capture,^15^ are key components of technological success. Thermoelectric devices, which utilize the solid-state Seebeck effect to convert heat directly into electricity, represent a unique challenge in that a large temperature gradient must be sustained across the device, necessitating the development of ultra-low thermal conductivity materials.^16^ Thus, an accurate description of the thermal properties of materials is integral to next-generation device design. In particular, heat capacity and thermal conductivity must be well understood in order to accurately model the thermodynamic state and temperature distributions during device operation.

Heat capacity describes how much heat (thermal energy) must be added or removed from the material to change the temperature by a certain amount. It is a fundamental thermodynamic quantity whose magnitude is defined by the various microscopic mechanisms available for heat storage. In solids, this means that heat capacity is intimately related to the frequency distribution of atomic vibrations that store thermal energy in phonon occupations.^17^ Additionally, electronic carriers or phenomena such as phase transformations^18^ can also contribute to the total heat capacity. It follows that the estimation and analysis of heat capacity in technological materials requires an understanding of the microscopic mechanisms at play in the temperature range of interest.

The thermodynamic relationship between thermal conductivity κ, thermal diffusivity D, density ρ, and heat capacity (at constant pressure) $c_{p}$,^19^

(Equation 1) $$\kappa =\rho c_{p}D\text{,}$$

means that estimations of κ from thermal diffusivity measurements are heavily dependent on accurate values of $c_{p}$. In this case, $c_{p}$ is an intrinsic quantity with units of J${\text{·g}}^{\text{−1}}{\text{·K}}^{\text{−1}}$ and is multiplied by ρ to obtain the heat capacity per volume with units of J${\text{·m}}^{\text{−3}}{\text{·K}}^{\text{−1}}$ in the relation $C_{p}=\rho c_{p}$. For thermoelectric materials, inaccurate estimates of heat capacity in Equation 1 will directly impact the estimation of the thermoelectric conversion efficiency.^19^ Given the widespread use of Equation 1 for estimating thermal conductivity near room temperature and above, this work primarily focuses on accurately characterizing heat capacity at these temperatures (>100 K), but low-temperature (>2 K) investigations are also demonstrated.

The relevance of heat capacity to other thermal properties such as thermal expansion^20^ $\left(\alpha \propto C_{p}\right)$ and thermal effusivity^21^ $\left(\epsilon \propto \sqrt{C_{p}}\right)$ makes it foundational to understanding thermophysical properties broadly, in addition to being essential for thermodynamic assessments.^22^^,^^23^ As scientists and engineers become increasingly interested in developing diverse materials such as those for energy conversion/storage, semiconductor and quantum technologies, etc., the ability to accurately estimate the magnitude and temperature dependence of heat capacity is crucial for material analysis and development. Because experimental heat capacity measurements can be inconsistent, particularly at high temperatures where independent measurements can deviate by ∼20%,^24^ reliable physics-based estimates of heat capacity offer a straightforward solution. Historically, the Dulong-Petit and Debye models of heat capacity have been widely used because they are easily determined from accessible material properties, but they have limitations. For example, the Debye model aims to estimate the maximum vibrational frequency of a solid but is actually a better estimate of the average vibrational frequency for many materials.^25^^,^^26^ Various attempts to improve beyond the Debye model have been undertaken in thermal conductivity models.^26^^,^^27^ Although many analytical models of the vibrational spectrum do capture the first-order physics of heat capacity, it is now possible to build a more detailed model with similarly accessible inputs. Herein, a method of estimating heat capacity is presented that utilizes a crystallographic input file (CIF) structure file and material properties such as the bulk and shear moduli (which often can be easily obtained from sources like Materials Project). Importantly, this study provides a starting point for constructing more detailed models of heat capacity, including through phase transformation regions.

## Thermodynamic definition of heat capacity

As most practical experimental characterization of solids is undertaken under constant pressure conditions, the focus of this work will be to describe the heat capacity at constant pressure, written as an intrinsic quantity (normalized by volume) as

(Equation 2) $$C_{p}=\frac{1}{V}{\left(\frac{\partial H}{\partial T}\right)}_{p}\text{,}$$

where H is the total enthalpy contained by the material with volume V. Although enthalpy is often dominated by thermal processes, it can also have contributions from phase transformations. In the absence of phase transformations, the constant pressure heat capacity $C_{p}$ (in units of J${\text{·m}}^{\text{−3}}{\text{·K}}^{\text{−1}}$) can be defined by the thermodynamic relation

(Equation 3) $$C_{p}=C_{V}+B\alpha^{2}T=C_{V}\left(1+\gamma \alpha T\right)$$

that relates to heat capacity at constant volume $C_{V}$, the isothermal bulk modulus B, and volumetric thermal expansion coefficient α or equivalently with the thermodynamic Grüneisen parameter γ. Implicitly, all the terms in Equation 3 are temperature dependent. Inspection of Equation 3 already suggests that $C_{p}$ may be described to varying degrees of approximation, with $C_{V}$ being a good first approximation that can be improved upon by considering the so-called dilation component that has an explicit linear temperature dependence $\left(B\alpha^{2}T\right)$.

In anticipation of the importance of $C_{V}$ in estimating the magnitude of $C_{p}$, it is important to note that $C_{V}$ is defined from the total internal energy U (at constant volume) as

(Equation 4) $$C_{V}=\frac{1}{V}{\left(\frac{\partial U}{\partial T}\right)}_{V}\text{,}$$

which is particularly beneficial as U is often accessible to estimate from theoretical considerations. The isothermal bulk modulus, $B=-{\left(\partial p/\partial \ln\;V\right)}_{T}$, is an elastic modulus of the material, whereas the thermal expansion coefficient, $\alpha ={\left(\partial \ln\;V/\partial T\right)}_{p}$, and thermodynamic Grüneisen parameter, $\gamma =\alpha B/C_{V}$, are anharmonic properties of the solid that describe the thermal pressure that drives the material’s change in volume with temperature. From the point of view that heat capacity is related to the degrees of freedom available for the material to store thermal energy, the dilation term represents the additional heat that can be stored by doing work against the surroundings to make more space for itself.

## Microscopic considerations of heat capacity

Dulong and Petit made the empirical observation (ca. 1819) that solids have a heat capacity of $∼3k_{B}$ per atom near room temperature, where $k_{B}$ is the Boltzmann constant and is related to the gas constant R through Avogadro’s number $N_{A}$ in the relation $R=N_{A}k_{B}$. From the equipartition theorem, which postulates that energy is equally divided between available degrees of freedom, this suggested that atomic motion is likely a dominant contributor to heat capacity. In the kinetic theory of ideal monatomic gases, kinetic energy distributed across three spatial dimensions gives heat capacity a value of $3k_{B}/2$ per atom (at constant volume). Atoms vibrating in solids have kinetic energy, but they also have strong interatomic interactions (bonds) that contribute an additional $3k_{B}/2$ per atom from potential energy, bringing the total to $3k_{B}$ per atom for a classical harmonic oscillator. Note that deviations from $3k_{B}$ per atom are possible when considering anharmonic oscillators at constant volume, which may amount to an increase in the contribution from $C_{V}$ (Equation 4) by a few percent near room temperature and ∼10% at 1,500 K.^28^ Nevertheless, the Dulong-Petit model of heat capacity is applicable when atomic vibrations can be considered as classical harmonic oscillators at high temperatures.

Because the Dulong-Petit heat capacity is representative of the heat capacity in the high-temperature limit, the thermoelectrics community has a long history of using it to approximate the heat capacity at temperatures near and above 300 K.^29^ The maximum vibrational frequencies of the atoms in many thermoelectric materials are already excited by these temperatures, such that around room temperature, the relation $C_{p}\approx C_{V}\approx 3k_{B}/$atom oftentimes holds. However, in many engineering solids relevant for semiconductor/microelectronic, photovoltaic, battery, or mechanical applications, the highest vibrational frequencies are not significantly excited by 300 K, thus making that model insufficient. For example, at 300 K, the heat capacity of silicon is only ∼80% of the Dulong-Petit value.^30^ Additionally, heat capacity can have a strong temperature dependence around room temperature. Meyer-Kelly-type equations for temperature-dependent heat capacity can often be found in reference books, but these empirical descriptions are fit to experimental data and cannot be determined a priori. Using a physics-based model for heat capacity that captures temperature dependence is distinctly advantageous for computational materials design or in the absence of experimental capabilities.

At lower temperatures, heat capacity drops sharply from the Dulong-Petit value. Einstein recognized that this results from the fact that solids are quantum objects with changing occupations of the quantized states of different vibrational modes.^31^ There is not enough thermal energy available at low temperature to populate higher vibrational states, so those vibrations are “frozen out.” Using quantum statistics, he was able to better predict the temperature dependence of heat capacity while still reaching the Dulong-Petit value at high temperature. Later, Debye proposed that atomic vibrations have a distribution of frequencies, unlike the single-frequency approximation that Einstein made. The concept of a continuum nature of the phonon density of states led to a better prediction of the temperature dependence of heat capacity at low temperatures, famously known as the Debye T-cubed law.^31^

While the Einstein and Debye models brought temperature-dependent estimates of heat capacity closer to observed values, the crude estimates of the phonon density of states neglect the nuanced nature of atomic vibrations specific to each material, causing inaccuracies in the estimation of heat capacity. Additionally, it is commonly observed that $C_{p}$ tends to exceed the Dulong-Petit/Debye predictions at high temperatures, suggesting that the Dulong-Petit and Debye models may be a good first approximation for $C_{p}$, but a more complete description of a material’s phonon density of states and dilation propensity is expected to be more accurate.

Temperature-dependent models of $C_{p}$ can be constructed starting from Equation 3. In addition to the explicit temperature dependence of the dilation term, it is also necessary to consider each of the microscopic mechanisms that may contribute appreciably to $C_{V}$ and their temperature dependence. The total heat capacity at constant volume is then the sum of constant volume heat capacities from different sources, e.g., vibrational, electronic, etc., as

(Equation 5) $$C_{V}=C_{V}^{\text{vib}}+C_{V}^{\text{elec}}+\ldots \text{.}$$

Consideration of the vibrational component is necessary for all solids, whereas the electronic contribution may only be needed in metallic-like materials (e.g., having electronic carrier concentrations greater than $∼10^{22}$ carriers$\cdot cm^{-3}$). Other contributions to heat capacity, e.g., magnetism,^32^ can be considered as necessary. From a quantum mechanical treatment of atomic vibrations as harmonic oscillators with angular frequency ω following Bose-Einstein statistics, the vibrational contribution to heat capacity at constant volume can be described as^17^

(Equation 6) $$C_{V}^{\text{vib}}=3nk_{B}\int_{0}^{\infty }\left(\frac{g\left(\omega \right)}{3n}\right){\left(\frac{\hbar \omega }{k_{B}T}\right)}^{2}\left(e^{\frac{\hbar \omega }{k_{B}T}}\right){\left(e^{\frac{\hbar \omega }{k_{B}T}}-1\right)}^{-2}d\omega$$

for a collection of oscillators with a spectral distribution defined by the phonon density of states g(ω). Note that g(ω)/3n is called the normalized density of states $\left(\int_{0}^{\infty }\left(g\left(\omega \right)/3n\right)d\omega =1\right)$, where n is the number density of atoms (atoms·${\text{m}}^{\text{−3}}$). The electronic term for constant volume heat capacity is derived from the Sommerfeld expansion as

(Equation 7) $$C_{V}^{\text{elec}}=\frac{\pi^{2}}{3}k_{B}^{2}D\left(E_{F}\right)T\text{,}$$

where $D\left(E_{F}\right)$ is the value of the electronic density of states at the Fermi level (states ${\text{·J}}^{\text{−1}}{\text{·m}}^{\text{−3}}$). Notably, the contribution of electronic carriers to heat capacity in this model has a linear temperature dependence.

Thus, for a vast majority of materials, the temperature dependent $C_{V}$ (Equation 5) can be estimated when estimations of the phonon density of states and electronic density of states are available. Then, values of $C_{p}$ can be obtained from Equation 3 with the inclusion of experimental or computational values of bulk modulus and thermal expansion coefficient.

In this work, heat capacity values are often reported in units of J${\text{·mol}}^{\text{−1}}{\text{·K}}^{\text{−1}}$. These units are obtained by multiplying volumetric heat capacity values (i.e., Equation 3), having units of J${\text{·m}}^{\text{−3}}{\text{·K}}^{\text{−1}}$, by the molar volume of the material $V_{m}$ (${\text{m}}^{\text{−3}}{\text{·mol}}^{\text{−1}}$), where $V_{m}$ is derived from the material’s formula weight $M_{w}$ (kg$\cdot \text{mo}l^{-1}$) divided by the material’s density ρ (kg${\text{·m}}^{\text{−3}}$) as $V_{m}=M_{w}/\rho$.^24^

## Methods

**Figure 1:**
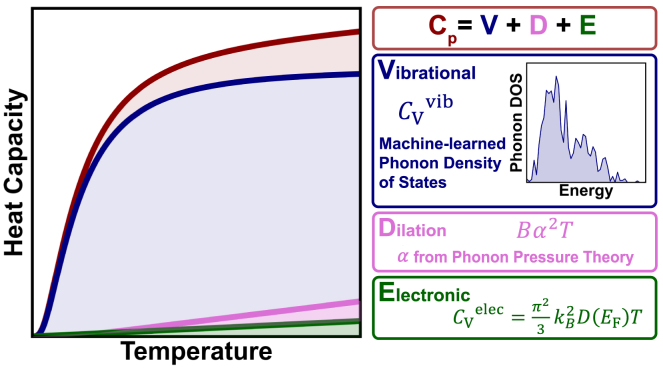
Construction of the VDE model The components of the VDE model are shown as vibrational (blue) using a machine-learned density of states algorithm, dilation (pink) using phonon pressure theory, and electronic (green) using the electronic density of states at the Fermi level, which all add together to get the VDE $C_{p}$ estimate (red).

Here, we introduce a physics-based estimation of $C_{p}$ based on Equation 3 that we call the VDE model (short for vibrational + dilation + electronic). For a detailed description of the model and relevant Python code, see Note S1. Although the VDE model requires mostly the same inputs as the Debye model, it is distinct in its incorporation of a machine-learned phonon density of states, a novel estimate of the dilation term, and the electronic term. Explicitly, the VDE model (Figure 1) accounts for the vibrational (Equation 6) and electronic (Equation 7) contributions to $C_{V}$, along with the dilation term, as

(Equation 8) $$C_{p}=C_{V}^{\text{vib}}+C_{V}^{\text{elec}}+B\alpha^{2}T\text{,}$$

which requires an estimation of the phonon density of states g(ω), the electronic density of states at the Fermi level $D\left(E_{F}\right)$, the bulk modulus B, and the thermal expansion coefficient α. The temperature dependence of g(ω), $D\left(E_{F}\right)$, B, and α is not included in the model, as the explicit temperature dependence of $C_{p}$ from Bose-Einstein statistics and the linear T terms were found to be sufficient, as will be shown. While many previous heat capacity models have relied on the Debye and Einstein models of the phonon density of states, the field of machine learning is under rapid development, and it is now possible to use a machine-learned estimate of the phonon density of states.^33^^,^^34^

In this study, the pre-trained model available in the ALIGNN Python package (model = jv_pdos_alignn) is used.^35^ ALIGNN is a graph neural network that encodes crystal structures as non-Euclidean graphs, where nodes, edges, and edge pairs capture atomic properties, bond lengths, and bond angles, respectively. The output of the ALIGNN Phonons model is a discretized phonon density of states with phonon density of states values specified in the frequency range of −300 to 1,000 ${\text{cm}}^{\text{−1}}$ (∼ −37 to 120 meV) with a bin size of 20 ${\text{cm}}^{\text{−1}}$ (∼2.5 meV). The ALIGNN phonon density of states model was trained on 14,000 phonon structures determined from density functional theory (DFT) calculations maintained by the National Institute of Standards and Technology in the NIST-JARVIS database.^36^ These phonon calculations were performed using the OptB88vdw functional using an automated k-point and energy cutoff convergence methodology.^37^ The dataset was randomly divided into an 80%-10%-10% training-validation (during training)-test (fully blind) split. Additional training and validation details can be found in the original paper presenting the model.^35^

The ALIGNN vibrational density of states estimation does not explicitly account for temperature dependence or anharmonic contributions at constant volume, but temperature-dependent structure files could be used as inputs. Other phonon density of states estimations could also be utilized, e.g., different machine-learned models,^38^^,^^39^^,^^40^ or those from DFT, force fields,^33^^,^^41^ or experiments. As a comparison, we show VDE results using phonon density of states estimates from a different machine-learned model that uses force fields, known as the MACE-MP-0-3b model^33^ with D3 dispersion^42^ (Note S1), hereafter referred to as MACE. Compared with analytic models, the ALIGNN and MACE phonon density of states offer a more accurate distribution of phonon modes, including van Hove singularities and acoustic-optical band gaps. For the purposes of estimating $C_{V}$, only the phonon density of states at positive frequencies (≥0 meV) is utilized, and negative values are set to zero before the density of states is normalized (see Note S1).

A powerful feature of the ALIGNN predictive model is that it only requires a CIF or other structure file (POSCAR or PDB) to generate the phonon density of states estimation. CIF files contain information about the atomic structure of materials, which can be determined experimentally (e.g., X-ray diffraction) or found from DFT structure optimization. In this study, we utilize the CIF files available on the Materials Project database^43^ unless otherwise stated. The ALIGNN phonon density of states output, plus Materials Project values for bulk modulus (i.e., $B_{\text{VRH}}$), shear modulus (i.e., $G_{\text{VRH}}$), electronic density of states, volume, density, and number of atoms in the structure are used in our high-throughput assessment of heat capacity. The thermal expansion coefficient is estimated from phonon pressure theory^44^ as

(Equation 9) $$\alpha \;\approx \;\frac{3}{2}\frac{C_{V}}{\rho v^{2}}\text{,}$$

where $\rho v^{2}=\left(10G+3B\right)/9$ is an average elastic modulus defined by the root mean square speed of sound. Thermal expansion is usually attributed to anharmonicity, which is related to various factors in the local bonding environment like coordination number and long-range interactions.^45^ However, thermal expansion may also be described within the context of elastic continuous medium theory, which characterizes each atom as an isolated oscillator with its own local mechanical equilibrium.^44^ In this latter case, it is possible to predict the thermal expansion coefficient from harmonic descriptors (Equation 9), and this estimation of α is expected to be within a factor of ∼2 of DFT-determined values. Given the aforementioned inputs, the VDE model yields a heat capacity prediction across a desired temperature range.

## High-throughput VDE model of heat capacity

**Figure 2:**
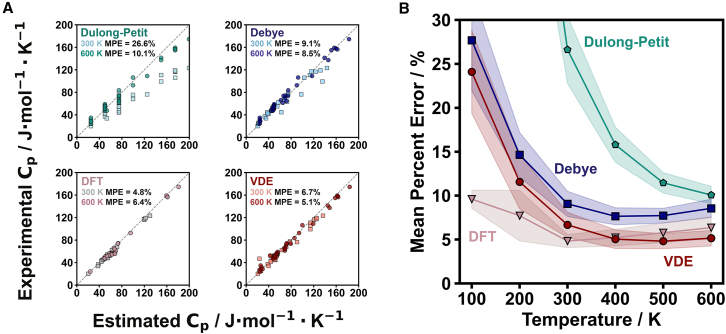
Comparison of heat capacity models with experimental values for diverse materials (A) The Dulong-Petit estimate is found to significantly overestimate heat capacity values with a mean percent error (MPE) of 26.6% at 300 K (light teal squares) and 10.1% at 600 K (dark teal circles). The Debye model estimate is found to be significantly better than the Dulong-Petit values with MPE values of 9.1% at 300 K (light blue squares) and 8.5% at 600 K (dark blue circles). DFT values at 300 K (light gray squares) have an MPE value of 4.8%, and at 600 K (pink circles), the MPE is 6.4%. The VDE model estimates are found to be comparable to DFT-level accuracy at higher temperatures with MPE values of 6.7% at 300 K (light red squares) and 5.1% at 600 K (dark red circles). (B) The MPE was assessed as a function of temperature for the Dulong-Petit (teal pentagons), Debye (blue squares), DFT (pink triangles), and VDE (red circles) models (Notes S2 and S3). The VDE model is seen to be ∼29% more accurate than the Debye model on average and comparable to DFT estimates of heat capacity over a wide temperature range. Shaded regions represent the standard error of the mean at each temperature.

Upon running the VDE model on 38 different materials, we compare their estimated $C_{p}$ with experimental $C_{p}$ values reported in the NIST-JANAF Tables^46^ (Note S2). Heat capacity estimations using the Dulong-Petit and Debye models were undertaken for comparison, as well as DFT estimations of $C_{p}$ for 25 of those materials (Note S3). Exemplary correlations of the estimated and experimental $C_{p}$ values are shown for 300 and 600 K (Figure 2A). While the Dulong-Petit and Debye models tend to overestimate experimental $C_{p}$ values, the VDE model provides a better estimate, as indicated by a lower mean percent error (MPE). In fact, by tracking the MPE as a function of temperature (Figure 2B), we find that the VDE model performs with DFT-level accuracy in estimating experimental heat capacity values across a wide temperature range (within ∼7% from 300 to 600 K) and is at least 29% more accurate than the Debye model on average. Even at the highest temperature evaluated in this comparison (600 K), the Dulong-Petit estimate still has the largest deviation from experimental values. Being temperature independent, the Dulong-Petit model performs progressively worse at lower temperatures (Note S2). Although the Debye model captures the general temperature dependence of heat capacity, it is not as predictive as the VDE model largely because it cannot capture the same degree of complexity of the phonon density of states. At higher temperatures, the dilation term becomes more important and contributes an additional benefit to the VDE model over the Debye model, as seen in the comparison between $C_{p}$ and $C_{V}$ (Note S2).

By inspection of Figure 2, it is apparent that the VDE model estimations of heat capacity are predictive of experimental values for diverse materials over a wide temperature range. Thus, we anticipate that the VDE model can be reliably used as an estimate for the heat capacity when experimental measurements are not available or as a starting point for more detailed investigation of thermophysical properties. Given the complexity of many materials, as well as limited access to low-temperature heat capacity measurements and the uncertainties (e.g., ∼20%) associated with high-temperature heat capacity measurements, the VDE model meets the essential needs for accurate predictions. While capturing typical contributions to heat capacity (i.e., vibrational, dilation, and electronic) over a large temperature range, it also provides a physically based framework for assessing additional heat capacity contributions. Moreover, the VDE model provides comparable (if not better) accuracy than DFT results at temperatures above 300 K, yet it can be run in minutes and does not require supercomputer usage. Because the VDE model requires much of the same input information as the Debye model, there is a low barrier to using the VDE model for improved heat capacity estimates (Note S1).

**Figure 3:**
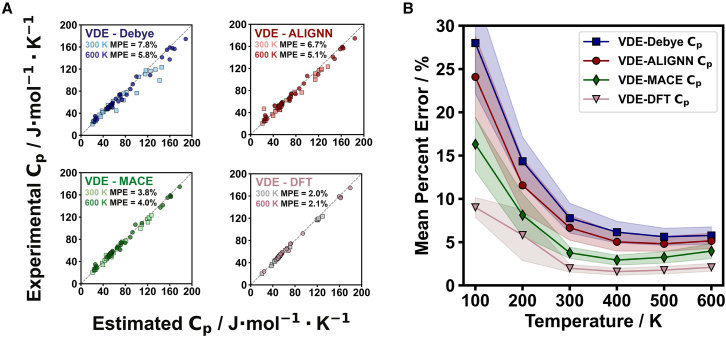
Comparison of VDE heat capacity estimates with different phonon density of states inputs (A) The VDE estimate using the Debye phonon density of states is found to have the highest mean percent error (MPE) values, with MPE of 7.8% at 300 K (light blue squares) and 5.8% at 600 K (dark blue circles). The VDE estimate using the ALIGNN phonon density of states is found to improve upon the former, with MPE values of 6.7% at 300 K (light red squares) and 5.1% at 600 K (dark red circles). When the MACE model is used as the phonon density of states input, we find the MPE to be 3.8% at 300 K (light green squares) and 4.0% at 600 K (dark green circles). VDE estimates using DFT phonon density of states inputs have very low MPE values, with the outputs (light gray squares) having MPE values of 2.0% at 300 K and 2.1% at 600 K (pink circles). (B) The MPE was assessed as a function of temperature for VDE outputs with phonon density of states estimations from the Debye model (blue squares), ALIGNN model (red circles), MACE model (green diamonds), and DFT (pink triangles). The VDE model’s dilation and electronic terms contribute to lower MPE values for all models (Notes S2 and S3). Shaded regions represent the standard error of the mean at each temperature.

Although the ALIGNN density of states estimation is featured in the VDE model (see Figure 2), it is important to note the earlier point that any phonon density of states estimation could be used in its place. To exemplify this and examine the effects of phonon density of states estimates in the VDE model, we compare the MPEs of VDE outputs that use the analytical Debye model, MACE model, and DFT-derived density of states in Equation 8 (Figure 3).

To clarify, the Debye and Dulong-Petit values featured in Figure 2 follow textbook definitions^31^ that do not consider dilation or electronic terms. The DFT values in Figure 2 include DFT-calculated dilation and electronic terms. In Figure 3, all results utilize the dilation and electronic terms defined for the VDE model.

An important conclusion from this analysis of the VDE model capabilities (Figure 3) is that both the ALIGNN and MACE estimates of the phonon density of states result in heat capacity values (MPE ∼5%) that rival DFT determinations (as seen in Figure 2). A large contributing factor is the analytical estimate of the thermal expansion coefficient used in the dilation term (Equation 9 and Note S2), which also drastically improves heat capacity estimates when using DFT-determined phonon density of states (MPE ∼2% in Figure 3; see also Note S3). Considering the computing resources needed for DFT, machine-learned estimates for density of states provide an excellent alternative. Although the Debye density of states performs comparably in this analysis, we will show that this does not hold for specific examples of complex materials.

## Heat capacity of complex materials

To better understand the scope of applicability and limitations of the VDE model, specific analysis was undertaken for a range of energy materials. Here, we use the ALIGNN estimate of the phonon density of states with comparison to the MACE model in places. For a complete comparison, see Note S7.

**Figure 4:**
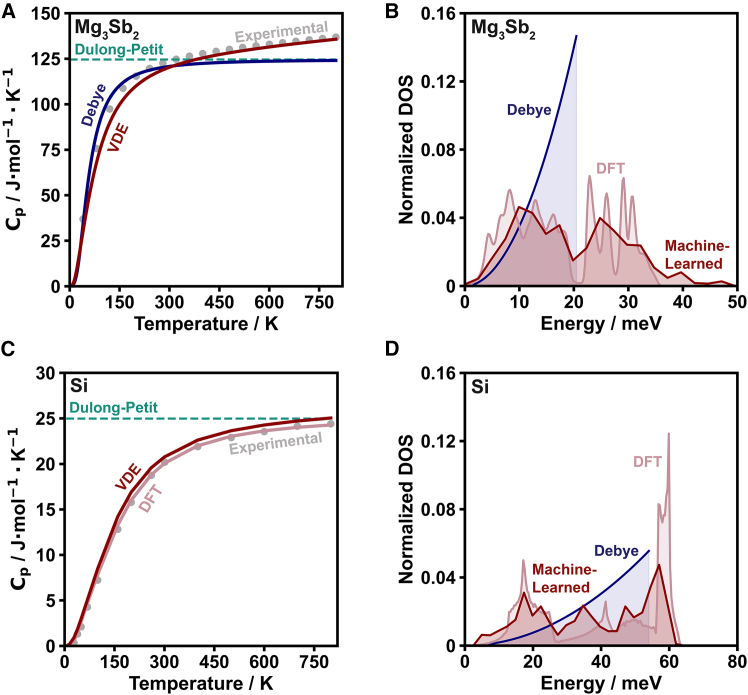
Temperature-dependent heat capacity and phonon density of states of ${\text{Mg}}_{\text{3}}{\text{Sb}}_{\text{2}}$ and Si (A) Heat capacity estimations of ${\text{Mg}}_{\text{3}}{\text{Sb}}_{\text{2}}$ as a function of temperature with the Dulong-Petit (teal dotted line), Debye (blue), and VDE (red) models compared with experimental values^24^ (gray points). (B) Normalized phonon density of states estimates as a function of phonon energy (E=ℏω) for ${\text{Mg}}_{\text{3}}{\text{Sb}}_{\text{2}}$ using the Debye model (blue), the ALIGNN algorithm from Gurunathan et al.^35^ (red), and using DFT with phonopy^49^^,^^50^ (pink), previously reported.^24^ (C) Heat capacity estimations of Si as a function of temperature with the Dulong-Petit (teal dotted line), DFT (pink), and VDE (red) models compared with experimental values^30^ (gray points). (D) Normalized phonon density of states estimates as a function of phonon energy for Si using the Debye model (blue), the ALIGNN algorithm from Gurunathan et al.^35^ (red), and using DFT with phonopy^49^^,^^50^ (pink).

The temperature-dependent heat capacity of ${\text{Mg}}_{\text{3}}{\text{Sb}}_{\text{2}}$ and Si was evaluated and compared with experimental results (Figure 4; Note S4). The compound ${\text{Mg}}_{\text{3}}{\text{Sb}}_{\text{2}}$ is an exceptional n-type thermoelectric material that is the first to truly rival ${\text{Bi}}_{\text{2}}{\text{Te}}_{\text{3}}$ in the low-mid temperature range (∼300–500 K)^47^ and consequently has received a lot of research attention over the past decade.^48^ Because the heat capacity of ${\text{Mg}}_{\text{3}}{\text{Sb}}_{\text{2}}$ has been theoretically and experimentally evaluated in detail previously,^24^ this comparison also serves as a validation of the VDE model.

With both ${\text{Mg}}_{\text{3}}{\text{Sb}}_{\text{2}}$ and Si, the VDE model has good agreement with experimental values. The case of ${\text{Mg}}_{\text{3}}{\text{Sb}}_{\text{2}}$ (Figures 4A and 4B) particularly exemplifies the importance of the dilation term. In this material, the experimentally observed heat capacity is higher than the Dulong-Petit value at temperatures above ∼300 K, which is not considered in models of $C_{V}$. When the dilation term is not included in the VDE model, it too underestimates the experimental values at high temperature (Note S5). In any case, the machine-learned phonon density of states estimation captures more spectral features than the Debye model (Figure 4B). In addition to using the VDE model for Si, heat capacity was also estimated using DFT-determined phonon density of states and thermal expansion coefficient in Equation 8 (Figures 4C and 4D; Note S3). While the DFT-based estimation of heat capacity is somewhat closer to experimental values, the VDE model using a machine-learned phonon density of states estimate and harmonic approximation of the thermal expansion coefficient is also in good agreement. Thus, although more accurate estimates of phonon density of states and material properties may be incorporated into heat capacity models (e.g., Equation 3), this study highlights the capability of incorporating machine-learned inputs into physics-based models to provide better heat capacity estimates than analytical models alone can provide.

**Figure 5:**
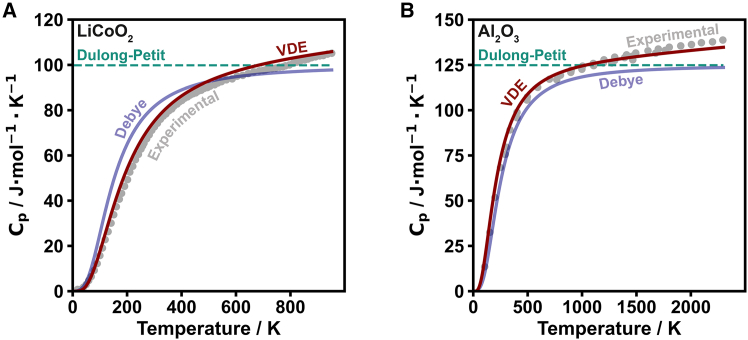
Heat capacity estimations of $\text{LiCo}O_{2}$ and ${\text{Al}}_{\text{2}}{\text{O}}_{\text{3}}$ (A) Heat capacity of $\text{LiCo}O_{2}$ as a function of temperature with the Dulong-Petit (teal dotted line), Debye (blue), and VDE (red) models compared with experimental values^53^^,^^54^ (gray points). (B) Heat capacity of ${\text{Al}}_{\text{2}}{\text{O}}_{\text{3}}$ as a function of temperature with the Dulong-Petit (teal dotted line), Debye (blue), and VDE (red) models compared with experimental values (gray points).^55^^,^^56^^,^^57^^,^^58^

To test its applicability across a wide temperature range, $\text{LiCo}O_{2}$, an archetype battery cathode material,^51^ and alumina (${\text{Al}}_{\text{2}}{\text{O}}_{\text{3}}$), a common high-temperature ceramic,^52^ were investigated (Figure 5; Note S4). For both materials, the VDE model more closely follows experimentally determined heat capacity values than the Debye model and maintains its accuracy even at the highest temperatures (above 2,000 K in the case of alumina). Although the Dulong-Petit and Debye models capture the high-temperature limit of $C_{V}$, the full contributions to $C_{p}$ are captured in the VDE model, displaying the strength of the VDE model in accounting for dilation contributions at high temperature. At low temperature, the more detailed vibrational density of states contributes to closer predictions of experimental heat capacity. This is especially important considering that $\text{LiCo}O_{2}$ is primarily used in batteries around room temperature where the heat capacity has a strong temperature dependence.

**Figure 6:**
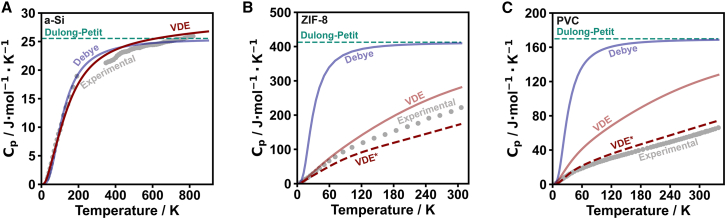
Heat capacity estimations of a-Si, ZIF-8, and PVC (A) Heat capacity of a-Si as a function of temperature with the Dulong-Petit (teal dotted line), Debye (blue), and VDE (red) models compared with experimental values^61^^,^^62^ (gray points). (B) Heat capacity of ZIF-8 as a function of temperature with the Dulong-Petit model (teal dotted line), Debye model (blue), VDE model using ALIGNN (pink), and VDE model using MACE (red dotted, Note S6). Models are compared with experimental values for ZIF-8^63^ (gray points). (C) Heat capacity of PVC as a function of temperature with the Dulong-Petit model (teal dotted line), Debye model (blue), VDE model using ALIGNN (pink), and VDE model using MACE (red dotted, Note S6). Models are compared with experimental values for PVC^64^ (gray points).

Because the ALIGNN model relies on the atomic structure to estimate the phonon density of states, it is appropriate to question what the effect of structural complexity may be on VDE model estimates. For this assessment, amorphous silicon (a-Si), a well-known MOF material known as ZIF-8,^59^ and the polymer polyvinyl chloride (PVC) were investigated (Figure 6; Notes S4 and S6). Given that the ALIGNN machine-learned phonon density of states estimation was trained on relatively small unit cell crystalline structures, it is not surprising that the VDE model closely follows experimental values of a material like $\text{LiCo}O_{2}$ (Figure 5A). Interestingly, however, a-Si (Figure 6A), which is far outside this training in terms of its number of atoms and non-crystalline nature,^60^ is still reasonably predicted by the VDE model, especially considering the discontinuity between the low-temperature and high-temperature data.^61^^,^^62^ In the cases of ZIF-8 and PVC, the heat capacity is somewhat overestimated by the VDE model compared with experimental values (Figures 6B and 6C) resulting from an overestimation of the low-frequency (<120 meV) vibrational density of states. Recognizing that there are vibrational modes in ZIF-8 and PVC at energies higher than 120 meV (Note S6), the apparent normalized density of states from the ALIGNN model is clearly overestimated. Still, the Debye and Dulong-Petit models fail drastically in describing the temperature dependence and magnitude of heat capacity in ZIF-8 and PVC up to ∼300 K.

The high-frequency vibrations in ZIF-8 are better handled by the MACE force field model, but given that the heat capacity is underestimated (see VDE∗ in Figure 6B), this indicates that the frequencies of the vibrational modes are overestimated on average (Note S6). For the case of PVC, however, the MACE model provides an exceptional estimation for the vibrational modes, providing a much-improved estimate for the heat capacity (see VDE∗ in Figure 6C). Thus, while the VDE model using the ALIGNN estimate of the phonon density of states provides the least complicated assessment of heat capacity, the use of machine-learned force fields can be used without much additional complexity.

While the 38 test materials (Figure 2) demonstrate that the VDE model performs well for structures within the scope of the ALIGNN model training, the specific example materials (Figures 5 and 6; Notes S4–S7) illustrate that the VDE model may even be applied to materials outside that scope. Further improvements may still be attained by developing machine-learned models for the phonon density of states that explicitly consider large unit cells and complex crystals, as well as amorphous materials. Nevertheless, the VDE model represents the possible advantage of incorporating machine-learned results into physics-based models instead of using machine learning alone^65^ and provides benchmarking for different machine-learned models. Further, the dilation term from phonon pressure theory excels in this analysis. As thermodynamic assessments and transport models for engineering devices are increasingly important for the development of next-generation technologies, accessible estimates of heat capacity play a foundational role.

## Heat capacity during phase transformations

Phase transformations are ubiquitous in engineering materials. Although many phase transformations are the result of a change in the thermodynamic free energy landscape with temperature or pressure, changes in elemental composition also initiate phase transformations. For example, battery materials used for the anode and cathode often undergo phase transformations during charge/discharge cycling due to changing local concentrations of the mobile ion (e.g., ${\text{Li}}^{+}$). Reported changes to the thermoelectric properties of materials undergoing phase transformations have led to controversial claims of increased thermoelectric figure of merit.^19^ Other specific phase transitions, such as spin-crossover in MOF materials^66^ or those in chalcogenide memory materials,^67^ are also gaining increasing attention with respect to the material’s thermal properties. More generally, characterizing and understanding the impact of phase transformations on thermal properties are foundational to engineering thermal management in modern materials systems.

**Figure 7:**
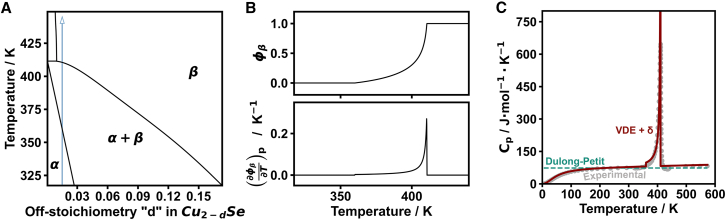
Heat capacity of $Cu_{2}$Se through a phase transition estimated using the VDE model and the equilibrium phase diagram (A) A portion of the Cu-Se phase diagram depicting the α→β phase transition of ${\text{Cu}}_{2-d}$Se.^69^^,^^70^^,^^71^ (B) The equilibrium phase fraction $\phi_{\beta }$ of the high-temperature β phase and its temperature derivative ${\left(\partial \phi_{\beta }/\partial T\right)}_{p}$ are shown as a function of temperature for ${\text{Cu}}_{\text{1.985}}$Se (corresponding to the blue arrow in A).^19^ (C) Heat capacity estimations of $Cu_{2}$Se with the Dulong-Petit model (teal dotted line) and the VDE model using ALIGNN with the added phase change contribution denoted by δ, where $\delta =\Delta H{\left(\partial \phi_{\beta }/\partial T\right)}_{p}$ (red). Models are compared with experimental values^72^^,^^73^^,^^74^ (gray points). The discontinuity in the VDE $C_{p}$ curve at the onset and conclusion of the phase transition is due to the numerical aspect of calculating ${\left(\partial \phi_{\beta }/\partial T\right)}_{p}$ without consideration of thermal broadening (Note S8). In effect, going from the α to α+β region happens in a stepwise manner, leading to the apparent discontinuity.

Phase transformations contribute to heat capacity when they store thermal energy by maintaining equilibrium in the system. The total heat capacity of a multi-phase system is the sum of the intrinsic heat capacity $C_{p\phi }$ of the phases present (e.g., Equation 3) and the contribution from the enthalpy of transformation ΔH as (Note S9)

(Equation 10) $$C_{p}=C_{p\phi }+\Delta H{\left(\frac{\partial \phi }{\partial T}\right)}_{p}$$

when changes in equilibrium (characterized by the order parameter ϕ) are able to respond “instantly” to changes in temperature. More generally, when ϕ does not respond instantly to changes in temperature, the relation^68^

(Equation 11) $$C_{p}=C_{p\phi }+\Delta H\frac{{\left(\partial \phi /\partial t\right)}_{p}}{{\left(\partial T/\partial t\right)}_{p}}$$

can be used, which considers the respective changes of ϕ and T with time t. Nevertheless, Equation 10 provides a valuable starting point, as ${\left(\partial \phi /\partial T\right)}_{p}$ can be estimated directly from the equilibrium phase diagram using the inverse lever rule (Figures 7A and 7B). Microscopically, the additional contribution to the heat capacity arises from the additional degree(s) of freedom facilitated by having a dynamic interface between the equilibrium phases. This is to say that thermal energy is stored in the fluctuations in ϕ at equilibrium.

For materials where phase transformation contributions to the heat capacity are observed experimentally, Equation 10 is reasonably expected to explain the temperature dependence of the heat capacity. Here, an estimation of the heat capacity of $Cu_{2}$Se from ∼2–575 K, through the α→β phase transition, was achieved using the VDE model (Equation 8) as $C_{p\phi }$ in Equation 10 (Figure 7C; Notes S4 and S9). The ${\left(\partial \phi /\partial T\right)}_{p}$ term was estimated using the phase diagram of $Cu_{2}$Se,^19^^,^^69^^,^^70^^,^^71^ and ΔH was considered to be temperature independent with a value of 30 J${\text{·g}}^{\text{−1}}$.^69^ Despite the additional complexity contributed by the phase transformation region, there is excellent agreement of the heat capacity estimation with experimental values across the full temperature range (Figure 7C).

It should be emphasized that an accurate characterization of the phase diagram (in the vicinity of the phase transformation of interest) is a requisite step for the characterization of the heat capacity using Equation 10. This is because the specific values of ${\left(\partial \phi /\partial T\right)}_{p}$ will depend on the exact composition and the phase diagram. The time considerations of Equation 11 suggest that the apparent heat capacity at a given temperature could be somewhat less than that predicted by Equation 10. For example, thermal lag introduced inherently by the heating/cooling rate of experimental characterization techniques may affect measured heat capacity values at a given temperature. Consequently, a detailed study of transformation kinetics (as a function of time and temperature) may be necessary to explain phase transformation contributions to heat capacity measurements in some materials. Previous efforts^75^^,^^76^ to characterize phase transformation contributions to heat capacity from a kinetics perspective underline the importance of transformation “speed” but seemingly overlook the fundamental importance of composition and equilibrium phase relations in describing the heat capacity of multi-phase systems.

In the thermodynamic relation between thermal conductivity and thermal diffusivity (Equation 1), it is the total heat capacity (including phase transformation contributions) that should be used. Thus, characterizing heat capacity through phase transformations is vital to assessing thermal conductivity in these temperature regions. Concurrently, understanding the timescales of transformation kinetics, as well as measurement rates and thermal lag, is necessary for proper analysis of heat capacity data. Especially as considerations of thermal management in electrochemical devices, like solid-state batteries, become increasingly prominent, the ability to characterize/estimate heat and temperature evolution throughout dynamic processes will be beneficial.

## Outlook

Accurate characterization of material thermal properties is a fundamental step toward systems-level design capabilities. The importance of heat capacity, both for defining the thermodynamic state of a material and for estimating thermal conductivity, means that it should be thoroughly studied in its own right. State-of-the-art thermodynamics modeling, such as CALPHAD, requires estimates of heat capacity for predicting thermodynamic phase relations. Combined with high-throughput first-principles structure calculations, the computational design of materials is expected to rapidly accelerate technological innovation. Accurate estimates of thermal conductivity, especially in complex systems undergoing phase transformations, will be essential to device design. For example, heat capacity estimations in the design of battery architectures and the optimization of charging rates could aid in the effort to mitigate material degradation and improve battery safety. Likewise, accurate estimations of thermoelectric performance are essential for identifying materials with promising real-world applications.

In totality, the VDE model provides a straightforward estimate for heat capacity at temperatures relevant for engineering design with DFT-level accuracy. Including the dilation term to $C_{p}$ using the thermal expansion coefficient determined from phonon pressure theory allows for a description of heat capacity based solely on the structural and elastic properties of a solid. Estimates fall within ∼7% of experimental values from 300 to 600 K and are at least 29% more accurate than the Debye model on average. When DFT-based phonon density of states inputs are used in the VDE model, estimates fall within ∼2% of experimental values. Thus, the VDE model serves as both a high-throughput metric for heat capacity values and as an excellent starting point for more detailed heat capacity analysis. By also demonstrating how to include the contribution from phase transformations into a heat capacity model, this work provides a hierarchical perspective for assessing the heat capacity of complex materials. Toward this end, future work to incorporate machine-learning results into physics-based models may lead to further improvements in estimations of heat capacity and other thermophysical properties needed for modern materials design.

## Data and code availability

The data and code necessary to reproduce the findings of this study are available on Github (https://github.com/AgneLab/Heat-Capacity) and in the supplemental information found online at https://doi.org/10.1016/j.joule.2025.102054.

## Acknowledgments

E.J.C. and M.T.A. would like to acknowledge Research Advanced Computing Services (RACS) at the University of Oregon for providing computing resources that have contributed to the research results reported within this publication (https://racs.uoregon.edu). J.B. and J.G. would like to acknowledge the Gauss Centre for Supercomputing e.V. (https://www.gauss-centre.eu) for funding workflow-related developments by providing generous computing time on the GCS Supercomputer SuperMUC-NG at Leibniz Supercomputing Centre (http://www.lrz.de) (project pn73da). J.G. was supported by MultiBonds ERC Grant (grant agreement no. 101161771) funded by the European Union. Views and opinions expressed are, however, those of the author(s) only and do not necessarily reflect those of the European Union or the European Research Council Executive Agency. Neither the European Union nor the granting authority can be held responsible for them.

## Author contributions

Conceptualization, M.T.A.; methodology, E.J.C. and M.T.A.; VDE model coding, E.J.C.; coding support, Z.M.J., R.G., J.G., and M.T.A.; writing – original draft, E.J.C. and M.T.A.; writing – review and editing, E.J.C. and all authors; compiling material information, E.J.C., Z.M.J., A.L., J.B., and M.T.A.; DFT and MACE calculations, J.B., E.J.C., and J.G.; additional resources, R.G., J.G., and M.T.A.; supervision, M.T.A..

## Declaration of interests

The authors declare no competing interests.

## Supplemental information

Document S1. Figures S1–S12, Tables S1–S9, and Notes S1–S10

Document S2. Article plus supplemental information

## References

[1] Meng Y., Pu J., Pei Q. (2021). Electrocaloric cooling over high device temperature span. Joule.

[2] Wang Y., Wu X., Yu M., Shen X., Wang S., Li H., Zhang Z., Liu W. (2024). Thermoelectric cyclic-thermal regulation: A new operational mode of thermoelectric materials with high energy efficiency. Joule.

[3] Clarke D.R., Oechsner M., Padture N.P. (2012). Thermal-barrier coatings for more efficient gas-turbine engines. MRS Bull..

[4] Snyder G.J. (2004). Application of the compatibility factor to the design of segmented and cascaded thermoelectric generators. Appl. Phys. Lett..

[5] Agne M.T., Böger T., Bernges T., Zeier W.G. (2022). Importance of thermal transport for the design of solid-state battery materials. PRX Energy.

[6] Feng X., Ren D., He X., Ouyang M. (2020). Mitigating thermal runaway of lithium-ion batteries. Joule.

[7] Rui X., Ren D., Liu X., Wang X., Wang K., Lu Y., Li L., Wang P., Zhu G., Mao Y. (2023). Distinct thermal runaway mechanisms of sulfide-based all-solid-state batteries. Energy Environ. Sci..

[8] Bates A.M., Preger Y., Torres-Castro L., Harrison K.L., Harris S.J., Hewson J. (2022). Are solid-state batteries safer than lithium-ion batteries?. Joule.

[9] Kong J., Chung S.W., Skadron K. (2012). Recent thermal management techniques for microprocessors. ACM Comput. Surv..

[10] Diaconu B.M., Cruceru M., Anghelescu L. (2023). A critical review on heat transfer enhancement techniques in latent heat storage systems based on phase change materials. passive and active techniques, system designs and optimization. J. Energy Storage.

[11] Schleich W.P., Ranade K.S., Anton C., Arndt M., Aspelmeyer M., Bayer M., Berg G., Calarco T., Fuchs H., Giacobino E. (2016). Quantum technology: from research to application. Appl. Phys. B.

[12] Salomão R., Oliveira K., Fernandes L., Tiba P., Prado U. (2022). Porous refractory ceramics for high-temperature thermal insulation - part 2: The technology behind energy saving. Interceram. – Int. Ceram. Rev..

[13] Lan D., Green M.A. (2022). Combatting temperature and reverse-bias challenges facing perovskite solar cells. Joule.

[14] Park S., Kim T., Yoon S., Koh C.W., Woo H.Y., Son H.J. (2020). Progress in materials, solution processes, and long-term stability for large-area organic photovoltaics. Adv. Mater..

[15] Rosen P.F., Dickson M.S., Calvin J.J., Ross N.L., Friščić T., Navrotsky A., Woodfield B.F. (2020). Thermodynamic evidence of structural transformations in CO2 -loaded metal–organic framework Zn(Melm)2 from heat capacity measurements. J. Am. Chem. Soc..

[16] Snyder G.J., Toberer E.S. (2008). Complex thermoelectric materials. Nat. Mater..

[17] Grimvall G. (1999).

[18] Aftab W., Usman A., Shi J., Yuan K., Qin M., Zou R. (2021). Phase change material-integrated latent heat storage systems for sustainable energy solutions. Energy Environ. Sci..

[19] Agne M.T., Voorhees P.W., Snyder G.J. (2019). Phase transformation contributions to heat capacity and impact on thermal diffusivity, thermal conductivity, and thermoelectric performance. Adv. Mater..

[20] Ritz E.T., Li S.J., Benedek N.A. (2019). Thermal expansion in insulating solids from first principles. J. Appl. Phys..

[21] Blaine R.L. (2018). In search of thermal effusivity reference materials. J. Therm. Anal. Calorim..

[22] Agne M.T., Barsoum M.W. (2016). Enthalpy of formation and thermodynamic parameters of the max phase v2alc. J. Alloys Compd..

[23] Olson G.B., Liu Z.K. (2023). Genomic materials design: Calculation of phase dynamics. Calphad.

[24] Agne M.T., Imasato K., Anand S., Lee K., Bux S.K., Zevalkink A., Rettie A.J.E., Chung D.Y., Kanatzidis M.G., Snyder G.J. (2018). Heat capacity of mg3sb2, mg3bi2, and their alloys at high temperature. Mater. Today Phys..

[25] Agne M.T., Hanus R., Snyder G.J. (2018). Minimum thermal conductivity in the context of diffuson -mediated thermal transport. Energy Environ. Sci..

[26] Bernges T., Peterlechner M., Wilde G., Agne M.T., Zeier W.G. (2023). Analytical model for two-channel phonon transport engineering. Mater. Today Phys..

[27] Chen Z., Zhang X., Lin S., Chen L., Pei Y. (2018). Rationalizing phonon dispersion for lattice thermal conductivity of solids. Natl. Sci. Rev..

[28] Moon J., Zella L., Lindsay L. (2024). Collective nature of phonon energies beyond harmonic oscillators. Comp. Mater. Today.

[29] Borup K.A., De Boor J., Wang H., Drymiotis F., Gascoin F., Shi X., Chen L., Fedorov M.I., Müller E., Iversen B.B., Snyder G.J. (2015). Measuring thermoelectric transport properties of materials. Energy Environ. Sci..

[30] Porter L.J., Yip S., Yamaguchi M., Kaburaki H., Tang M. (1997). Empirical bond-order potential description of thermodynamic properties of crystalline silicon. J. Appl. Phys..

[31] Kittel C. (2005).

[32] Ridier K., Zhang Y., Piedrahita-Bello M., Quintero C.M., Salmon L., Molnár G., Bergaud C., Bousseksou A. (2020). Heat capacity and thermal damping properties of spin-crossover molecules: A new look at an old topic. Adv. Mater..

[33] Batatia I., Benner P., Chiang Y., Elena A.M., Kovács D.P., Riebesell J., Advincula X.R., Asta M., Avaylon M., Baldwin W.J. (2024). A foundation model for atomistic materials chemistry. arXiv.

[34] Yang H., Hu C., Zhou Y., Liu X., Shi Y., Li J., Li G., Chen Z., Chen S., Zeni C. (2024). Mattersim: A deep learning atomistic model across elements, temperatures and pressures. arXiv.

[35] Gurunathan R., Choudhary K., Tavazza F. (2023). Rapid prediction of phonon structure and properties using the atomistic line graph neural network (alignn). Phys. Rev. Mater..

[36] Choudhary K., Garrity K.F., Reid A.C.E., DeCost B., Biacchi A.J., Hight Walker A.R., Trautt Z., Hattrick-Simpers J., Kusne A.G., Centrone A. (2020). The joint automated repository for various integrated simulations (jarvis) for data-driven materials design. Computational Materials.

[37] Choudhary K., Tavazza F. (2019). Convergence and machine learning predictions of monkhorst-pack k-points and plane-wave cut-off in high-throughput dft calculations. Comp. Mater. Sci..

[38] Chen Z., Andrejevic N., Smidt T., Ding Z., Xu Q., Chi Y.T., Nguyen Q.T., Alatas A., Kong J., Li M. (2021). Direct prediction of phonon density of states with euclidean neural networks. Adv. Sci. (Weinh).

[39] Okabe R., Chotrattanapituk A., Boonkird A., Andrejevic N., Fu X., Jaakkola T.S., Song Q., Nguyen T., Drucker N., Mu S. (2024). Virtual node graph neural network for full phonon prediction. Nat. Comput. Sci..

[40] Kong S., Ricci F., Guevarra D., Neaton J.B., Gomes C.P., Gregoire J.M. (2022). Density of states prediction for materials discovery via contrastive learning from probabilistic embeddings. Nat. Commun..

[41] Choudhary K., DeCost B., Major L., Butler K., Thiyagalingam J., Tavazza F. (2023). Unified graph neural network force-field for the periodic table: solid state applications. Digit. Discov..

[42] Grimme S., Antony J., Ehrlich S., Krieg H. (2010). A consistent and accurate ab initio parametrization of density functional dispersion correction (dft-d) for the 94 elements h-pu. J. Chem. Phys..

[43] Jain A., Ong S.P., Hautier G., Chen W., Richards W.D., Dacek S., Cholia S., Gunter D., Skinner D., Ceder G., Persson K.A. (2013). Commentary: The materials project: A materials genome approach to accelerating materials innovation. APL Mater..

[44] Agne M.T., Anand S., Snyder G.J. (2022). Inherent anharmonicity of harmonic solids. Research (Wash D. C).

[45] Chen Z., Liu W., Shan B., Pei Y. (2024). Analytical approach to structural chemistry origins of mechanical, acoustical and thermal properties. Natl. Sci. Rev..

[46] Allison, T.C. (2013). NIST-JANAF Thermochemical Tables. https://janaf.nist.gov/.

[47] Imasato K., Kang S.D., Ohno S., Snyder G.J. (2018). Band engineering in mg 3 sb 2 by alloying with mg 3 bi 2 for enhanced thermoelectric performance. Mater. Horiz..

[48] Imasato K., Wood M., Anand S., Kuo J.J., Snyder G.J. (2022). Understanding the high thermoelectric performance of mg 3 Sb 2 -Mg3 Bi 2 alloys. Adv. Energy Sustain. Res..

[49] Togo A. (2023). First-principles Phonon Calculations with Phonopy and Phono3py. J. Phys. Soc. Jpn..

[50] Togo A., Chaput L., Tadano T., Tanaka I. (2023). Implementation strategies in phonopy and phono3py. J. Phys. Condens. Matter.

[51] Liu Q., Su X., Lei D., Qin Y., Wen J., Guo F., Wu Y.A., Rong Y., Kou R., Xiao X. (2018). Approaching the capacity limit of lithium cobalt oxide in lithium ion batteries via lanthanum and aluminium doping. Nat. Energy.

[52] Wang X., Zhong Y., Hu Q. (2025). A review of al2o3-based eutectic ceramics for high-temperature structural materials. J. Mater. Sci. Technol..

[53] Gotcu-Freis P., Cupid D.M., Rohde M., Seifert H.J. (2015). New experimental heat capacity and enthalpy of formation of lithium cobalt oxide. J. Chem. Thermodyn..

[54] Kawaji H., Takematsu M., Tojo T., Atake T., Hirano A., Kanno R. (2002). Low temperature heat capacity and thermodynamic functions of licoo2. Journal of Thermal Analysis and Calorimetry.

[55] Huang L.F., Lu X.Z., Tennessen E., Rondinelli J.M. (2016). An efficient ab-initio quasiharmonic approach for the thermodynamics of solids. Comp. Mater. Sci..

[56] Munro R.G. (1997). Evaluated material properties for a sintered alpha-alumina. J. Am. Ceram. Soc..

[57] Schauer A. (1965). Thermal expansion, grueneisen parameter, and temperature dependence of lattice vibration frequencies of aluminum oxide. Can. J. Phys..

[58] Chase M. (1998).

[59] Lai Z. (2018). Development of zif-8 membranes: opportunities and challenges for commercial applications. Curr. Opin. Chem. Eng..

[60] Deringer V.L., Bernstein N., Bartók A.P., Cliffe M.J., Kerber R.N., Marbella L.E., Grey C.P., Elliott S.R., Csányi G. (2018). Realistic atomistic structure of amorphous silicon from machine-learning-driven molecular dynamics. J. Phys. Chem. Lett..

[61] Tsang K.H., Kui H.W., Chik K.P. (1993). Calorimetric studies of the heat capacity and relaxation of amorphous si prepared by electron beam evaporation. J. Appl. Phys..

[62] Queen D.R., Liu X., Karel J., Metcalf T.H., Hellman F. (2013). Excess specific heat in evaporated amorphous silicon. Phys. Rev. Lett..

[63] Rosen P.F., Calvin J.J., Dickson M.S., Katsenis A.D., Friščić T., Navrotsky A., Ross N.L., Kolesnikov A.I., Woodfield B.F. (2019). Heat capacity and thermodynamic functions of crystalline forms of the metal-organic framework zinc 2-methylimidazolate, zn(meim)2. J. Chem. Thermodyn..

[64] Chang S.S. (1977). Heat capacity and thermodynamic properties of poly(vinyl chloride). J. Res. Natl. Bur. Stand. (1977).

[65] Kauwe S.K., Graser J., Vazquez A., Sparks T.D. (2018). Machine learning prediction of heat capacity for solid inorganics. Integr. Mater. Manuf. Innov..

[66] Davenport A.M., Marshall C.R., Nishiguchi T., Kadota K., Andreeva A.B., Horike S., Brozek C.K. (2024). Size-dependent spin crossover and bond flexibility in metal–organic framework nanoparticles. J. Am. Chem. Soc..

[67] Zhang W., Mazzarello R., Wuttig M., Ma E. (2019). Designing crystallization in phase-change materials for universal memory and neuro-inspired computing. Nat. Rev. Mater..

[68] Prigogine I., Defay R. (1954).

[69] Kang S.D., Danilkin S.A., Aydemir U., Avdeev M., Studer A., Snyder G.J. (2016). Apparent critical phenomena in the superionic phase transition of cu 2- x se. New J. Phys..

[70] Ishikawa T., Miyatani S.y. (1977). Electronic and ionic conduction in Cu 2-δ Se, Cu 2-δ S and Cu 2-δ (se, s). J. Phys. Soc. Jpn..

[71] Vučić Z., Milat O., Horvatić V., Ogorelec Z. (1981). Composition-induced phase-transition splitting in cuprous selenide. Phys. Rev. B.

[72] Liu H., Shi X., Xu F., Zhang L., Zhang W., Chen L., Li Q., Uher C., Day T., Snyder G.J. (2012). Copper ion liquid-like thermoelectrics. Nat. Mater..

[73] Liu H., Yang J., Shi X., Danilkin S.A., Yu D., Wang C., Zhang W., Chen L. (2016). Reduction of thermal conductivity by low energy multi-einstein optic modes. J. Materiomics.

[74] Brown D.R., Heijl R., Borup K.A., Iversen B.B., Palmqvist A., Snyder G.J. (2016). Relating phase transition heat capacity to thermal conductivity and effusivity in cu 2 se. Physica Rapid. Research. Ltrs..

[75] Chen H., Yue Z., Ren D., Zeng H., Wei T., Zhao K., Yang R., Qiu P., Chen L., Shi X. (2019). Thermal conductivity during phase transitions. Adv. Mater..

[76] Qiu X., Qiu P., Yue Z., Chen H., Deng T., Xiao J., Ren D., Zhou Z., Chen L., Shi X. (2022). Phase transition behaviors and thermoelectric properties of CuAgTe1–xSex near 400 k. ACS Appl. Mater. Interfaces.

