# Supplementary material for: Heat capacity estimation of complex materials for energy technologies
Source: Joule. 2025 Aug 20;9(8):None. doi: 10.1016/j.joule.2025.102054 (PMC12369388; doi:10.1016/j.joule.2025.102054)
Supplement: Document S1. Figures S1–S12, Tables S1–S9, and Notes S1–S10 [file mmc1.pdf]

Joule, Volume *9*

Supplemental information

Heat capacity estimation of complex materials for energy technologies

Elana J. Cope, Joana Bustamante, Zöe M. Johnson, Alicia Lancaster, Ramya Gurunathan, Janine George, and Matthias T. Agne

Supplemental Note 1:

The VDE model code, related documents, and examples are provided at https://github.com/AgneLab/Heat-Capacity/, and example code can also be found below.

To run the VDE model, follow the following steps.

1. Ensure that Python is installed. Install alignn, pandas, numpy, matplotlib, scipy, pymatgen using pip installer.
2. Obtain a csv file with energy in meV and phonon density of states. This can be done by running the ALIGNN model code (https://github.com/usnistgov/alignn) or MACE (see below) on a provided CIF file using the following command:

run ALIGNN_DOS_Generate.py --model_name jv_pdos_alignn --file_format cif --file_path "./Compound.cif" --out_file "Compound.csv"

3. Obtain the following information for the compound: number of atoms per unit cell, density (gram per cubic cm), volume of cell (cubic Angstrom), bulk modulus (GPa), shear modulus (Gpa), molecular weight (g per mole). These can often be obtained from materials project[1] and loaded into the code via a csv file with inputs or using an API (https://api.materialsproject.org/docs). The number of atoms and the volume must be either both for the conventional cell or both for the primitive cell—do not mix and match.
4. If there is not a value for the electronic density of states at the Fermi level, just input 0 into the function for g_Ef. Otherwise, the electronic density of states at the Fermi level must be obtained using our function, which normalizes it into units of states per mole per cubic meter. The function takes the materials project ID and uses an API to obtain the full density of states, normalizes it, and finds the value at the Fermi level. Do not use the raw value of electronic density of states from Materials Project as the units will be incorrect.
5. Run the VDE model function with the energy and density of states values (e_raw_meV, g_raw), bulk modulus, shear modulus, density, molecular weight, and number of atoms from step 3 (B_vrh, G_vrh, Density, Molec_Wt, N_atom, V_cell). Follow step for the electronic density of states at the Fermi level (g_Ef), and define a temperature range (Temp).

To calculate the density of states using the MACE-MP-0-3b model, the phonon density of states with MACE-MP-0-3b[2] and D3 dispersion[3] correction can be computed. To perform the finite difference method, a Phonopy-based workflow[4,5] can be used, as implemented in atomate2.[6]

The code is shown below, with an example function that runs the model, saves the output into a csv, and plots the outputs.

```
######################################################################

import csv

import numpy as np

import matplotlib.pyplot as plt

from scipy.constants import hbar, k, e, pi

import pandas as pd
```

```
from matplotlib import rcParams, cm
from matplotlib.ticker import MaxNLocator
import os
from pymatgen.ext.matproj import MPRester
from collections import Counter
from pymatgen.core.periodic_table import Element  # To get atomic number
import time
import random

#figure settings
rcParams.update({'figure.autolayout': True}) #keeps the axis labels from getting cut off
# These commands modify default figure properties to make them more legible
plt.rcParams['axes.linewidth']    = 2.5
plt.rcParams['lines.linewidth']   = 1.5
plt.rcParams['font.sans-serif']   = 'Arial'
plt.rcParams['font.weight']       = 'bold'
plt.rcParams['axes.labelweight']  = 'bold'
plt.rcParams['axes.titleweight']  =    'bold'
plt.rcParams['axes.labelsize']    = 16
plt.rcParams['font.size'] = 18
plt.rcParams['axes.titlesize']    = 16
plt.rcParams['xtick.labelsize']   = 16
plt.rcParams['ytick.labelsize']  =  16
#plt.rcParams['axes.grid']        = True
plt.rcParams['xtick.major.size']  = 5
plt.rcParams['xtick.major.width'] = 2.5
plt.rcParams['ytick.major.size']  = 5
plt.rcParams['ytick.major.width'] = 2.5
plt.rcParams['lines.markersize']  = 12
plt.rcParams['xtick.minor.visible'] = False
plt.rcParams['ytick.minor.visible'] = False

#%%
```

```
def Cp_model(e_raw_meV, g_raw, B_vrh, G_vrh, Density, Molec_Wt, N_atom, V_cell, g_Ef, Temp):

    DF = pd.DataFrame(columns=['meV','DOS'])
    DF['meV']=pd.Series(e_raw_meV)
    DF['DOS']=pd.Series(g_raw)

    # delete all indices with negative energy using a boolean mask
    mask = DF.values[:, 0] >= 0 # energy in column 0
    pos_dataset = DF.values[mask]

    # replace any negative DOS values with 0
    pos_dataset[pos_dataset[:, 1] < 0, 1] = 0

    e_pos = pos_dataset[:,0]/6.241506363094e+21 # filtered energies converted from meV to J
    g_pos= pos_dataset[:,1] #filtered DOS (negative = 0)

    # normalize the DOS
    g_norm = g_pos/np.trapz(g_pos, e_pos) #normalized with e_pos (in J)

    #check to make sure DOS is normalized to 1. The printed value should equal 1 if the normalization
is correct.
    print(np.trapz(g_norm, e_pos))

    #handle division by zero
    epsilon = 1e-10 #constant to avoid dividing by 0
    Temp = np.array(Temp, dtype=float)
    Temp[Temp < epsilon] = epsilon

    Num_Density = N_atom/(V_cell * 1e-30) # number density in atom/(m^3)
    Vm = (Molec_Wt/1000)/(Density*1000)  # molar volume in m^3/mol

    #Compute Cv values:
    Cv_vib = [] #vibrational constant volume heat capcity defined
    for T in Temp:
```

```
        X = e_pos / (k * T)

        # Handle division by zero
        denominator = (np.exp(X) - 1)
        denominator[denominator < epsilon] = epsilon

        xx = (g_norm * (X**2) * np.exp(X)) / denominator**2 #stuff inside the integral
        integral_T = (k * 3 * Num_Density * np.trapz(xx, e_pos)) #compute the integral for every value
of T

        Cv_vib.append(integral_T)
    Cv_vib = np.array(Cv_vib)

    Cv_elec = ((pi**2) / 3) * (k**2) * g_Ef * Temp #electronic term for Cv
    Cv_total = Cv_vib + Cv_elec #total Cv from vib + elec

    #convert bulk and shear modulus values to Pa = (J/m^3)
    G_vrh_Pa = G_vrh*1e9
    B_vrh_Pa = B_vrh*1e9

    #Calculate thermal expansion coefficient and dilation terms
    Alpha =  (27 * Cv_total)/((20 * G_vrh_Pa) + (6 * B_vrh_Pa))
    Dilation = ((Alpha**2)*B_vrh_Pa*Temp)

    Cp = (Cv_total + Dilation)*Vm
    return Cp


def Plot(Temp, Cp, formula):
    #### plot Cp
    fig = plt.figure()
    ax = plt.subplot(1,1,1)
```

```
    plt.ylabel('$\mathrm{C_{p}}$ / J$\cdot$mol$^{-1}\cdot$K$^{-1}$')
    plt.xlabel('Temperature / K')

    #set range of axes
    xmin=0
    xmax=max(Temp)
    plt.xlim(xmin, xmax)
    ymin=0
    ymax=max(Cp)
    plt.ylim(ymin, ymax)

    #make square graph
    ax.set_aspect((xmax-xmin)/(ymax-ymin))
    #make five tick marks on each axis
    plt.gca().xaxis.set_major_locator(MaxNLocator(integer=True, nbins=6))
    plt.gca().yaxis.set_major_locator(MaxNLocator(integer=True, nbins=6))
    plt.plot(Temp, Cp, '-', color='darkred', linestyle='-', linewidth=3, label=f'{formula}')
    plt.legend()
    plt.savefig(f'Cp_vs_T_{formula}.png', dpi=800)
    plt.show()



def get_eDOS(material_id):
    API_KEY = "your API key here"
    # Initialize MPRester with your API key
    with MPRester(API_KEY) as mpr:

        dos = mpr.get_dos_by_material_id(material_id) #units= states / eV
        if dos is None:
            dos = np.nan
            dos.energies= np.nan #to avoid dividing by 0

        total_density = sum(dos.densities.values()) # Sum over both spins, if present, units = states / eV
```

```
    min_index = np.argmin(abs(dos.energies - dos.efermi))
    dos_energies = dos.energies * 1.602177e-19 #convert eV to J, units = J

    dos_integral = np.trapz(total_density, dos_energies)
    dos_norm = total_density/dos_integral # J^-1
    #check to make sure DOS is normalized to 1
    print('check if normalized (dos_norm/dos_energies:')
    print(np.trapz(dos_norm, dos_energies)) #units = J^-1 * J = unitless

    structure = mpr.get_structure_by_material_id(material_id)
    # Count the occurrences of each element in the unit cell
    element_counts = Counter(site.specie.symbol for site in structure)
    total_electrons = 0
    for element, count in element_counts.items():
        # Get the atomic number (total number of electrons) for the element
        atomic_number = Element(element).Z

        # Multiply the atomic number by the number of atoms in the unit cell
        total_electrons += atomic_number * count

    cell_volume = structure.volume #in cubic Angstrom
    charge_density = total_electrons/(cell_volume * 1e-30) #unit = states / m^3
    dos_norm_charge = charge_density * dos_norm # unit = states * J^-1 * m^-3
    eDOS_at_Fermi = dos_norm_charge[min_index] # unit = states * J^-1 * m^-3
    return eDOS_at_Fermi

##### Example usage
data_input = 'HeatCapacity_DataEntry_ForVDE.csv'
df = pd.read_csv(data_input, header=1)
for i, row in df.iterrows():
    # Initialize an empty list to store DataFrames for each material

    formula = df['Formula'][i]
    ID = df['Material_ID'][i]
```

```
N_atom = df['Num_Atoms'][i]
Density = df['Density_g_per_cubcm'][i]
V_cell = df['Vol_Cell_cubAngstrom'][i]
B_vrh = float(df['B_vrh_Gpa'][i])
G_vrh = float(df['G_vrh_Gpa'][i])
Molec_Wt = df['Molecular_Weight_g_per_mol'][i]

#For electronic density of states, either enter in value manually, use API, or enter '0'
#g_Ef = df['Elec_DOS_eF'][i]
#g_Ef = 0
g_Ef = get_eDOS(df['Material_ID'][i])



#get the DOS for that material
DOSdf = pd.read_csv(df['Formula'][i]+".csv", header=0)
e_raw_meV = DOSdf['meV']
g_raw = DOSdf['DOS']

Temps = np.arange(5, 600) #define temperatures that should be run

# Call the Cp_model function
Cp = Cp_model(e_raw_meV, g_raw, B_vrh, G_vrh, Density, Molec_Wt, N_atom, V_cell, g_Ef, Temps)

result_df = pd.DataFrame({
   'Temp':Temps,
   'Cp': Cp,
})

# Save the result_df DataFrame to a CSV file for the current material
result_filename = f'Cp_output_{formula}.csv'
result_df.to_csv(result_filename, index=False)

Plot(Temps, Cp, formula)
```

Supplemental Note 2:

Table S1. *Inputs used for the VDE model, obtained from the Materials Project Database.*[1]

Formula	*Material's Project ID*	*Number of atoms*	*Density ($g \cdot cm^{-3}$)*	*Unit cell volume ($Å^3$)*	*Electronic DOS at Fermi level ($states \cdot J^{-1} \cdot m^{-3}$)*	*VRH bulk modulus (GPa)*	*VRH shear modulus (GPa)*	*Molecular weight ($g \cdot mol^{-1}$)*
Si	mp-149	8	2.31	161.32	0	89	62	28.085
KI	mp-22898	8	3.1	355.63	0	11	6	166.003
NaI	mp-23268	8	3.73	266.76	0	15	8	149.894
KBr	mp-23251	8	2.76	286.07	0	13	7	119.00
NaBr	mp-22916	8	3.29	207.8	0	19	11	102.89
KCl	mp-23193	8	2	248.12	0	16	9	74.55
NaCl	mp-22862	8	2.22	174.5	0	24	14	58.44
KF	mp-463	8	2.58	149.62	0	30	17	58.096
NaF	mp-682	8	2.92	95.56	0	46	30	41.988
CaO	mp-2605	8	3.35	111.08	0	106	74	56.08
MgO	mp-1265	8	3.63	73.77	0	151	119	40.305
TiC	mp-631	8	4.89	81.26	7.52E+46	251	172	63.91
CsCl	mp-22865	2	3.93	71.15	0	15	7	168.36
Fe	mp-13	2	7.9	23.47	4.54E+47	207	68	55.845
TiO2	mp-2657	6	4.24	62.61	0	209	107	79.866
CrN	mp-2132	8	5.96	73.61	2.90E+47	102	32	66.003
$MgAl_2O_3$	mp-3536	56	3.58	527.59	0	183	98	142.27
$AlLiO_2$	mp-8001	12	3.44	95.39	0	143	97	65.92
Al_2BeO_4	mp-3081	28	3.71	227.11	0	215	147	126.973
B_2Ti	mp-1145	3	4.51	25.6	9.63E+46	254	257	69.489
$CMgO_3$	mp-5348	30	3.02	277.82	0	103	56	84.3139
B_2Mg	mp-763	3	2.67	28.6	1.03E+47	147	114	45.93
Co	mp-102	4	9.03	43.37	1.22E+47	201	120	58.933
LiF	mp-1138	8	2.53	68.09	0	70	50	25.99
MoS_2	mp-1434	9	4.67	170.76	0	25	16	160.07
FeS_2*-pyrite*	mp-226	12	5.07	157.19	0	146	135	119.98
PbI_2	mp-567503	6	5.7	268.73	0	1	2	460.809
Li_2O_3Ti	mp-2931	48	3.46	420.99	0	110	72	109.76
Li_2O	mp-1960	12	1.97	100.79	0	79	68	29.9
Li_3N	mp-2251	4	1.33	43.5	0	52	34	34.83
SiO_2 *- quartz*	mp-6930	9	2.63	113.71	0	34	46	60.08
$ZnSO_4$	mp-5126	24	3.93	272.6	0	71	33	161.47
C_3Al_4	mp-1591	21	2.99	239.71	0	159	113	143.96
Ni	mp-23	4	9.29	41.97	1.60E+47	174	92	58.6934
Mg_2O_4Si	mp-2895	28	3.24	288.81	0	119	75	140.69
LiCl	mp-22905	8	2.14	131.43	0	32	21	42.47
Al_2O_5Si*-Andalusite*	mp-4753	32	3.16	340.97	0	143	89	162.043
Hf	mp-103	2	13.31	44.53	7.06E+47	108	57	178.49

Table S2. *Model outputs at 100K. Outputs for vibrational, electronic, dilation terms and total heat capacity from the VDE model are shown, as well as Dulong-Petit (DP) model outputs, Debye model outputs, and experimental[7] heat capacity values for temperature = 100K. All values are reported in* $J \cdot mol^{-1} \cdot K^{-1}$.

Formula	$VDE\ C_V{}^{vib}$	$VDE\ C_V{}^{elec}$	$VDE\ C_V{}^{total}$	*VDE Dilation*	*VDE* C_p	*DP* C_p	*Debye* C_p	*Exp.* C_p
Si	8.55	0	8.55	0.01	8.56	24.97	6.07	7.27
KI	42.18	0	42.18	0.77	42.95	49.89	45.92	45
NaI	40.17	0	40.17	0.7	40.87	49.92	44.67	43.03
KBr	41.73	0	41.73	0.81	42.53	49.94	44.08	43.15
NaBr	38.88	0	38.88	0.6	39.48	49.87	40.84	40.17
KCl	39.63	0	39.63	0.65	40.28	49.78	39.27	39.2
NaCl	35.15	0	35.15	0.46	35.61	49.99	33.36	34.93
KF	31.56	0	31.56	0.36	31.92	49.87	31.39	31.61
NaF	3.34	0	3.34	0	3.34	49.86	20.71	22.84
CaO	18.44	0	18.44	0.04	18.48	49.94	11.47	14.72
MgO	10.35	0	10.35	0.01	10.36	49.87	5.12	7.8
TiC	12.61	0.06	12.67	0.01	12.68	53.29	5.37	7.34
CsCl	40.9	0	40.9	0.81	41.71	49.88	45.62	44.09
Fe	21.34	0.2	21.54	0.15	21.68	24.95	11.39	12.1
TiO2	16.09	0	16.09	0.02	16.11	74.77	11.64	18.5
CrN	16.78	0.2	16.98	0.12	17.1	49.85	27.32	9.99
$MgAl_2O_3$	24.95	0	24.95	0.02	24.97	174.71	22.59	22.67
$AlLiO_2$	16.47	0	16.47	0.02	16.49	99.85	10.87	17.97
Al_2BeO_4	21.42	0	21.42	0.01	21.43	174.77	12.11	16.37
B_2Ti	16.85	0.09	16.94	0.01	16.95	74.79	3.31	7.52
$CMgO_3$	30.65	0	30.65	0.08	30.74	124.87	25.5	24.68
B_2Mg	16.2	0.11	16.31	0.02	16.33	74.74	5.48	11.17
Co	15.43	0.05	15.48	0.04	15.52	24.93	7.95	13.91
LiF	17.18	0	17.18	0.07	17.26	49.99	9.04	12.82
MoS_2	29.09	0	29.09	0.2	29.3	74.83	58.36	24.05
FeS_2*-pyrite*	30.53	0	30.53	0.03	30.57	74.83	13.57	18.71
PbI_2	65.92	0	65.92	1.85	67.77	74.76	73.71	69.79
Li_2O_3Ti	31.7	0	31.7	0.06	31.76	149.81	25.96	31.14
Li_2O	12.33	0	12.33	0.02	12.34	74.85	6.62	10.45
Li_3N	21.82	0	21.82	0.07	21.89	99.74	16.03	22.2
SiO_2 *- quartz*	13.84	0	13.84	0.02	13.86	74.89	21.17	15.69
$ZnSO_4$	47.06	0	47.06	0.24	47.3	149.83	69.14	47.59
C_3Al_4	33.44	0	33.44	0.03	33.47	174.71	17.8	29.7
Ni	15.86	0.06	15.92	0.06	15.98	24.94	9.91	13.63
Mg_2O_4Si	37.77	0	37.77	0.06	37.83	174.37	30.2	32.32
LiCl	30.15	0	30.15	0.29	30.44	50.03	24.7	36.31
Al_2O_5Si*- Andalusite*	32.61	0	32.61	0.03	32.64	199.33	27.86	30.32
Hf	21.92	0.59	22.51	0.09	22.61	24.95	18.86	20.58

Table S3. *Model outputs at 200K. Outputs for vibrational, electronic, dilation terms and total heat capacity from the VDE model are shown, as well as Dulong-Petit (DP) model outputs, Debye model outputs, and experimental[7] heat capacity values for temperature = 200K. All values are reported in* $J \cdot mol^{-1} \cdot K^{-1}$.

Formula	$VDE\ C_V{}^{vib}$	$VDE\ C_V{}^{elec}$	$VDE\ C_V{}^{total}$	VDE Dilation	$VDE\ C_p$	$DP\ C_p$	$Debye\ C_p$	$Exp.\ C_p$
Si	16.8	0	16.8	0.1	16.89	24.97	16	15.64
KI	46.4	0	46.4	1.86	48.26	49.89	48.86	50.52
NaI	46.12	0	46.12	1.85	47.97	49.92	48.53	49.85
KBr	46.8	0	46.8	2.03	48.83	49.94	48.38	49.87
NaBr	45.16	0	45.16	1.62	46.78	49.87	47.36	48.74
KCl	46.54	0	46.54	1.78	48.32	49.78	46.81	48.44
NaCl	45.1	0	45.1	1.5	46.6	49.99	44.87	46.87
KF	43.29	0	43.29	1.35	44.64	49.87	44.03	45.19
NaF	12.5	0	12.5	0.1	12.6	49.86	38.71	40.81
CaO	36.65	0	36.65	0.28	36.92	49.94	31.28	33.68
MgO	28.58	0	28.58	0.15	28.73	49.87	21.67	26.68
TiC	29.69	0.12	29.81	0.1	29.92	53.29	22.95	14.92
CsCl	46.28	0	46.28	2.07	48.35	49.88	48.76	50.13
Fe	23.71	0.4	24.11	0.37	24.48	24.95	19.97	21.59
TiO2	41.14	0	41.14	0.24	41.38	74.77	39.73	42.01
CrN	33.74	0.4	34.14	1	35.14	49.85	42.24	29.13
$MgAl_2O_3$	82.35	0	82.35	0.49	82.83	174.71	85.18	78.07
$AlLiO_2$	49.74	0	49.74	0.34	50.09	99.85	44.72	48
Al_2BeO_4	75.47	0	75.47	0.29	75.76	174.77	61.16	66.43
B_2Ti	37.7	0.19	37.88	0.08	37.96	74.79	19.71	28.13
$CMgO_3$	66.89	0	66.89	0.8	67.69	124.87	74.59	57.57
B_2Mg	36.83	0.22	37.05	0.17	37.22	74.74	27.02	34.39
Co	21.62	0.1	21.72	0.16	21.88	24.93	17.68	22.23
LiF	35.31	0	35.31	0.61	35.93	49.99	28.37	32.8
MoS_2	54.88	0	54.88	1.45	56.33	74.83	70.14	51.84
FeS_2-pyrite	53.45	0	53.45	0.2	53.65	74.83	42.52	49.06
PbI_2	70.03	0	70.03	4.18	74.21	74.76	74.5	75.4
Li_2O_3Ti	83.45	0	83.45	0.8	84.25	149.81	83.47	82.66
Li_2O	38.67	0	38.67	0.34	39.01	74.85	30.05	37.2
Li_3N	56.9	0	56.9	0.95	57.85	99.74	53.77	55.42
SiO_2 - quartz	36.33	0	36.33	0.23	36.56	74.89	50.81	32.64
$ZnSO_4$	86.9	0	86.9	1.61	88.51	149.83	120.36	78.05
C_3Al_4	89.55	0	89.55	0.37	89.92	174.71	75.63	82.1
Ni	21.41	0.13	21.53	0.22	21.76	24.94	19.08	22.47
Mg_2O_4Si	91.65	0	91.65	0.68	92.34	174.37	97.14	86.14
LiCl	42.65	0	42.65	1.14	43.79	50.03	41.06	43.35
Al_2O_5Si- Andalusite	94.65	0	94.65	0.52	95.18	199.33	100.81	84.61
Hf	23.76	1.19	24.95	0.23	25.18	24.95	23.19	24.42

Table S4. *Model outputs at 300K. Outputs for vibrational, electronic, dilation terms and total heat capacity from the VDE model are shown, as well as Dulong-Petit (DP) model outputs, Debye model outputs, and experimental[7] heat capacity values for temperature = 300K. All values are reported in* $J \cdot mol^{-1} \cdot K^{-1}$.

Formula	VDE C_V^{vib}	VDE C_V^{elec}	VDE C_V^{total}	VDE Dilation	VDE C_p	DP C_p	Debye C_p	Exp. C_p
Si	20.55	0	20.55	0.21	20.76	24.97	20.27	20.05
KI	47.58	0	47.58	2.94	50.52	49.89	49.43	52.81
NaI	47.71	0	47.71	2.97	50.69	49.92	49.29	52.26
KBr	48.16	0	48.16	3.22	51.38	49.94	49.24	52.3
NaBr	47.02	0	47.02	2.63	49.65	49.87	48.73	51.44
KCl	48.14	0	48.14	2.86	50.99	49.78	48.43	51.33
NaCl	47.6	0	47.6	2.51	50.11	49.99	47.62	50.54
KF	46.53	0	46.53	2.33	48.87	49.87	47.14	49.02
NaF	23.1	0	23.1	0.49	23.59	49.86	44.41	46.92
CaO	43.06	0	43.06	0.57	43.63	49.94	40.09	42.24
MgO	37.92	0	37.92	0.4	38.32	49.87	33.16	37.24
TiC	39.39	0.18	39.58	0.27	39.85	53.29	35.26	33.94
CsCl	47.82	0	47.82	3.31	51.12	49.88	49.38	52.47
Fe	24.3	0.6	24.9	0.59	25.49	24.95	22.54	25.14
TiO2	55.18	0	55.18	0.64	55.82	74.77	55.25	55.29
CrN	40.92	0.6	41.52	2.22	43.73	49.85	46.25	51.39
$MgAl_2O_3$	119.37	0	119.37	1.53	120.91	174.71	123.46	116.53
$AlLiO_2$	70.22	0	70.22	1.03	71.25	99.85	67.46	68.13
Al_2BeO_4	113.14	0	113.14	0.98	114.12	174.77	103.16	105.94
B_2Ti	51.33	0.28	51.61	0.22	51.83	74.79	37.45	44.52
$CMgO_3$	88.32	0	88.32	2.08	90.4	124.87	97.91	76.5
B_2Mg	50.51	0.33	50.85	0.48	51.33	74.74	44.93	47.87
Co	23.26	0.15	23.41	0.28	23.69	24.93	21.26	23.98
LiF	42.36	0	42.36	1.33	43.68	49.99	38.2	41.94
MoS_2	64.47	0	64.47	3	67.47	74.83	72.69	63.68
FeS_2-pyrite	62.95	0	62.95	0.42	63.36	74.83	57.22	62.29
PbI_2	71.32	0	71.32	6.5	77.82	74.76	74.65	77.61
Li_2O_3Ti	111.93	0	111.93	2.15	114.08	149.81	113.41	110.75
Li_2O	53.94	0	53.94	0.98	54.93	74.85	47.68	54.38
Li_3N	75.38	0	75.38	2.51	77.89	99.74	74.25	75.54
SiO_2 - quartz	50.65	0	50.65	0.66	51.31	74.89	62.53	44.77
$ZnSO_4$	110.19	0	110.19	3.89	114.09	149.83	135.59	99.37
C_3Al_4	123.81	0	123.81	1.07	124.88	174.71	115.92	117.3
Ni	23.09	0.19	23.28	0.39	23.67	24.94	22.06	26.02
Mg_2O_4Si	123.33	0	123.33	1.86	125.19	174.37	131.99	119.15
LiCl	46.27	0	46.27	2.02	48.28	50.03	45.73	48.1
Al_2O_5Si- Andalusite	134.92	0	134.92	1.6	136.51	199.33	143.58	123.32
Hf	24.28	1.78	26.06	0.37	26.43	24.95	24.14	25.71

Table S5. *Model outputs at 400K. Outputs for vibrational, electronic, dilation terms and total heat capacity from the VDE model are shown, as well as Dulong-Petit (DP) model outputs, Debye model outputs, and experimental[7] heat capacity values for temperature = 400K. All values are reported in* $J \cdot mol^{-1} \cdot K^{-1}$.

Formula	$VDE\ C_V{}^{vib}$	$VDE\ C_V{}^{elec}$	$VDE\ C_V{}^{total}$	VDE Dilation	$VDE\ C_p$	$DP\ C_p$	$Debye\ C_p$	$Exp.\ C_p$
Si	22.28	0	22.28	0.34	22.62	24.97	22.16	22.14
KI	48.11	0	48.11	4.01	52.12	49.89	49.63	53.93
NaI	48.39	0	48.39	4.08	52.46	49.92	49.57	53.81
KBr	48.74	0	48.74	4.39	53.14	49.94	49.55	53.81
NaBr	47.93	0	47.93	3.65	51.57	49.87	49.23	53.45
KCl	48.74	0	48.74	3.9	52.65	49.78	49.01	53.14
NaCl	48.58	0	48.58	3.49	52.07	49.99	48.64	52.35
KF	47.85	0	47.85	3.29	51.14	49.87	48.31	51.05
NaF	30.98	0	30.98	1.17	32.14	49.86	46.68	49.6
CaO	45.8	0	45.8	0.87	46.67	49.94	44.02	46.63
MgO	42.46	0	42.46	0.66	43.12	49.87	39.3	42.56
TiC	44.48	0.25	44.73	0.46	45.19	53.29	41.87	40.69
CsCl	48.5	0	48.5	4.54	53.04	49.88	49.6	54.69
Fe	24.54	0.81	25.34	0.81	26.15	24.95	23.55	27.39
TiO2	62.39	0	62.39	1.09	63.49	74.77	62.76	62.84
CrN	44.25	0.81	45.05	3.48	48.53	49.85	47.78	49.08
$MgAl_2O_3$	139.32	0	139.32	2.79	142.11	174.71	142.81	137.99
$AlLiO_2$	81.04	0	81.04	1.83	82.87	99.85	79.44	81.55
Al_2BeO_4	134.57	0	134.57	1.85	136.43	174.77	128.04	130.28
B_2Ti	59.29	0.37	59.66	0.39	60.04	74.79	49.42	54.89
$CMgO_3$	100.64	0	100.64	3.61	104.24	124.87	108.57	89.86
B_2Mg	58.61	0.44	59.05	0.87	59.92	74.74	55.38	54.39
Co	23.91	0.2	24.11	0.4	24.51	24.93	22.77	24.83
LiF	45.4	0	45.4	2.03	47.43	49.99	42.81	46.54
MoS_2	68.58	0	68.58	4.53	73.11	74.83	73.62	68.91
FeS_2-pyrite	67.38	0	67.38	0.64	68.02	74.83	64.1	68.85
PbI_2	71.93	0	71.93	8.82	80.75	74.76	74.7	78.86
Li_2O_3Ti	126.16	0	126.16	3.65	129.81	149.81	127.58	127.36
Li_2O	61.73	0	61.73	1.72	63.45	74.85	57.46	64
Li_3N	84.54	0	84.54	4.2	88.74	99.74	84.1	87.1
SiO_2 - quartz	58.88	0	58.88	1.19	60.07	74.89	67.55	53.43
$ZnSO_4$	123.53	0	123.53	6.52	130.05	149.83	141.57	116.02
C_3Al_4	142.12	0	142.12	1.88	144	174.71	137.49	138.51
Ni	23.81	0.25	24.07	0.56	24.62	24.94	23.26	28.49
Mg_2O_4Si	140.93	0	140.93	3.24	144.17	174.37	148.49	137.56
LiCl	47.77	0	47.77	2.86	50.63	50.03	47.55	50.97
Al_2O_5Si- Andalusite	157.44	0	157.44	2.9	160.33	199.33	164.81	149.57
Hf	24.51	2.37	26.88	0.53	27.42	24.95	24.49	26.75

Table S6. *Model outputs at 500K. Outputs for vibrational, electronic, dilation terms and total heat capacity from the VDE model are shown, as well as Dulong-Petit (DP) model outputs, Debye model outputs, and experimental*[7] *heat capacity values for temperature = 500K. All values are reported in* $J \cdot mol^{-1} \cdot K^{-1}$.

Formula	$VDE\ C_V{}^{vib}$	$VDE\ C_V{}^{elec}$	$VDE\ C_V{}^{total}$	*VDE Dilation*	*VDE* C_p	*DP* C_p	*Debye* C_p	*Exp.* C_p
Si	23.18	0	23.18	0.46	23.64	24.97	23.12	23.33
KI	48.4	0	48.4	5.07	53.47	49.89	49.73	55.36
NaI	48.73	0	48.73	5.17	53.9	49.92	49.69	55.02
KBr	49.04	0	49.04	5.56	54.6	49.94	49.69	55.25
NaBr	48.45	0	48.45	4.66	53.11	49.87	49.46	54.85
KCl	49.03	0	49.03	4.94	53.97	49.78	49.29	54.75
NaCl	49.06	0	49.06	4.45	53.51	49.99	49.12	53.94
KF	48.51	0	48.51	4.23	52.74	49.87	48.86	52.72
NaF	36.21	0	36.21	1.99	38.2	49.86	47.79	51.26
CaO	47.2	0	47.2	1.15	48.35	49.94	46.03	48.98
MgO	44.88	0	44.88	0.92	45.8	49.87	42.7	45.54
TiC	47.3	0.31	47.61	0.65	48.26	53.29	45.54	45.17
CsCl	48.87	0	48.87	5.76	54.63	49.88	49.7	56.9
Fe	24.66	1.01	25.67	1.04	26.7	24.95	24.04	29.7
TiO2	66.36	0	66.36	1.55	67.91	74.77	66.74	67.2
CrN	46	1.01	47.01	4.73	51.74	49.85	48.51	49.75
$MgAl_2O_3$	150.51	0	150.51	4.07	154.58	174.71	153.26	149.83
$AlLiO_2$	87.05	0	87.05	2.63	89.68	99.85	86.04	88.41
Al_2BeO_4	146.99	0	146.99	2.77	149.76	174.77	142.54	145.25
B_2Ti	63.98	0.47	64.45	0.56	65.01	74.79	56.89	61.67
$CMgO_3$	107.93	0	107.93	5.19	113.12	124.87	114.07	100.06
B_2Mg	63.44	0.56	64	1.28	65.27	74.74	61.42	58.41
Co	24.23	0.25	24.48	0.52	24.99	24.93	23.51	26.53
LiF	46.94	0	46.94	2.71	49.66	49.99	45.21	49.33
MoS_2	70.67	0	70.67	6.01	76.68	74.83	74.05	71.76
FeS_2*-pyrite*	69.73	0	69.73	0.86	70.58	74.83	67.69	72.04
PbI_2	72.28	0	72.28	11.13	83.41	74.76	74.72	80.39
Li_2O_3Ti	133.85	0	133.85	5.13	138.99	149.81	135.01	135.65
Li_2O	65.99	0	65.99	2.46	68.44	74.85	62.98	69.58
Li_3N	89.48	0	89.48	5.89	95.36	99.74	89.3	96.94
SiO_2 *- quartz*	63.73	0	63.73	1.74	65.48	74.89	70.07	59.64
$ZnSO_4$	131.42	0	131.42	9.22	140.64	149.83	144.47	131.21
C_3Al_4	152.35	0	152.35	2.7	155.06	174.71	149.45	150.89
Ni	24.19	0.32	24.5	0.72	25.23	24.94	23.84	31.05
Mg_2O_4Si	151.16	0	151.16	4.66	155.82	174.37	157.13	148.7
LiCl	48.53	0	48.53	3.7	52.22	50.03	48.42	53.34
Al_2O_5Si*- Andalusite*	170.42	0	170.42	4.24	174.66	199.33	176.18	165.37
Hf	24.63	2.97	27.6	0.7	28.3	24.95	24.65	27.69

Table S7. *Model outputs at 600K. Outputs for vibrational, electronic, dilation terms and total heat capacity from the VDE model are shown, as well as Dulong-Petit (DP) model outputs, Debye model outputs, and experimental*[7] *heat capacity values for temperature = 600K. All values are reported in* $J \cdot mol^{-1} \cdot K^{-1}$.

Formula	$VDE\ C_V^{vib}$	$VDE\ C_V^{elec}$	$VDE\ C_V^{total}$	VDE Dilation	$VDE\ C_p$	$DP\ C_p$	$Debye\ C_p$	$Exp.\ C_p$
Si	23.7	0	23.7	0.57	24.27	24.97	23.66	24.15
KI	48.57	0	48.57	6.13	54.69	49.89	49.78	57.3
NaI	48.93	0	48.93	6.25	55.18	49.92	49.76	56.23
KBr	49.22	0	49.22	6.72	55.94	49.94	49.77	56.36
NaBr	48.77	0	48.77	5.67	54.44	49.87	49.58	56.07
KCl	49.19	0	49.19	5.96	55.16	49.78	49.44	56.32
NaCl	49.34	0	49.34	5.4	54.74	49.99	49.38	55.48
KF	48.89	0	48.89	5.15	54.04	49.87	49.17	54.27
NaF	39.67	0	39.67	2.87	42.54	49.86	48.41	52.68
CaO	48	0	48	1.43	49.42	49.94	47.18	50.48
MgO	46.3	0	46.3	1.18	47.48	49.87	44.73	47.43
TiC	48.99	0.37	49.36	0.84	50.19	53.29	47.73	47.65
CsCl	49.09	0	49.09	6.98	56.06	49.88	49.75	59.1
Fe	24.73	1.21	25.94	1.27	27.21	24.95	24.32	32.05
TiO2	68.73	0	68.73	1.99	70.72	74.77	69.06	69.93
CrN	47.03	1.21	48.23	5.98	54.21	49.85	48.92	50.42
$MgAl_2O_3$	157.23	0	157.23	5.33	162.56	174.71	159.4	157.89
$AlLiO_2$	90.65	0	90.65	3.43	94.07	99.85	89.95	92.69
Al_2BeO_4	154.61	0	154.61	3.67	158.28	174.77	151.42	154.97
B_2Ti	66.89	0.56	67.45	0.74	68.19	74.79	61.64	66.17
$CMgO_3$	112.47	0	112.47	6.76	119.23	124.87	117.22	109.04
B_2Mg	66.46	0.67	67.13	1.68	68.81	74.74	65.1	61.38
Co	24.41	0.3	24.71	0.63	25.34	24.93	23.94	28.2
LiF	47.82	0	47.82	3.38	51.2	49.99	46.6	51.59
MoS_2	71.85	0	71.85	7.46	79.31	74.83	74.29	73.6
FeS_2-pyrite	71.1	0	71.1	1.07	72.17	74.83	69.77	74.31
PbI_2	72.48	0	72.48	13.43	85.92	74.76	74.74	83.71
Li_2O_3Ti	138.39	0	138.39	6.59	144.97	149.81	139.3	141.4
Li_2O	68.5	0	68.5	3.18	71.68	74.85	66.3	73.82
Li_3N	92.38	0	92.38	7.53	99.92	99.74	92.32	106.43
SiO_2 - quartz	66.74	0	66.74	2.3	69.04	74.89	71.49	64.42
$ZnSO_4$	136.33	0	136.33	11.91	148.24	149.83	146.08	137.44
C_3Al_4	158.49	0	158.49	3.51	162.01	174.71	156.58	159.2
Ni	24.4	0.38	24.78	0.89	25.67	24.94	24.17	34.85
Mg_2O_4Si	157.47	0	157.47	6.06	163.54	174.37	162.13	156.43
LiCl	48.96	0	48.96	4.51	53.48	50.03	48.91	55.59
Al_2O_5Si- Andalusite	178.36	0	178.36	5.58	183.93	199.33	182.83	174.52
Hf	24.71	3.56	28.27	0.88	29.15	24.95	24.74	28.58

Heat capacity estimations and their comparisons to experimental values for the 38 materials (input parameters shown in Table S1) using the Dulong-Petit, Debye, and VDE models at 100, 200, 300, 400, 500, and 600 K are shown in Figures S1 and S2. Model outputs at higher temperatures generally had better agreement with experimental values. Across all temperatures, the VDE model had lower mean percent error values than the Debye and Dulong-petit models.

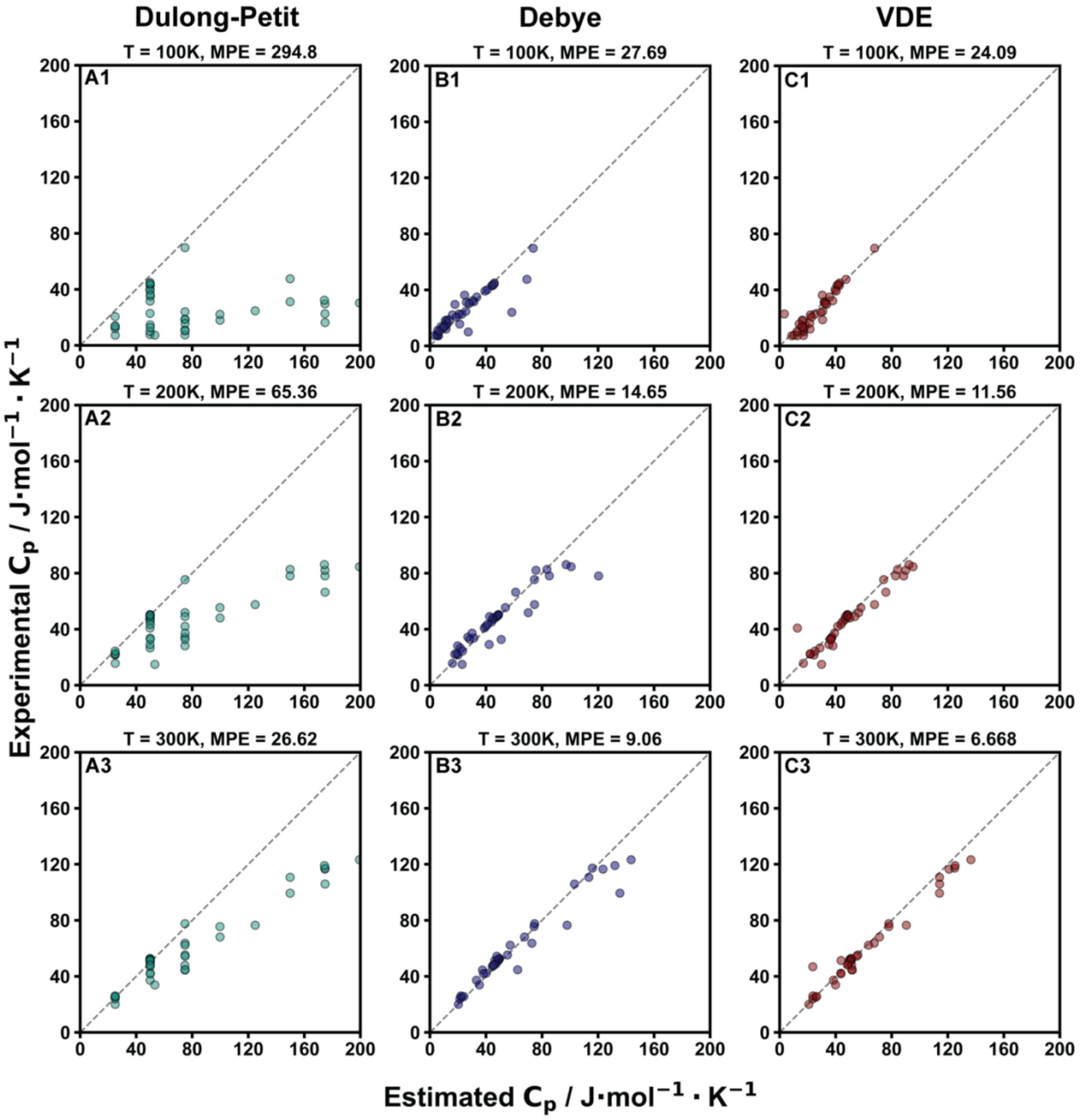

Figure S1. *Comparison of heat capacity estimates with experimental values for 38 diverse materials at **(1)** 100 K, **(2)** 200K, and **(3)** 300K. **(a)** The Dulong-Petit estimate is found to significantly overestimate heat capacity values with MPE ranging from 26.62% to 249.8%. **(b)** The Debye model estimate is found to be significantly better than the Dulong-Petit values with MPE from 9.1% to 27.7%. However, **(c)** the VDE model estimate is found to be the most accurate, on average, with MPE values from 6.7% to 24.1%.*

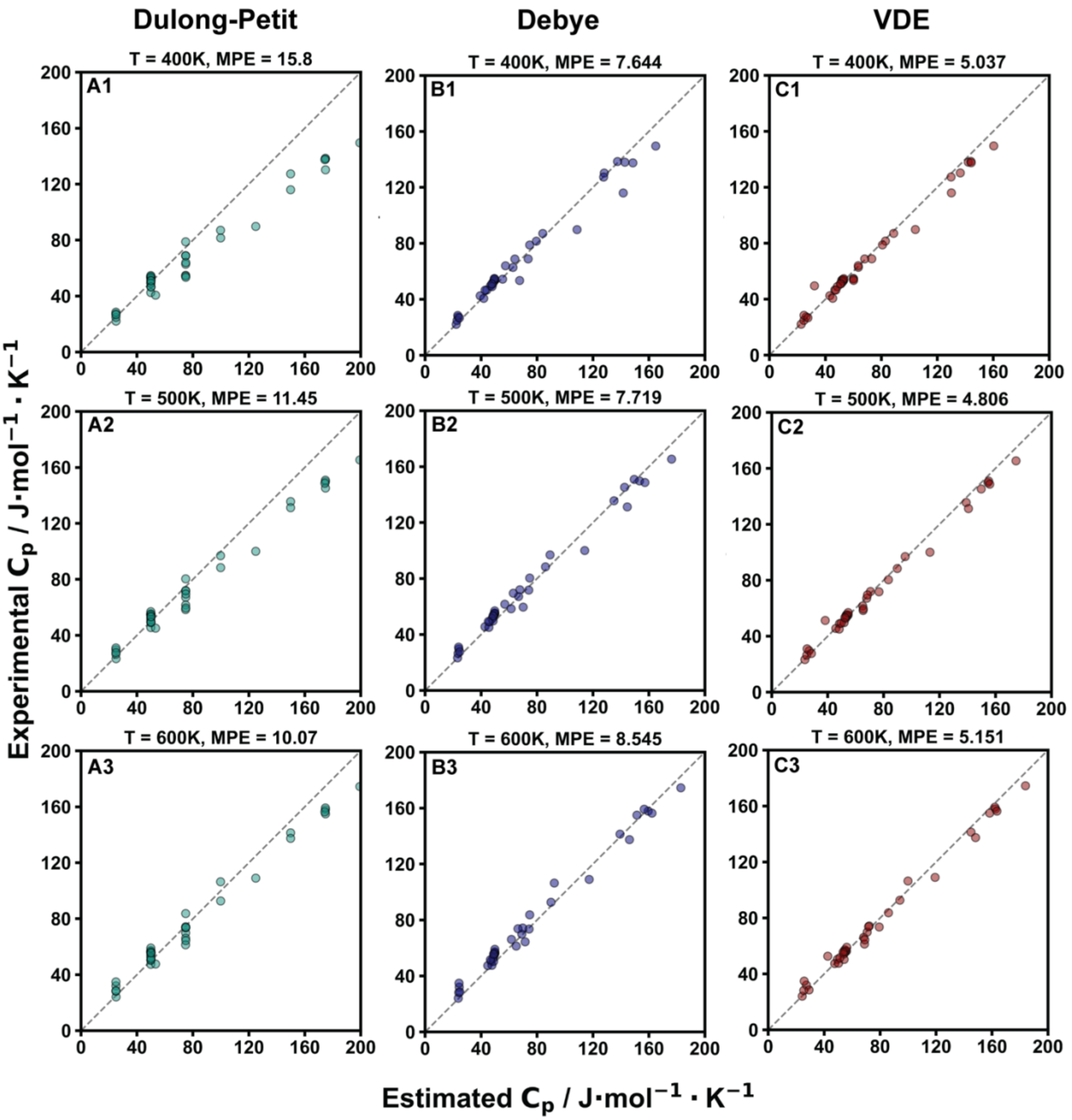

Figure S2. *Comparison of heat capacity estimates with experimental values for 38 diverse materials at **(1)** 400 K, **(2)** 500K, and **(3)** 600K. **(a)** The Dulong-Petit estimate is found to significantly overestimate heat capacity values with MPE ranging from 10.1% to 15.8%. **(b)** The Debye model estimate is found to be significantly better than the Dulong-Petit values with MPE from 7.6% to 8.5%. However, **(c)** the VDE model estimate is found to be the most accurate, on average, with MPE values between 4.8% to 5.1%.*

Mean percent error estimations as a function of temperature are shown in Figure S3, with the whole range of the error in the Dulong-Petit estimation. The Dulong-Petit model has mean percent error values approaching almost 300% at 100 K, illustrating the model's drastic inaccuracy at lower temperatures due to its constant approximation of the phonon density of states.

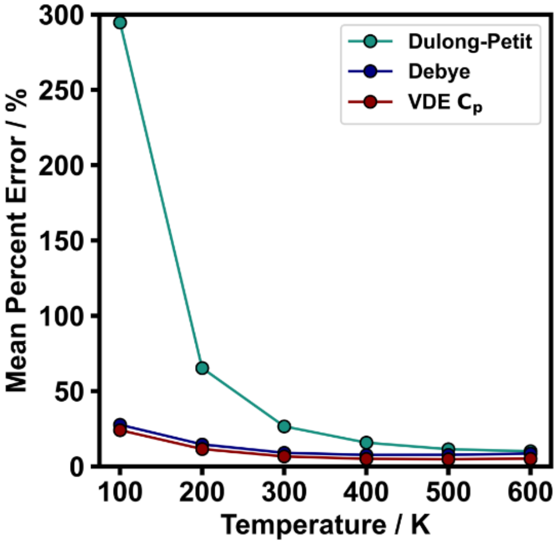

Figure S3. *Temperature dependence of mean percent error with the whole range of Dulong-Petit mean percent error shown. The same analysis of experimental constant-pressure heat capacity values and their corresponding estimated heat capacity values (as shown in Figure S1-S2) was conducted as a function of temperature for the Dulong-Petit (green), Debye (blue) and the VDE (red) models.*

Mean percent error estimations as a function of temperature are shown in Figure S4, with both the error from the VDE model's total C_V estimation (e.g. vibrational + electronic terms) and its C_p estimation (vibrational + electronic + dilation terms). The mean percent error of C_p is lower than that of C_V, which converges to the Dulong-Petit estimation at higher temperatures. This illustrates the importance of the dilation term at higher temperatures for accurate heat capacity estimations.

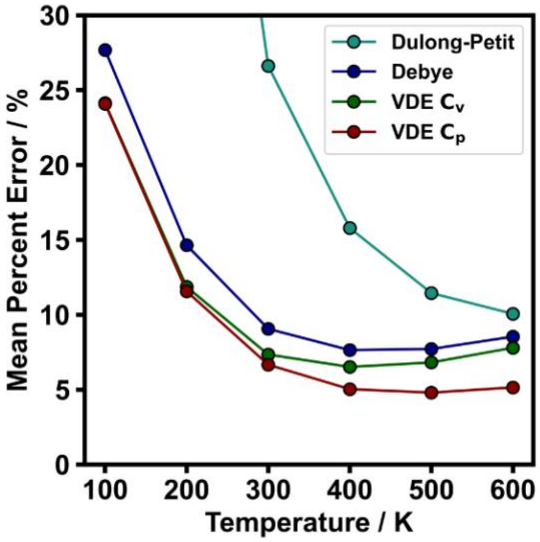

Figure S4. Temperature dependence of mean percent error with the constant-volume heat capacity also shown. The same analysis of experimental constant-pressure heat capacity values and their corresponding estimated heat capacity values (as shown in Figure S1-S2) was conducted as a function of temperature for the Dulong-Petit (green), Debye (blue) and the VDE (red) models. The constant-volume heat capacity has higher mean percent-error at high temperatures than the constant-pressure heat capacity.

Supplemental Note 3:

Structural optimizations and phonon computations were performed with DFT as implemented in VASP with plane wave basis sets.[8–11] For the electronic optimization, strict convergence energy and forces criteria EDIFF = $1E^{-7}$ and EDIFFG= -0.001 or $1E^{-5}$ per cell, respectively were used. The projector augmented-wave method with a plane wave cutoff between 520 eV and 700 eV and the Perdew-Burke-Ernzerhof (PBE) functional was used.[12–14]

Structural optimization was started from the structures provided (mp-id) in the Materials Project Database[1] (Table S8) using all allowed degrees of freedom. To ensure the same k-point mesh in the structural optimizations, a Γ-centered grid with a maximum separation of 0.12$Å^{-1}$ was employed. Harmonic vibrational frequencies and normalized phonon eigenvectors were computed using the finite displacement method as implemented in PHONOPY[4,5,15] imposing individual atomic displacements of 0.01 Å. To ensure free imaginary modes dispersion curves, supercells greater than 17 Å per lattice was used (supercell lattice convergence was tested to ensure the reliability of our Grüneisen calculations). For most of the cases, the static runs were done at gamma point. Due to challenges with convergence and/or imaginary modes in phonon dispersion curves with some materials run through the VDE model, only 25 of the 38 materials used in the VDE analysis were investigated using DFT (Table S8).

For the Grüneisen parameter calculations, two additional relaxations were also performed under fixed-volume conditions: $0.997^3 \times V_0$ (contracted volume) and $1.003^3 \times V_0$ (expanded volume), where V_0 corresponds to the ground-state volume. Subsequently, harmonic phonon calculations were conducted following the same setup of the ground-state calculations. To obtain the mode-specific Grüneisen parameter and average Grüneisen parameter, PHONOPY and classes from pymatgen were used.

Furthermore, the non-analytical correction by Gonze et al., as implemented in PHONOPY , was applied to Si (mp-7000) and LiF (mp-1138) to ensure free imaginary modes dispersion curves.[16] For the remaining structures, the effect of this correction on the Grüneisen parameter was found to be negligible.

The thermal expansion coefficient α was calculated using the average of the N mode Grüneisen parameters γ_i as

$$\gamma = \frac{1}{N}\sum\gamma_i$$

in the relation

$$\alpha = \frac{C_\mathrm{V}\gamma}{B},$$

where C_V is the sum of the vibrational and electronic terms obtained from the VDE model with the DFT-generated phonon density of states as an input, and C_p uses the above thermal expansion coefficient in the dilation term. Heat capacity estimations and their comparisons to experimental values (the same values as compared to previously with the other models in Supplemental Note 2) for the 25 materials (input parameters shown in Tables S1 and S8) using DFT are shown in Figures S5.

***Table S8**. Average Grüneisen parameter for the set of materials. For those structures, harmonic phonons were computed at gamma point.*

Formula	Material's Project ID	Average Grüneisen parameter
LiF	mp-1138	1.81
TiB_2	mp-1145	1.35
MgO	mp-1265	1.48
Si	mp-149	1.07
Al_4C_3	mp-1591	1.31
Li2O	mp-1960	1.42
FeS_2	mp-226	1.54
NaCl	mp-22862	2.31
KI	mp-22898	2.15
LiCl	mp-22905	2.86
NaBr	mp-22916	2.23
KCl	mp-23193	1.82
KBr	mp-23251	1.93
NaI	mp-23268	2.63
CaO	mp-2605	1.72
Mg_2SiO_4	mp-2895	1.34
$MgAl_2O_4$	mp-3536	1.28
KF	mp-463	2.47
Al_2SiO_5	mp-4753	1.66
TiC	mp-631	1.70
NaF	mp-682	1.93
SiO_2	mp-7000	1.06
MgB_2	mp-763	1.50
$LiAlO_2$	mp-8001	1.65
CsCl	mp-22865	2.84

*SiO_2 mp-7000 replaced mp-6930 from previous analyses

To access the full dataset of DFT-computed force constants, vibrational density of states, and Grüneisen parameters, please visit https://doi.org/10.5281/zenodo.15057994.

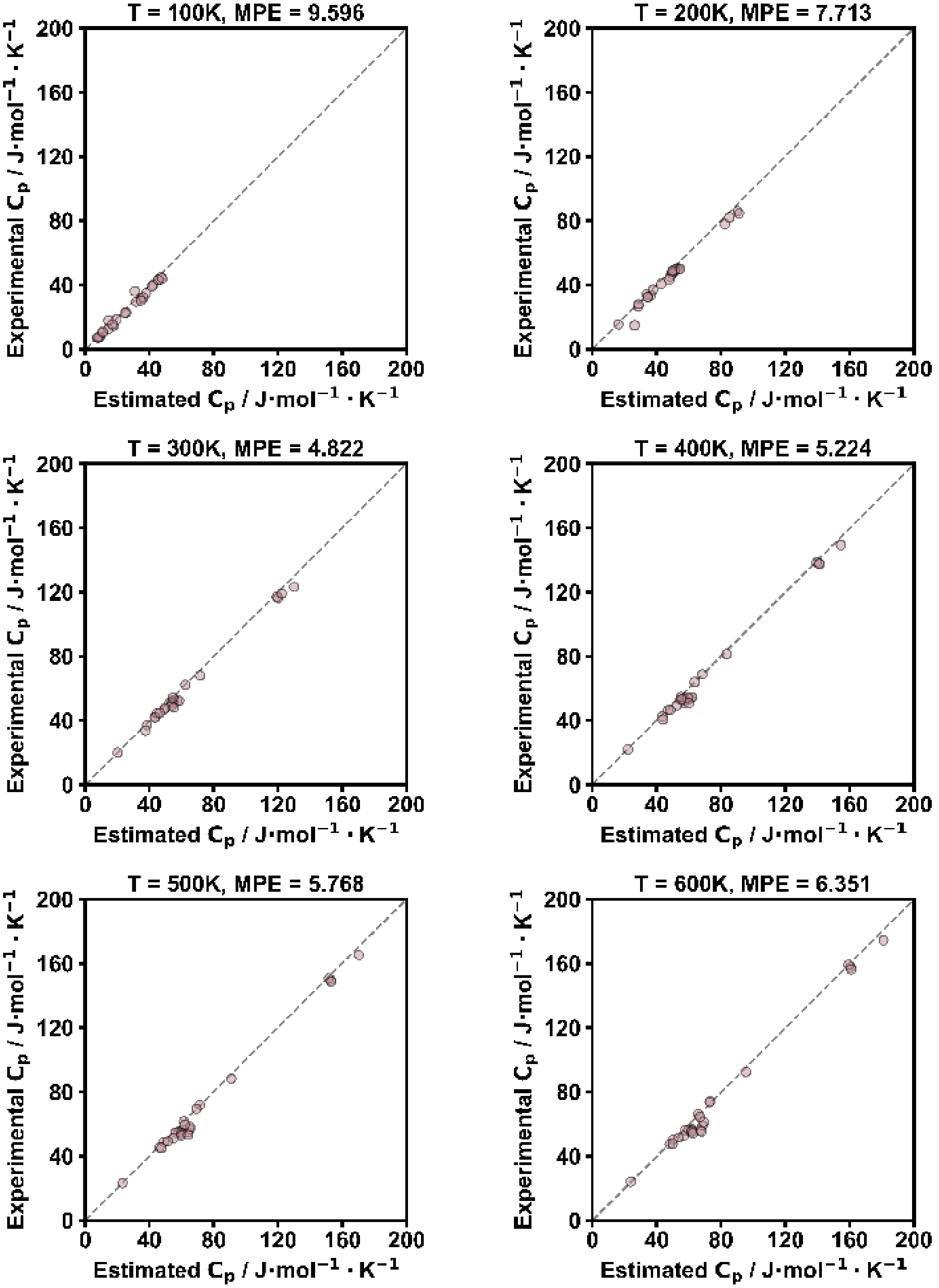

Figure S5. *Comparison of DFT heat capacity estimates with experimental values for 25 diverse materials at 100 - 600K. Mean-percent errors (MPE) are shown along with the temperature at the top of each graph.*

To compare the impact of the DFT-generated dilation term to the VDE model's dilation term, and to see the effectiveness of the DFT-generated phonon density of states in the VDE model, estimations of C_p were also generated using the DFT phonon density of states estimations, but the VDE model's dilation and electronic terms. Previously, C_v (vibrational and electronic components) were run through the VDE model, but the DFT-generated Grüneisen parameters were used for the thermal expansion coefficient in the dilation component instead of the estimation from phonon pressure theory (see Eq. 9 in the main text). A comparison of the mean percent error of the DFT heat capacity estimations can be seen in Figure S6, where no dilation is used (C_v alone), DFT dilation is used (with Grüneisen methodology described above), and with VDE dilation (from phonon pressure theory). All mean percent errors are relative to the same experimental dataset as described in the main text.

Upon inspection of Figure S6, one can see that the VDE dilation term reduces the mean percent error significantly from the estimate without (DFT-C_v) at high temperatures, but the DFT-determined dilation actually increases the mean percent error of the estimation at high temperatures. This indicates that the VDE model's dilation from phonon pressure theory brings the heat capacity estimations closer to experimental heat capacity values at higher temperatures, whereas the DFT-determined dilation has the opposite effect.

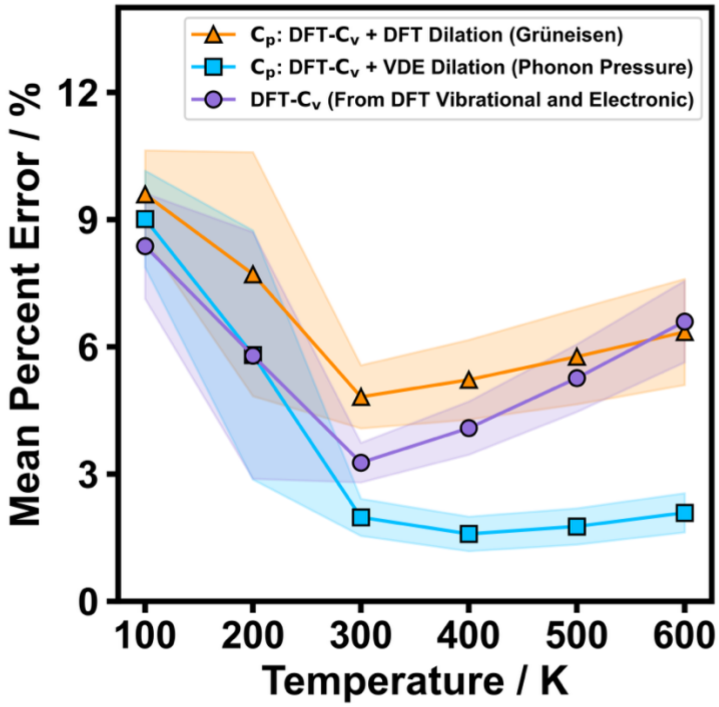

Figure S6. *Temperature dependence of mean percent error with the constant-volume heat capacity also shown. The same type of analysis of experimental constant-pressure heat capacity values and their corresponding estimated heat capacity values (as shown in Figure S3-S4) was conducted as a function of temperature for the DFT* C_v *values (purple circles), DFT* C_p *with a DFT-determined dilation term (orange triangles), and DFT* C_p *with the VDE dilation term (blue squares).*

Supplemental Note 4:

Table S9. *Inputs used for the VDE model for example materials, obtained from the Materials Project Database,*[1] *except for ZIF-8,*[17–20] *a-Si,*[21–24] *PVC,*[25–27] *and the elastic properties of* Al_2O_3*.*[28]

Formula	*Material's Project ID*	*Number of atoms*	*Density* $(g \cdot cm^{-3})$	*Unit cell volume* $(Å^3)$	*Electronic DOS at Fermi level* $(states \cdot J^{-1} \cdot m^{-3})$	*VRH bulk modulus* (GPa)	*VRH shear modulus* (GPa)	*Molecular weight* $(g \cdot mol^{-1})$	*Thermal expansion coefficient* (K^{-1})
Mg_3Sb_2	mp-2646	5	4.0	131.2	0	42	18	316.44	calculated
Si	mp-149	8	2.3	161.3	0	89	62	28.09	calculated
$LiCoO_2$	mp-22526	12	5.1	95.2	0	135	69.9	97.87	calculated
$Zn(MeIm)_2$ -SOD (ZIF-8)		102	1.0	2474.7	0	6.52	Not used	229.60	180 x 10^{-6}
Cu_2Se	mp-16366	12	7.0	195.3	0	84	6	202.06	calculated
a-Si		512	2.2	10601.6	0	75	35	28.09	calculated
PVC		24	1.5	250.5	0	4.7	Not used	62.50	31.5 x 10^{-6}
Al_2O_3	mp-1143	30	3.9	262.3	0	254	163	101.96	calculated

Supplemental Note 5:

A comparison of the VDE model predictions of C_V (e.g. vibrational + electronic terms) and its C_p (vibrational + electronic + dilation terms) for Mg_3Sb_2 is shown in Figure S7. The C_V values converge to the Dulong-Petit estimation, whereas inclusion of the dilation term in the C_p estimation brings the VDE model's prediction significantly closer to experimental heat capacity values.

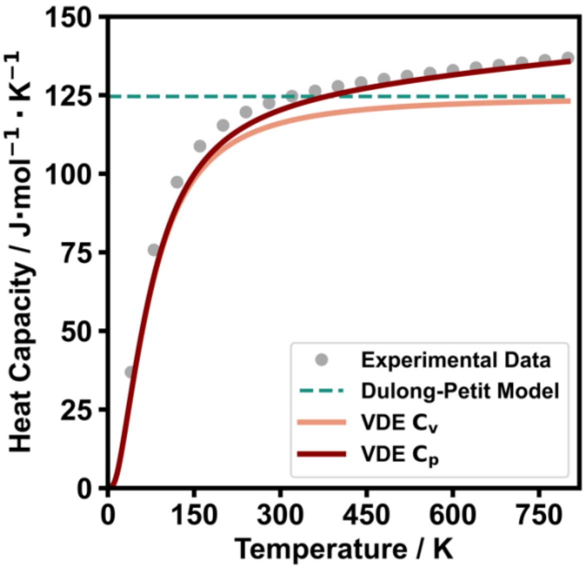

Figure S7. *Temperature dependent heat capacity of Mg_3Sb_2 with both constant volume (orange) and constant pressure (red) outputs from the VDE model. Estimations of Mg_3Sb_2 are compared to the Dulong-Petit model (teal dotted line) and to experimental values (gray points).*

Supplemental Note 6:

The phonon density of states output from the ALIGNN and MACE-MP-0-3b models are shown for ZIF-8 (Figure S8) and PVC (Figure S9). Density of states values for ZIF-8 are also compared to that reported by Hegde et al.[29] in Figure S8. Each trace was normalized such that the vibrational density of states below 120 meV integrates to 1. In ZIF-8, the full experimental phonon density of states integrates to 2.2 and the density of states for MACE-MP-0-3b integrates to 1.8 in comparison. Thus, only approximately ~45% of the vibrational modes in ZIF-8 fall within the range of energies used in the ALIGNN[30] training. Similarly in PVC, the density of states for MACE-MP-0-3b integrates to 2.2 in comparison.

In ZIF-8, an experimental value[19] for the thermal expansion coefficient of 180e-6 K^{-1} was used, as ZIF-8 is unavailable on materials project. Similarly in the case of PVC, an experimental value[24] for the thermal expansion coefficient of 31.5e-6 K^{-1} was used. As the vibrations of the hydrogen atoms are very localized, Gaussian smearing was used instead of the tetrahedron smearing method when computing the phonon densities of states with the MACE model.

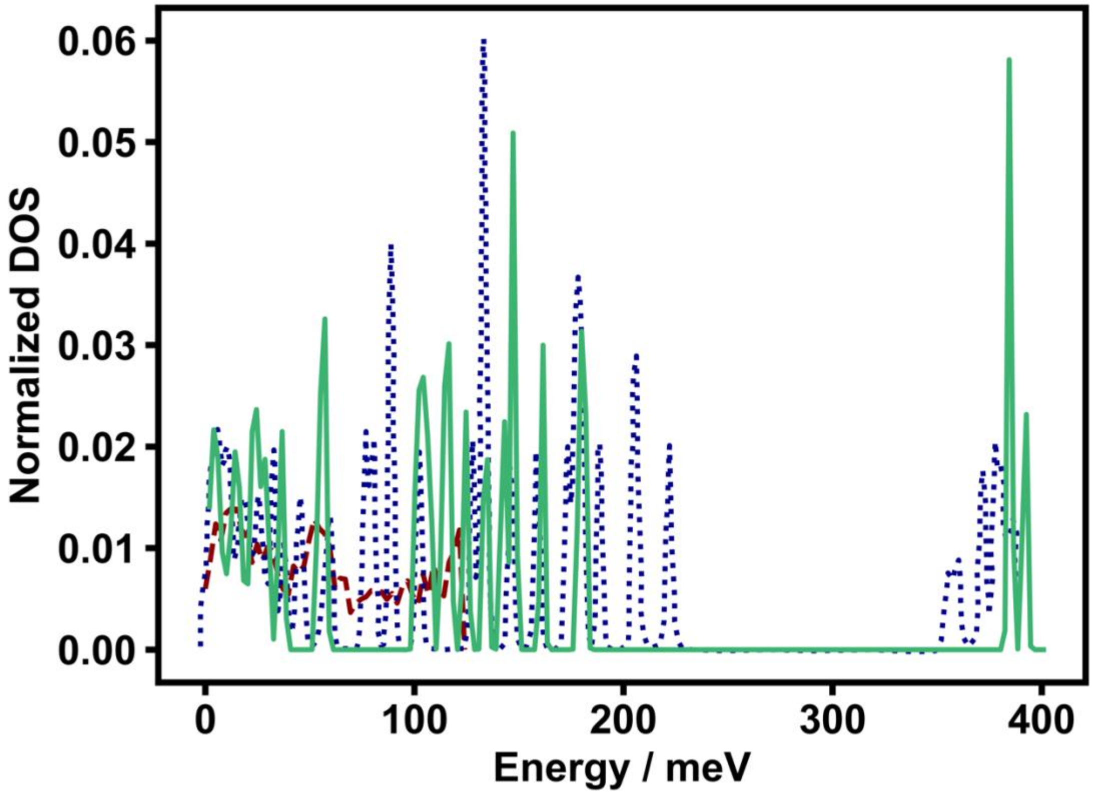

Figure S8. *Phonon density of states of ZIF-8 with the ALIGNN output (red dashed), the* MACE-MP-0-3b (green solid) *and that reported by Hegde et al.,*[29] *(blue dotted) normalized to the same scale, such that the integral of the blue dotted curve = 2.2 and red dashed curve = 1.8.*

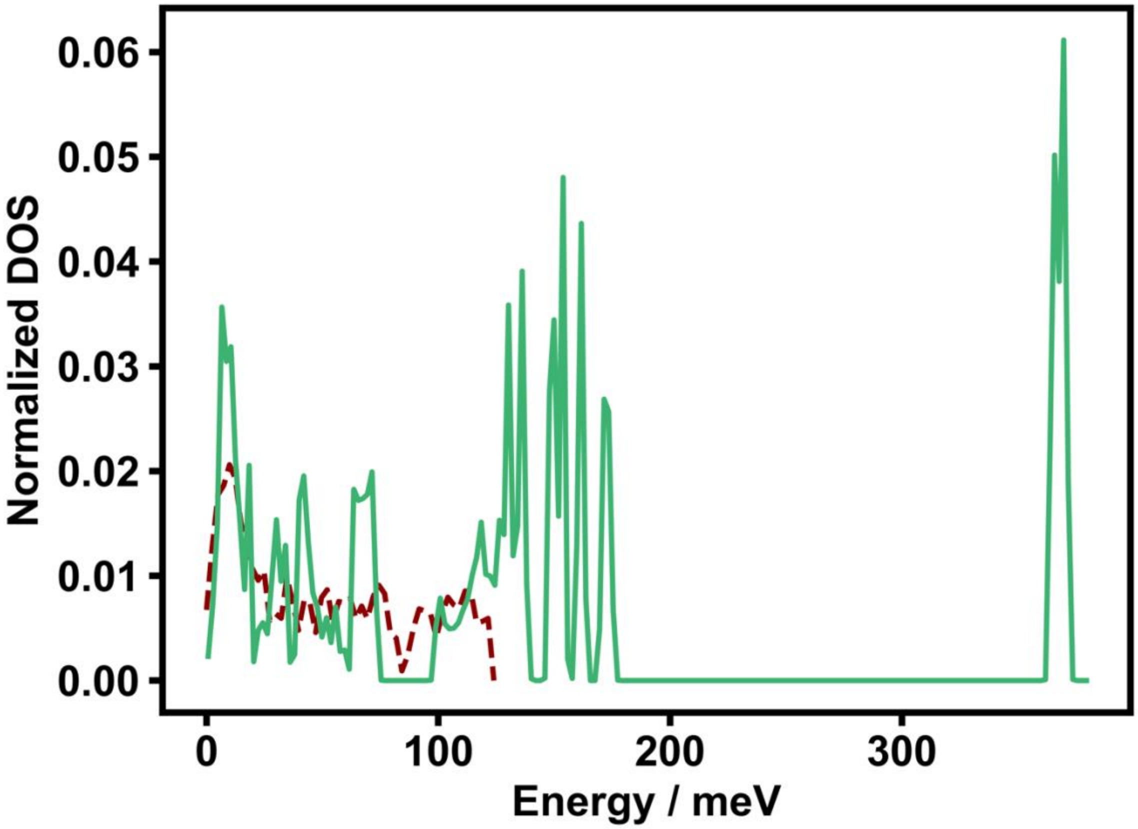

Figure S9. Phonon density of states of PVC with the ALIGNN output (red dashed) and the MACE-MP-0-3b (green solid) *normalized to the same scale such that the integral of the red dashed curve = 2.2.*

Supplemental Note 7:

The effect of different phonon density of states estimations on VDE heat capacity outputs is compared for the example materials, similar to the analysis shown in Figure 3 of the main text. The ALIGNN model, the MACE model (with the estimation procedures as mentioned in Supplemental Note 6), and the Debye model were each used as phonon density of states inputs into the VDE model, and the resulting VDE heat capacity estimations are shown in Figure S10.

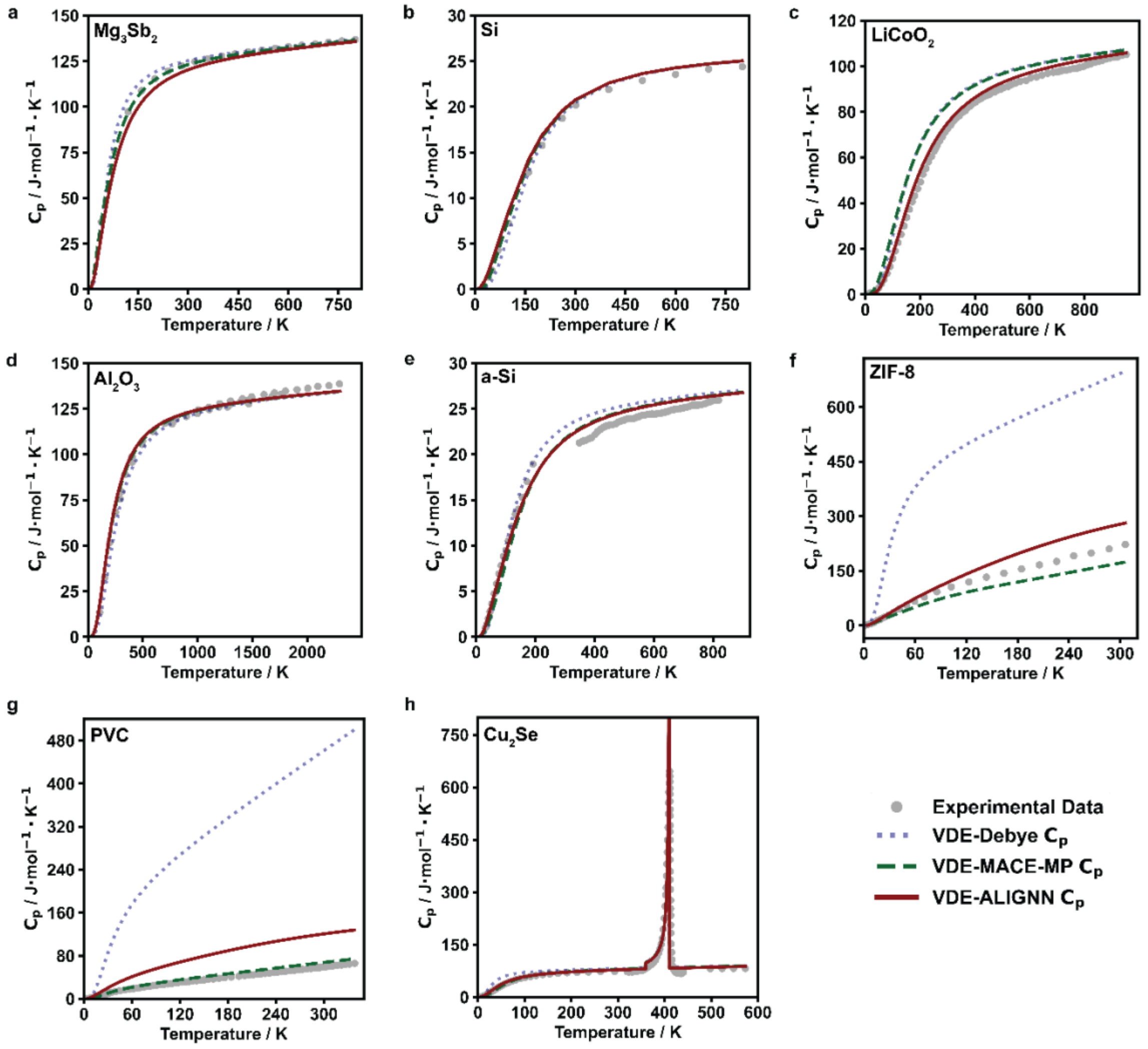

Figure S10. *Temperature dependent heat capacity estimates from the VDE model with density of states from the ALIGNN model (red line), the MACE-MP-0-3b model*[2] *with D3 dispersion (green dashed line), and Debye model (blue dotted line) for* ***(a)*** *Mg_3Sb_2,* ***(b)*** *Si,* ***(c)*** *$LiCoO_2$,* ***(d)*** *Al_2O_3,* ***(e)*** *a-Si,* ***(f)*** *ZIF-8,* ***(g)*** *PVC, and* ***(h)*** *Cu_2Se. Experimental values for Mg_3Sb_2,*[31] *Si,*[32] *$LiCoO_2$,*[33,34] *Al_2O_3,*[35–38] *a-Si,*[39,40] *ZIF-8,*[41] *PVC,*[42] *and Cu_2Se*[43–45] *are shown as gray points.*

For several of the materials (e.g. Mg3Sb2, Si, Al2O3, and Cu2Se), the three different phonon density of states estimates provide comparable heat capacity estimates, indicating that the dilation term used in the VDE model greatly contributes to the accuracy in the VDE model in those cases (when

comparing to the Debye estimates in Figures 4 and 5 of the main text, for example). However, in other cases such as that of LiCoO2, ZIF-8, a-Si, and PVC, one or the other of the machine learned models provides the most accurate heat capacity estimation when using the VDE model. This indicates that in some cases, a rough DOS estimation is acceptable when used in conjunction with a reliable dilation term; however, one cannot necessarily trust that the Debye model would be sufficient for every material that one would be interested in. In some cases, it might be useful to save computing time with the simplest estimate, but for not significantly more time and computing resources, the machine learned density of states estimates could more reliably predict heat capacity values for a wider range of materials.

Supplemental Note 8:

Thermal broadening is a technique used to smooth discontinuities resulting from piecewise functions of the phase fraction of a given material used in the phase differential for the heat capacity estimation, as in Eq. 10 in the main text. The discontinuous increase in the VDE estimation at the onset of the phase transformation is an artifact of the algorithms used to estimate ϕ_β carried through the calculations, resulting in the shift of a derivative over a linear to a non-linear region.[46] Thermal broadening algorithms have been used in previous works to alleviate this in heat capacity estimates.

Thermal broadening was applied to the VDE heat capacity estimation for Cu_2Se. The algorithm applied is a rolling average over a 3 K temperature range. The algorithm was applied three times, and the resulting trace (Figure S11) is a smoother trace than the original discontinuity.

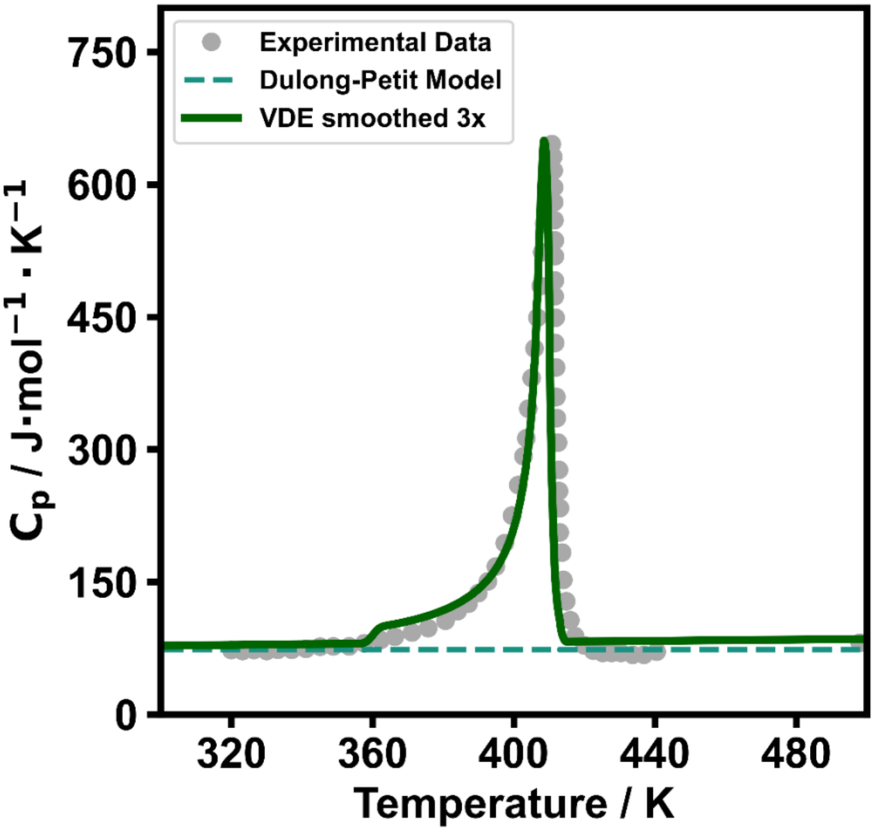

Figure S11. *Heat capacity estimations of Cu_2Se with the Dulong-Petit model (teal dotted line) and the VDE model with the added phase change contribution run through the thermal broadening algorithm three times (green). Models are compared to experimental values*[43–45] *(gray points).*

Supplemental Note 9:

The heat capacity across a multiphase system is described by the contribution of intrinsic heat capacity $C_{p\phi}$ at temperature T and pressure p of the phases present in the relation

$$C_p = \left(\frac{\partial H}{\partial T}\right)_p = C_{p\phi} + \Delta H \left(\frac{\partial \phi}{\partial T}\right)_p$$

where ΔH represents the enthalpy associated with the phase transition. For a two-phase system with phases α and β, the total heat capacity can be described by each phase's contribution and the fraction ϕ of each phase in the relation[46]

$$C_{p\phi} = \phi_\alpha(C_V^\alpha + \Gamma_\alpha T) + \phi_\beta\left(C_V^\beta + \Gamma_\beta T\right),$$

where $\phi_\alpha + \phi_\beta = 1$ and the dilation terms of each phase are represented by $\Gamma = B\alpha_V^2$. In many materials, each phase has approximately the same number of atoms/volume such that their high temperature heat capacities attain similar values. Thus, approximating C_V using a singular phase of the material is often sufficient.[46] As shown throughout this work, C_V is the largest component of C_p, and the dilation term only accounts for a small percentage. Due to similar expansion of phases, it is also sufficient to approximate the dilation terms as one phase as well. Therefore, in practice, $C_{p\phi}$ has a negligible dependence on $\phi(T)$. As a result, we have approximated $C_{p\phi}$ for Cu_2Se using only the material information for the low temperature α phase as

$$C_{p\phi} \approx (C_V^\alpha + \Gamma_\alpha T),$$

which is an approximation that holds even when the material is in the β phase, above the phase transition region from 410-660 K.

Supplemental Note 10:

As a test of how the ALIGNN phonon density of states output responds to slight volume changes in the same structure, a test of Si (mp-149) was run. Lattice parameter scaling of the structure was set to 1.0 (original scaling), 0.99, and 1.01, and the ALIGNN phonon density of states was calculated. The output shows a slight change in the phonon density of states from the original structure to the scaled structures. However, the outputs from 0.99 and 1.01 were identical, indicating that these changes in the lattice parameter scaling does impact the phonon density of states, but that the direction of scaling (e.g. slightly larger or slightly smaller) makes little difference at this scale.

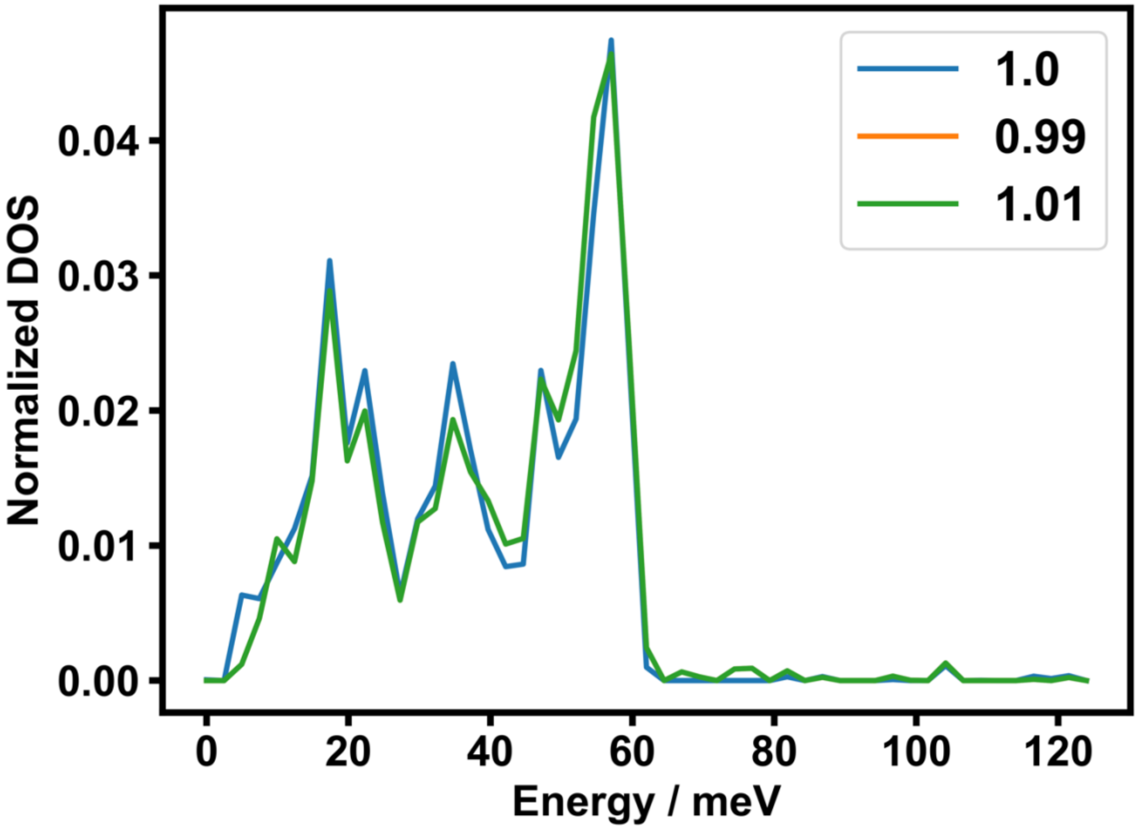

Figure S12. *Phonon density of states estimations from the ALIGNN model of Si at the original lattice parameter scaling (blue), 0.99 scaling (orange, hidden by the green trace which directly overlaps), and 1.01 (green).*

References

1. Jain, A., Ong, S.P., Hautier, G., Chen, W., Richards, W.D., Dacek, S., Cholia, S., Gunter, D., Skinner, D., Ceder, G., et al. (2013). Commentary: The Materials Project: A materials genome approach to accelerating materials innovation. APL Mater. *1*, 011002. https://doi.org/10.1063/1.4812323.

2. Batatia, I., Benner, P., Chiang, Y., Elena, A.M., Kovács, D.P., Riebesell, J., Advincula, X.R., Asta, M., Avaylon, M., Baldwin, W.J., et al. (2024). A foundation model for atomistic materials chemistry. Preprint at arXiv, https://doi.org/10.48550/arXiv.2401.00096 https://doi.org/10.48550/arXiv.2401.00096.

3. Grimme, S., Antony, J., Ehrlich, S., and Krieg, H. (2010). A consistent and accurate *ab initio* parametrization of density functional dispersion correction (DFT-D) for the 94 elements H-Pu. J. Chem. Phys. *132*, 154104. https://doi.org/10.1063/1.3382344.

4. Togo, A. (2023). First-principles Phonon Calculations with Phonopy and Phono3py. J. Phys. Soc. Jpn. *92*, 012001. https://doi.org/10.7566/JPSJ.92.012001.

5. Togo, A., Chaput, L., Tadano, T., and Tanaka, I. (2023). Implementation strategies in phonopy and phono3py. J. Phys. Condens. Matter *35*, 353001. https://doi.org/10.1088/1361-648X/acd831.

6. Ganose, A., Sahasrabuddhe, H., Asta, M., Beck, K., Biswas, T., Bonkowski, A., Bustamante, J., Chen, X., Chiang, Y., Chrzan, D., et al. (2025). Atomate2: Modular workflows for materials science. Preprint, https://doi.org/10.26434/chemrxiv-2025-tcr5h https://doi.org/10.26434/chemrxiv-2025-tcr5h.

7. Thomas C. Allison (2013). NIST-JANAF Thermochemical Tables - SRD 13. Version 1.0.2 ([object Object]). https://doi.org/10.18434/T42S31 https://doi.org/10.18434/T42S31.

8. Kresse, G., and Furthmüller, J. (1996). Efficient iterative schemes for ab initio total-energy calculations using a plane-wave basis set. Phys. Rev. B *54*, 11169–11186. https://doi.org/10.1103/PhysRevB.54.11169.

9. Kresse, G., and Furthmüller, J. (1996). Efficiency of ab-initio total energy calculations for metals and semiconductors using a plane-wave basis set. Comput. Mater. Sci. *6*, 15–50. https://doi.org/10.1016/0927-0256(96)00008-0.

10. Kresse, G., and Hafner, J. (1993). *Ab initio* molecular dynamics for liquid metals. Phys. Rev. B *47*, 558–561. https://doi.org/10.1103/PhysRevB.47.558.

11. Kresse, G., and Hafner, J. (1994). *Ab initio* molecular-dynamics simulation of the liquid-metal–amorphous-semiconductor transition in germanium. Phys. Rev. B *49*, 14251–14269. https://doi.org/10.1103/PhysRevB.49.14251.

12. Blöchl, P.E. (1994). Projector augmented-wave method. Phys. Rev. B *50*, 17953–17979. https://doi.org/10.1103/PhysRevB.50.17953.

13. Kresse, G., and Joubert, D. (1999). From ultrasoft pseudopotentials to the projector augmented-wave method. Phys. Rev. B *59*, 1758–1775. https://doi.org/10.1103/PhysRevB.59.1758.

14. Perdew, J.P., Burke, K., and Ernzerhof, M. (1996). Generalized Gradient Approximation Made Simple. Phys. Rev. Lett. *77*, 3865–3868. https://doi.org/10.1103/PhysRevLett.77.3865.

15. Togo, A., and Tanaka, I. (2015). First principles phonon calculations in materials science. Scr. Mater. *108*, 1–5. https://doi.org/10.1016/j.scriptamat.2015.07.021.

16. Gonze, X., and Lee, C. (1997). Dynamical matrices, Born effective charges, dielectric permittivity tensors, and interatomic force constants from density-functional perturbation theory. Phys. Rev. B *55*, 10355–10368. https://doi.org/10.1103/PhysRevB.55.10355.

17. Lewis, D.W., Ruiz-Salvador, A.R., Gómez, A., Rodriguez-Albelo, L.M., Coudert, F.-X., Slater, B., Cheetham, A.K., and Mellot-Draznieks, C. (2009). Zeolitic imidazole frameworks: structural and energetics trends compared with their zeolite analogues. CrystEngComm *11*, 2272–2276. https://doi.org/10.1039/B912997A.

18. Tan, J.C., Bennett, T.D., and Cheetham, A.K. (2010). Chemical structure, network topology, and porosity effects on the mechanical properties of Zeolitic Imidazolate Frameworks. Proc. Natl. Acad. Sci. *107*, 9938–9943. https://doi.org/10.1073/pnas.1003205107.

19. Chester, A.M., Castillo-Blas, C., Sajzew, R., Rodrigues, B.P., Lampronti, G.I., Sapnik, A.F., Robertson, G.P., Mazaj, M., Irving, D.J.M., Wondraczek, L., et al. (2024). Loading and thermal behaviour of ZIF-8 metal–organic framework-inorganic glass composites. Dalton Trans. *53*, 10655–10665. https://doi.org/10.1039/D4DT00894D.

20. Chapman, K.W., Halder, G.J., and Chupas, P.J. (2009). Pressure-Induced Amorphization and Porosity Modification in a Metal−Organic Framework. J. Am. Chem. Soc. *131*, 17546–17547. https://doi.org/10.1021/ja908415z.

21. Deringer, V.L., Bernstein, N., Bartók, A.P., Cliffe, M.J., Kerber, R.N., Marbella, L.E., Grey, C.P., Elliott, S.R., and Csányi, G. (2018). Realistic Atomistic Structure of Amorphous Silicon from Machine-Learning-Driven Molecular Dynamics. J. Phys. Chem. Lett. *9*, 2879–2885. https://doi.org/10.1021/acs.jpclett.8b00902.

22. Ivashchenko, V.I., Turchi, P.E.A., and Shevchenko, V.I. (2007). Simulations of the mechanical properties of crystalline, nanocrystalline, and amorphous SiC and Si. Phys. Rev. B *75*, 085209. https://doi.org/10.1103/PhysRevB.75.085209.

23. Kluge, M.D., and Ray, J.R. (1988). Elastic constants and density of states of a molecular-dynamics model of amorphous silicon. Phys. Rev. B *37*, 4132–4136. https://doi.org/10.1103/PhysRevB.37.4132.

24. Albaret, T., Tanguy, A., Boioli, F., and Rodney, D. (2016). Mapping between atomistic simulations and Eshelby inclusions in the shear deformation of an amorphous silicon model. Phys. Rev. E *93*, 053002. https://doi.org/10.1103/PhysRevE.93.053002.

25. Huan, T.D., and Ramprasad, R. (2020). Polymer Structure Prediction from First Principles. J. Phys. Chem. Lett. *11*, 5823–5829. https://doi.org/10.1021/acs.jpclett.0c01553.

26. PVC Properties Vinidex Pty Ltd. https://www.vinidex.com.au/technical-resources/material-properties/pvc-properties/.

27. Jayanna, H.S., and Subramanyam, S.V. (1992). Thermal expansion of irradiated polyvinyl chloride from 10 K to 340 K. Polym. Bull. *28*, 481–487. https://doi.org/10.1007/BF00297343.

28. Shackelford, J.F., and Doremus, R.H. eds. (2008). Ceramic and Glass Materials: Structure, Properties and Processing (Springer Science+Business Media, LLC) https://doi.org/10.1007/978-0-387-73362-3.

29. Hegde, V.I., Tan, J.-C., Waghmare, U.V., and Cheetham, A.K. (2013). Stacking Faults and Mechanical Behavior beyond the Elastic Limit of an Imidazole-Based Metal Organic Framework: ZIF-8. J. Phys. Chem. Lett. *4*, 3377–3381. https://doi.org/10.1021/jz4016734.

30. Gurunathan, R., Choudhary, K., and Tavazza, F. (2023). Rapid prediction of phonon structure and properties using the atomistic line graph neural network (ALIGNN). Phys. Rev. Mater. *7*, 023803. https://doi.org/10.1103/PhysRevMaterials.7.023803.

31. Agne, M.T., Imasato, K., Anand, S., Lee, K., Bux, S.K., Zevalkink, A., Rettie, A.J.E., Chung, D.Y., Kanatzidis, M.G., and Snyder, G.J. (2018). Heat capacity of Mg3Sb2, Mg3Bi2, and their alloys at high temperature. Mater. Today Phys. *6*, 83–88. https://doi.org/10.1016/j.mtphys.2018.10.001.

32. Porter, L.J., Yip, S., Yamaguchi, M., Kaburaki, H., and Tang, M. (1997). Empirical bond-order potential description of thermodynamic properties of crystalline silicon. J. Appl. Phys. *81*, 96–106. https://doi.org/10.1063/1.364102.

33. Gotcu-Freis, P., Cupid, D.M., Rohde, M., and Seifert, H.J. (2015). New experimental heat capacity and enthalpy of formation of lithium cobalt oxide. J. Chem. Thermodyn. *84*, 118–127. https://doi.org/10.1016/j.jct.2014.12.007.

34. Kawaji, H., Takematsu, M., Tojo, T., Atake, T., Hirano, A., and Kanno, R. (2002). LOW TEMPERATURE HEAT CAPACITY AND THERMODYNAMIC FUNCTIONS OF LiCoO2.

35. Huang, L.-F., Lu, X.-Z., Tennessen, E., and Rondinelli, J.M. (2016). An efficient ab-initio quasiharmonic approach for the thermodynamics of solids. Comput. Mater. Sci. *120*, 84–93. https://doi.org/10.1016/j.commatsci.2016.04.012.

36. Munro, R.G. (1997). Evaluated Material Properties for a Sintered alpha-Alumina. J. Am. Ceram. Soc. *80*, 1919–1928. https://doi.org/10.1111/j.1151-2916.1997.tb03074.x.

37. Schauer, A. (1965). THERMAL EXPANSION, GRUENEISEN PARAMETER, AND TEMPERATURE DEPENDENCE OF LATTICE VIBRATION FREQUENCIES OF ALUMINUM OXIDE. Can. J. Phys. *43*, 523–531. https://doi.org/10.1139/p65-049.

38. Chase, M. (1998). NIST-JANAF Thermochemical Tables, 4th Edition (American Institute of Physics, -1).

39. Tsang, K.H., Kui, H.W., and Chik, K.P. (1993). Calorimetric studies of the heat capacity and relaxation of amorphous Si prepared by electron beam evaporation. J. Appl. Phys. *74*, 4932–4935. https://doi.org/10.1063/1.354329.

40. Queen, D.R., Liu, X., Karel, J., Metcalf, T.H., and Hellman, F. (2013). Excess Specific Heat in Evaporated Amorphous Silicon. Phys. Rev. Lett. *110*, 135901. https://doi.org/10.1103/PhysRevLett.110.135901.

41. Rosen, P.F., Calvin, J.J., Dickson, M.S., Katsenis, A.D., Friščić, T., Navrotsky, A., Ross, N.L., Kolesnikov, A.I., and Woodfield, B.F. (2019). Heat capacity and thermodynamic functions of crystalline forms of the metal-organic framework zinc 2-methylimidazolate, Zn(MeIm)2. J. Chem. Thermodyn. *136*, 160–169. https://doi.org/10.1016/j.jct.2019.05.008.

42. Chang, S.-S. (1977). Heat capacity and thermodynamic properties of poly(vinyl chloride). J. Res. Natl. Bur. Stand. *82*, 9. https://doi.org/10.6028/jres.082.002.

43. Liu, H., Shi, X., Xu, F., Zhang, L., Zhang, W., Chen, L., Li, Q., Uher, C., Day, T., and Snyder, G.J. (2012). Copper ion liquid-like thermoelectrics. Nat. Mater. *11*, 422–425. https://doi.org/10.1038/nmat3273.

44. Liu, H., Yang, J., Shi, X., Danilkin, S.A., Yu, D., Wang, C., Zhang, W., and Chen, L. (2016). Reduction of thermal conductivity by low energy multi-Einstein optic modes. J. Materiomics *2*, 187–195. https://doi.org/10.1016/j.jmat.2016.05.006.

45. Brown, D.R., Heijl, R., Borup, K.A., Iversen, B.B., Palmqvist, A., and Snyder, G.J. (2016). Relating phase transition heat capacity to thermal conductivity and effusivity in Cu_2Se. Phys. Status Solidi RRL – Rapid Res. Lett. *10*, 618–621. https://doi.org/10.1002/pssr.201600160.

46. Agne, M.T., Voorhees, P.W., and Snyder, G.J. (2019). Phase Transformation Contributions to Heat Capacity and Impact on Thermal Diffusivity, Thermal Conductivity, and Thermoelectric Performance. Adv. Mater. *31*, 1902980. https://doi.org/10.1002/adma.201902980.
