# Supplementary material for: Heat capacity estimation of complex materials for energy technologies
Source: Joule. 2025 Aug 20;9(8):None. doi: 10.1016/j.joule.2025.102054 (PMC12369388; doi:10.1016/j.joule.2025.102054)
Supplement: Document S2. Article plus supplemental information [file mmc2.pdf]

Joule

CellPress

Perspective

Heat capacity estimation of complex materials for energy technologies

Elana J. Cope,[1] Joana Bustamante,[2] Zöe M. Johnson,[1] Alicia Lancaster,[1] Ramya Gurunathan,[3] Janine George,[2,4] and Matthias T. Agne[1,5,*]

[1]Department of Chemistry and Biochemistry, University of Oregon, Eugene, OR, USA
[2]Federal Institute for Materials Research and Testing (BAM), 12205 Berlin, Germany
[3]Independent Contributor, Durham, NC, USA
[4]Institute of Condensed Matter Theory and Solid-State Optics, Friedrich Schiller University, 07743 Jena, Germany
[5]Materials Science Institute, University of Oregon, Eugene, OR, USA
*Correspondence: agne@uoregon.edu
https://doi.org/10.1016/j.joule.2025.102054

CONTEXT & SCALE The control of heat in energy materials is one of the greatest current engineering challenges. Accurate estimations of heat capacity are key in creating and using materials safely and efficiently. Current models for heat capacity are often limited due to crude estimations of the phonon density of states, which is a key component of the thermodynamic definition of heat capacity. Utilization of a more detailed phonon density of states, which can easily be obtained from machine-learned algorithms, combined with dilation and electronic contributions, yields heat capacity estimations that are 29% better than the widely utilized Debye model and are comparable to state-of-the-art quantum mechanical calculations. The framework and necessary tools for heat capacity estimations demonstrated herein can be built into more detailed models and analyses, such as high-throughput characterization, transport models, or other thermodynamic assessments. Consequently, the so-called vibrational + dilation + electronic (VDE) model of heat capacity developed in this work better enables the informed design of next-generation devices.

SUMMARY

Heat capacity, which directly relates to free energy changes and thermal transport, is fundamental to modern engineering design. Even though current computational technology provides a detailed picture of atomic vibrations, the Debye and Dulong-Petit models are still widely utilized despite being prone to lower accuracy. Modern considerations of vibrational states, anharmonicity, electronic carriers, and phase transformations could improve estimates. Herein, the physics-based vibrational + dilation + electronic (VDE) model incorporates a user-provided phonon density of states, a phonon pressure-based dilation term, and an electronic component. Phonon density of states from analytical, machine-learned, and first-principles methods are compared, thus highlighting the advantages of machine-learned technology. Heat capacity estimates for 38 diverse materials are often within 5% of experimental values between 200 and 600 K. Detailed temperature-dependent investigations are carried out for several materials, including $LiCoO_2$, ZIF-8, Mg_3Sb_2, polyvinyl chloride (PVC), and amorphous silicon. Cu_2Se is modeled through its phase transition, which further demonstrates the model's capabilities to enable engineering design and sophisticated analysis.

INTRODUCTION

Thermal management is the crux of many modern engineering design problems. Effective control over the spatial temperature profile in electronic and chemical systems is often a limiting factor for device operation and performance.[1,2] Consequences of failure in thermal management range from inefficient energy usage at best to dangerous catastrophes at worst. For example, inclusion of materials with mismatched thermal expansion coefficients could cause the thermal barrier coatings on turbine blades to crack[3] or semiconductor interfaces of a thermoelectric module to fail.[4] Catastrophic failure of lithium-ion batteries occurs when temperatures rise high enough to promote self-propagating reactions,[5–8] a process named as thermal runaway. Microprocessors,[9] latent heat storage systems,[10] and quantum technologies[11] benefit from fast heat transport, whereas refractory processes[12] and industrial chemical reactions often benefit from heat retention. Considerations of the temperature dependence of chemical

stability, e.g., for organic and perovskite materials used in optoelectronic and photovoltaic devices,[13,14] and the optimization of operating temperatures, e.g., for metal-organic frameworks (MOFs) used in carbon capture,[15] are key components of technological success. Thermoelectric devices, which utilize the solid-state Seebeck effect to convert heat directly into electricity, represent a unique challenge in that a large temperature gradient must be sustained across the device, necessitating the development of ultra-low thermal conductivity materials.[16] Thus, an accurate description of the thermal properties of materials is integral to next-generation device design. In particular, heat capacity and thermal conductivity must be well understood in order to accurately model the thermodynamic state and temperature distributions during device operation.

Heat capacity describes how much heat (thermal energy) must be added or removed from the material to change the temperature by a certain amount. It is a fundamental thermodynamic quantity whose magnitude is defined by the various microscopic mechanisms available for heat storage. In solids, this means that heat capacity is intimately related to the frequency distribution of atomic vibrations that store thermal energy in phonon occupations.[17] Additionally, electronic carriers or phenomena such as phase transformations[18] can also contribute to the total heat capacity. It follows that the estimation and analysis of heat capacity in technological materials requires an understanding of the microscopic mechanisms at play in the temperature range of interest.

The thermodynamic relationship between thermal conductivity κ, thermal diffusivity D, density ρ, and heat capacity (at constant pressure) c_p,[19]

$$\kappa = \rho c_p D, \qquad \text{(Equation 1)}$$

means that estimations of κ from thermal diffusivity measurements are heavily dependent on accurate values of c_p. In this case, c_p is an intrinsic quantity with units of $J \cdot g^{-1} \cdot K^{-1}$ and is multiplied by ρ to obtain the heat capacity per volume with units of $J \cdot m^{-3} \cdot K^{-1}$ in the relation $C_p = \rho c_p$. For thermoelectric materials, inaccurate estimates of heat capacity in Equation 1 will directly impact the estimation of the thermoelectric conversion efficiency.[19] Given the widespread use of Equation 1 for estimating thermal conductivity near room temperature and above, this work primarily focuses on accurately characterizing heat capacity at these temperatures (>100 K), but low-temperature (>2 K) investigations are also demonstrated.

The relevance of heat capacity to other thermal properties such as thermal expansion[20] $(\alpha \propto C_p)$ and thermal effusivity[21] $(\epsilon \propto \sqrt{C_p})$ makes it foundational to understanding thermophysical properties broadly, in addition to being essential for thermodynamic assessments.[22,23] As scientists and engineers become increasingly interested in developing diverse materials such as those for energy conversion/storage, semiconductor and quantum technologies, etc., the ability to accurately estimate the magnitude and temperature dependence of heat capacity is crucial for material analysis and development. Because experimental heat capacity measurements can be inconsistent, particularly at high temperatures where independent measurements can deviate by $\sim$20%,[24] reliable physics-based estimates of heat capacity offer a straightforward solution. Historically, the Dulong-Petit and Debye models of heat capacity have been widely used because they are easily determined from accessible material properties, but they have limitations. For example, the Debye model aims to estimate the maximum vibrational frequency of a solid but is actually a better estimate of the average vibrational frequency for many materials.[25,26] Various attempts to improve beyond the Debye model have been undertaken in thermal conductivity models.[26,27] Although many analytical models of the vibrational spectrum do capture the first-order physics of heat capacity, it is now possible to build a more detailed model with similarly accessible inputs. Herein, a method of estimating heat capacity is presented that utilizes a crystallographic input file (CIF) structure file and material properties such as the bulk and shear moduli (which often can be easily obtained from sources like Materials Project). Importantly, this study provides a starting point for constructing more detailed models of heat capacity, including through phase transformation regions.

THERMODYNAMIC DEFINITION OF HEAT CAPACITY

As most practical experimental characterization of solids is undertaken under constant pressure conditions, the focus of this work will be to describe the heat capacity at constant pressure, written as an intrinsic quantity (normalized by volume) as

$$C_p = \frac{1}{V}\left(\frac{\partial H}{\partial T}\right)_p, \qquad \text{(Equation 2)}$$

where H is the total enthalpy contained by the material with volume V. Although enthalpy is often dominated by thermal processes, it can also have contributions from phase transformations. In the absence of phase transformations, the constant pressure heat capacity C_p (in units of $J \cdot m^{-3} \cdot K^{-1}$) can be defined by the thermodynamic relation

$$C_p = C_V + B\alpha^2 T = C_V(1 + \gamma\alpha T) \qquad \text{(Equation 3)}$$

that relates to heat capacity at constant volume C_V, the isothermal bulk modulus B, and volumetric thermal expansion coefficient α or equivalently with the thermodynamic Grüneisen parameter γ. Implicitly, all the terms in Equation 3 are temperature dependent. Inspection of Equation 3 already suggests that C_p may be described to varying degrees of approximation, with C_V being a good first approximation that can be improved upon by considering the so-called dilation component that has an explicit linear temperature dependence $(B\alpha^2 T)$.

In anticipation of the importance of C_V in estimating the magnitude of C_p, it is important to note that C_V is defined from the total internal energy U (at constant volume) as

$$C_V = \frac{1}{V}\left(\frac{\partial U}{\partial T}\right)_V, \qquad \text{(Equation 4)}$$

which is particularly beneficial as U is often accessible to estimate from theoretical considerations. The isothermal bulk modulus,

$B = -(\partial p/\partial \ln V)_T$, is an elastic modulus of the material, whereas the thermal expansion coefficient, $\alpha = (\partial \ln V/\partial T)_p$, and thermodynamic Grüneisen parameter, $\gamma = \alpha B/C_V$, are anharmonic properties of the solid that describe the thermal pressure that drives the material's change in volume with temperature. From the point of view that heat capacity is related to the degrees of freedom available for the material to store thermal energy, the dilation term represents the additional heat that can be stored by doing work against the surroundings to make more space for itself.

MICROSCOPIC CONSIDERATIONS OF HEAT CAPACITY

Dulong and Petit made the empirical observation (ca. 1819) that solids have a heat capacity of $\sim 3k_B$ per atom near room temperature, where k_B is the Boltzmann constant and is related to the gas constant R through Avogadro's number N_A in the relation $R = N_A k_B$. From the equipartition theorem, which postulates that energy is equally divided between available degrees of freedom, this suggested that atomic motion is likely a dominant contributor to heat capacity. In the kinetic theory of ideal monatomic gases, kinetic energy distributed across three spatial dimensions gives heat capacity a value of $3k_B/2$ per atom (at constant volume). Atoms vibrating in solids have kinetic energy, but they also have strong interatomic interactions (bonds) that contribute an additional $3k_B/2$ per atom from potential energy, bringing the total to $3k_B$ per atom for a classical harmonic oscillator. Note that deviations from $3k_B$ per atom are possible when considering anharmonic oscillators at constant volume, which may amount to an increase in the contribution from C_V (Equation 4) by a few percent near room temperature and $\sim$10% at 1,500 K.[28] Nevertheless, the Dulong-Petit model of heat capacity is applicable when atomic vibrations can be considered as classical harmonic oscillators at high temperatures.

Because the Dulong-Petit heat capacity is representative of the heat capacity in the high-temperature limit, the thermoelectrics community has a long history of using it to approximate the heat capacity at temperatures near and above 300 K.[29] The maximum vibrational frequencies of the atoms in many thermoelectric materials are already excited by these temperatures, such that around room temperature, the relation $C_p \approx C_V \approx 3k_B/$atom oftentimes holds. However, in many engineering solids relevant for semiconductor/microelectronic, photovoltaic, battery, or mechanical applications, the highest vibrational frequencies are not significantly excited by 300 K, thus making that model insufficient. For example, at 300 K, the heat capacity of silicon is only $\sim$80% of the Dulong-Petit value.[30] Additionally, heat capacity can have a strong temperature dependence around room temperature. Meyer-Kelly-type equations for temperature-dependent heat capacity can often be found in reference books, but these empirical descriptions are fit to experimental data and cannot be determined a priori. Using a physics-based model for heat capacity that captures temperature dependence is distinctly advantageous for computational materials design or in the absence of experimental capabilities.

At lower temperatures, heat capacity drops sharply from the Dulong-Petit value. Einstein recognized that this results from the fact that solids are quantum objects with changing occupations of the quantized states of different vibrational modes.[31] There is not enough thermal energy available at low temperature to populate higher vibrational states, so those vibrations are "frozen out." Using quantum statistics, he was able to better predict the temperature dependence of heat capacity while still reaching the Dulong-Petit value at high temperature. Later, Debye proposed that atomic vibrations have a distribution of frequencies, unlike the single-frequency approximation that Einstein made. The concept of a continuum nature of the phonon density of states led to a better prediction of the temperature dependence of heat capacity at low temperatures, famously known as the Debye T-cubed law.[31]

While the Einstein and Debye models brought temperature-dependent estimates of heat capacity closer to observed values, the crude estimates of the phonon density of states neglect the nuanced nature of atomic vibrations specific to each material, causing inaccuracies in the estimation of heat capacity. Additionally, it is commonly observed that C_p tends to exceed the Dulong-Petit/Debye predictions at high temperatures, suggesting that the Dulong-Petit and Debye models may be a good first approximation for C_p, but a more complete description of a material's phonon density of states and dilation propensity is expected to be more accurate.

Temperature-dependent models of C_p can be constructed starting from Equation 3. In addition to the explicit temperature dependence of the dilation term, it is also necessary to consider each of the microscopic mechanisms that may contribute appreciably to C_V and their temperature dependence. The total heat capacity at constant volume is then the sum of constant volume heat capacities from different sources, e.g., vibrational, electronic, etc., as

$$C_V = C_V^{\mathrm{vib}} + C_V^{\mathrm{elec}} + \ldots \qquad \text{(Equation 5)}$$

Consideration of the vibrational component is necessary for all solids, whereas the electronic contribution may only be needed in metallic-like materials (e.g., having electronic carrier concentrations greater than $\sim 10^{22}$ carriers$\cdot$cm^{-3}). Other contributions to heat capacity, e.g., magnetism,[32] can be considered as necessary. From a quantum mechanical treatment of atomic vibrations as harmonic oscillators with angular frequency ω following Bose-Einstein statistics, the vibrational contribution to heat capacity at constant volume can be described as[17]

$$C_V^{\mathrm{vib}} = 3nk_B \int_0^{\infty} \left(\frac{g(\omega)}{3n}\right)\left(\frac{\hbar\omega}{k_B T}\right)^2 \left(e^{\frac{\hbar\omega}{k_B T}}\right)\left(e^{\frac{\hbar\omega}{k_B T}} - 1\right)^{-2} d\omega \qquad \text{(Equation 6)}$$

for a collection of oscillators with a spectral distribution defined by the phonon density of states $g(\omega)$. Note that $g(\omega)/3n$ is called the normalized density of states ($\int_0^{\infty} (g(\omega)/3n) d\omega = 1$), where n is the number density of atoms (atoms$\cdot$m^{-3}). The electronic term for constant volume heat capacity is derived from the Sommerfeld expansion as

$$C_V^{\mathrm{elec}} = \frac{\pi^2}{3} k_B^2 D(E_F) T, \qquad \text{(Equation 7)}$$

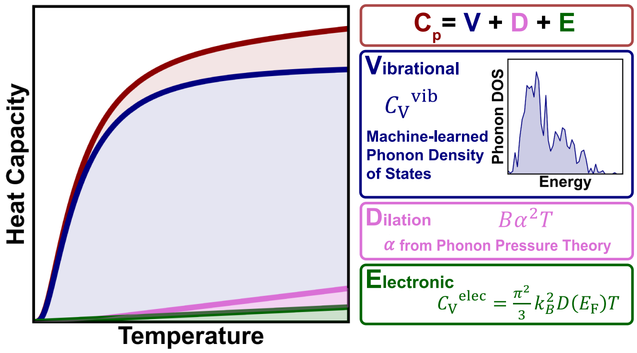

Figure 1. Construction of the VDE model

The components of the VDE model are shown as vibrational (blue) using a machine-learned density of states algorithm, dilation (pink) using phonon pressure theory, and electronic (green) using the electronic density of states at the Fermi level, which all add together to get the VDE C_p estimate (red).

where $D(E_F)$ is the value of the electronic density of states at the Fermi level (states $\cdot J^{-1} \cdot m^{-3}$). Notably, the contribution of electronic carriers to heat capacity in this model has a linear temperature dependence.

Thus, for a vast majority of materials, the temperature dependent C_V (Equation 5) can be estimated when estimations of the phonon density of states and electronic density of states are available. Then, values of C_p can be obtained from Equation 3 with the inclusion of experimental or computational values of bulk modulus and thermal expansion coefficient.

In this work, heat capacity values are often reported in units of $J \cdot mol^{-1} \cdot K^{-1}$. These units are obtained by multiplying volumetric heat capacity values (i.e., Equation 3), having units of $J \cdot m^{-3} \cdot K^{-1}$, by the molar volume of the material V_m ($m^{-3} \cdot mol^{-1}$), where V_m is derived from the material's formula weight M_w ($kg \cdot mol^{-1}$) divided by the material's density ρ ($kg \cdot m^{-3}$) as $V_m = M_w / \rho$.[24]

METHODS

Here, we introduce a physics-based estimation of C_p based on Equation 3 that we call the VDE model (short for vibrational + dilation + electronic). For a detailed description of the model and relevant Python code, see Note S1. Although the VDE model requires mostly the same inputs as the Debye model, it is distinct in its incorporation of a machine-learned phonon density of states, a novel estimate of the dilation term, and the electronic term. Explicitly, the VDE model (Figure 1) accounts for the vibrational (Equation 6) and electronic (Equation 7) contributions to C_V, along with the dilation term, as

$$C_p = C_V^{vib} + C_V^{elec} + B\alpha^2 T, \quad \text{(Equation 8)}$$

which requires an estimation of the phonon density of states $g(\omega)$, the electronic density of states at the Fermi level $D(E_F)$, the bulk modulus B, and the thermal expansion coefficient α. The temperature dependence of $g(\omega)$, $D(E_F)$, B, and α is not included in the model, as the explicit temperature dependence of C_p from Bose-Einstein statistics and the linear T terms were found to be sufficient, as will be shown. While many previous heat capacity models have relied on the Debye and Einstein models of the phonon density of states, the field of machine learning is under rapid development, and it is now possible to use a machine-learned estimate of the phonon density of states.[33,34]

In this study, the pre-trained model available in the ALIGNN Python package (model = jv_pdos_alignn) is used.[35] ALIGNN is a graph neural network that encodes crystal structures as non-Euclidean graphs, where nodes, edges, and edge pairs capture atomic properties, bond lengths, and bond angles, respectively. The output of the ALIGNN Phonons model is a discretized phonon density of states with phonon density of states values specified in the frequency range of -300 to 1,000 cm^{-1} (~ -37 to 120 meV) with a bin size of 20 cm^{-1} ($\sim$2.5 meV). The ALIGNN phonon density of states model was trained on 14,000 phonon structures determined from density functional theory (DFT) calculations maintained by the National Institute of Standards and Technology in the NIST-JARVIS database.[36] These phonon calculations were performed using the OptB88vdw functional using an automated k-point and energy cutoff convergence methodology.[37] The dataset was randomly divided into an 80%-10%-10% training-validation (during training)-test (fully blind) split. Additional training and validation details can be found in the original paper presenting the model.[35]

The ALIGNN vibrational density of states estimation does not explicitly account for temperature dependence or anharmonic contributions at constant volume, but temperature-dependent structure files could be used as inputs. Other phonon density of states estimations could also be utilized, e.g., different machine-learned models,[38–40] or those from DFT, force fields,[33,41] or experiments. As a comparison, we show VDE results using phonon density of states estimates from a different machine-learned model that uses force fields, known as the MACE-MP-0-3b model[33] with D3 dispersion[42] (Note S1), hereafter referred to as MACE. Compared with analytic models, the ALIGNN and MACE phonon density of states offer a more accurate distribution of phonon modes, including van Hove singularities and acoustic-optical band gaps. For the purposes of estimating C_V, only the phonon density of states at positive frequencies ($\geq$ 0 meV) is utilized, and negative values are set to zero before the density of states is normalized (see Note S1).

A powerful feature of the ALIGNN predictive model is that it only requires a CIF or other structure file (POSCAR or PDB) to generate the phonon density of states estimation. CIF files contain information about the atomic structure of materials, which can be determined experimentally (e.g., X-ray diffraction) or found from DFT structure optimization. In this study, we utilize the CIF files available on the Materials Project database[43] unless otherwise stated. The ALIGNN phonon density of states output, plus Materials Project values for bulk modulus (i.e., B_{VRH}), shear modulus (i.e., G_{VRH}), electronic density of states, volume, density, and number of atoms in the structure are used in our high-throughput assessment of heat capacity. The thermal expansion coefficient is estimated from phonon pressure theory[44] as

$$\alpha \approx \frac{3}{2} \frac{C_V}{\rho v^2}, \quad \text{(Equation 9)}$$

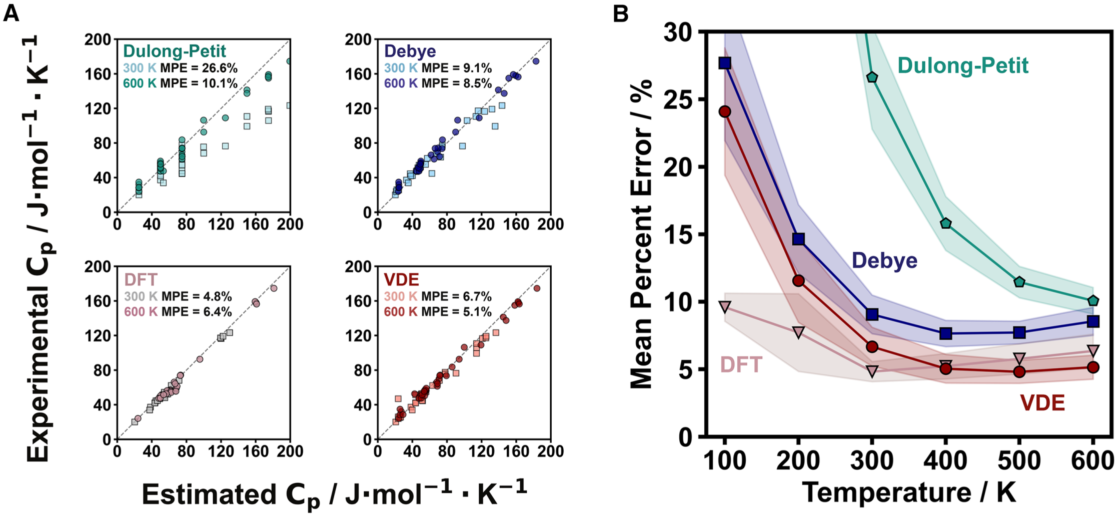

Figure 2. Comparison of heat capacity models with experimental values for diverse materials

(A) The Dulong-Petit estimate is found to significantly overestimate heat capacity values with a mean percent error (MPE) of 26.6% at 300 K (light teal squares) and 10.1% at 600 K (dark teal circles). The Debye model estimate is found to be significantly better than the Dulong-Petit values with MPE values of 9.1% at 300 K (light blue squares) and 8.5% at 600 K (dark blue circles). DFT values at 300 K (light gray squares) have an MPE value of 4.8%, and at 600 K (pink circles), the MPE is 6.4%. The VDE model estimates are found to be comparable to DFT-level accuracy at higher temperatures with MPE values of 6.7% at 300 K (light red squares) and 5.1% at 600 K (dark red circles).

(B) The MPE was assessed as a function of temperature for the Dulong-Petit (teal pentagons), Debye (blue squares), DFT (pink triangles), and VDE (red circles) models (Notes S2 and S3). The VDE model is seen to be ~ 29% more accurate than the Debye model on average and comparable to DFT estimates of heat capacity over a wide temperature range. Shaded regions represent the standard error of the mean at each temperature.

where $\rho v^2 = (10G + 3B)/9$ is an average elastic modulus defined by the root mean square speed of sound. Thermal expansion is usually attributed to anharmonicity, which is related to various factors in the local bonding environment like coordination number and long-range interactions.[45] However, thermal expansion may also be described within the context of elastic continuous medium theory, which characterizes each atom as an isolated oscillator with its own local mechanical equilibrium.[44] In this latter case, it is possible to predict the thermal expansion coefficient from harmonic descriptors (Equation 9), and this estimation of α is expected to be within a factor of $\sim$2 of DFT-determined values. Given the aforementioned inputs, the VDE model yields a heat capacity prediction across a desired temperature range.

HIGH-THROUGHPUT VDE MODEL OF HEAT CAPACITY

Upon running the VDE model on 38 different materials, we compare their estimated C_p with experimental C_p values reported in the NIST-JANAF Tables[46] (Note S2). Heat capacity estimations using the Dulong-Petit and Debye models were undertaken for comparison, as well as DFT estimations of C_p for 25 of those materials (Note S3). Exemplary correlations of the estimated and experimental C_p values are shown for 300 and 600 K (Figure 2A). While the Dulong-Petit and Debye models tend to overestimate experimental C_p values, the VDE model provides a better estimate, as indicated by a lower mean percent error (MPE). In fact, by tracking the MPE as a function of temperature (Figure 2B), we find that the VDE model performs with DFT-level accuracy in estimating experimental heat capacity values across a wide temperature range (within $\sim$7% from 300 to 600 K) and is at least 29% more accurate than the Debye model on average. Even at the highest temperature evaluated in this comparison (600 K), the Dulong-Petit estimate still has the largest deviation from experimental values. Being temperature independent, the Dulong-Petit model performs progressively worse at lower temperatures (Note S2). Although the Debye model captures the general temperature dependence of heat capacity, it is not as predictive as the VDE model largely because it cannot capture the same degree of complexity of the phonon density of states. At higher temperatures, the dilation term becomes more important and contributes an additional benefit to the VDE model over the Debye model, as seen in the comparison between C_p and C_V (Note S2).

By inspection of Figure 2, it is apparent that the VDE model estimations of heat capacity are predictive of experimental values for diverse materials over a wide temperature range. Thus, we anticipate that the VDE model can be reliably used as an estimate for the heat capacity when experimental measurements are not available or as a starting point for more detailed investigation of thermophysical properties. Given the complexity of many materials, as well as limited access to low-temperature heat capacity measurements and the uncertainties (e.g., $\sim$20%) associated with high-temperature heat capacity measurements, the VDE model meets the essential needs for accurate predictions. While capturing typical contributions to heat capacity (i.e., vibrational, dilation, and electronic) over a large temperature range, it also provides a physically based framework for assessing additional heat capacity contributions. Moreover, the VDE model provides comparable (if not better) accuracy than DFT results at

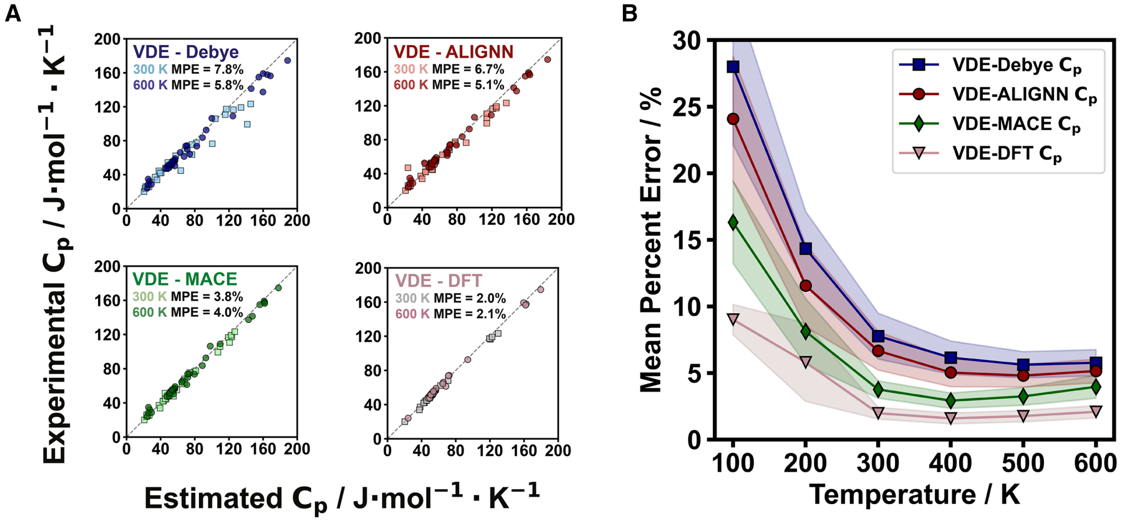

Figure 3. Comparison of VDE heat capacity estimates with different phonon density of states inputs

(A) The VDE estimate using the Debye phonon density of states is found to have the highest mean percent error (MPE) values, with MPE of 7.8% at 300 K (light blue squares) and 5.8% at 600 K (dark blue circles). The VDE estimate using the ALIGNN phonon density of states is found to improve upon the former, with MPE values of 6.7% at 300 K (light red squares) and 5.1% at 600 K (dark red circles). When the MACE model is used as the phonon density of states input, we find the MPE to be 3.8% at 300 K (light green squares) and 4.0% at 600 K (dark green circles). VDE estimates using DFT phonon density of states inputs have very low MPE values, with the outputs (light gray squares) having MPE values of 2.0% at 300 K and 2.1% at 600 K (pink circles).

(B) The MPE was assessed as a function of temperature for VDE outputs with phonon density of states estimations from the Debye model (blue squares), ALIGNN model (red circles), MACE model (green diamonds), and DFT (pink triangles). The VDE model's dilation and electronic terms contribute to lower MPE values for all models (Notes S2 and S3). Shaded regions represent the standard error of the mean at each temperature.

temperatures above 300 K, yet it can be run in minutes and does not require supercomputer usage. Because the VDE model requires much of the same input information as the Debye model, there is a low barrier to using the VDE model for improved heat capacity estimates (Note S1).

Although the ALIGNN density of states estimation is featured in the VDE model (see Figure 2), it is important to note the earlier point that any phonon density of states estimation could be used in its place. To exemplify this and examine the effects of phonon density of states estimates in the VDE model, we compare the MPEs of VDE outputs that use the analytical Debye model, MACE model, and DFT-derived density of states in Equation 8 (Figure 3).

To clarify, the Debye and Dulong-Petit values featured in Figure 2 follow textbook definitions[31] that do not consider dilation or electronic terms. The DFT values in Figure 2 include DFT-calculated dilation and electronic terms. In Figure 3, all results utilize the dilation and electronic terms defined for the VDE model.

An important conclusion from this analysis of the VDE model capabilities (Figure 3) is that both the ALIGNN and MACE estimates of the phonon density of states result in heat capacity values (MPE ~5%) that rival DFT determinations (as seen in Figure 2). A large contributing factor is the analytical estimate of the thermal expansion coefficient used in the dilation term (Equation 9 and Note S2), which also drastically improves heat capacity estimates when using DFT-determined phonon density of states (MPE ~2% in Figure 3; see also Note S3). Considering the computing resources needed for DFT, machine-learned estimates for density of states provide an excellent alternative. Although the Debye density of states performs comparably in this analysis, we will show that this does not hold for specific examples of complex materials.

HEAT CAPACITY OF COMPLEX MATERIALS

To better understand the scope of applicability and limitations of the VDE model, specific analysis was undertaken for a range of energy materials. Here, we use the ALIGNN estimate of the phonon density of states with comparison to the MACE model in places. For a complete comparison, see Note S7.

The temperature-dependent heat capacity of Mg_3Sb_2 and Si was evaluated and compared with experimental results (Figure 4; Note S4). The compound Mg_3Sb_2 is an exceptional n-type thermoelectric material that is the first to truly rival Bi_2Te_3 in the low-mid temperature range (~300–500 K)[47] and consequently has received a lot of research attention over the past decade.[48] Because the heat capacity of Mg_3Sb_2 has been theoretically and experimentally evaluated in detail previously,[24] this comparison also serves as a validation of the VDE model.

With both Mg_3Sb_2 and Si, the VDE model has good agreement with experimental values. The case of Mg_3Sb_2 (Figures 4A and 4B) particularly exemplifies the importance of the dilation term. In this material, the experimentally observed heat capacity is higher than the Dulong-Petit value at temperatures above ~300 K, which is not considered in models of C_V. When the dilation term is not included in the VDE model, it too underestimates the experimental values at high temperature (Note S5). In any case, the machine-learned phonon density of states estimation

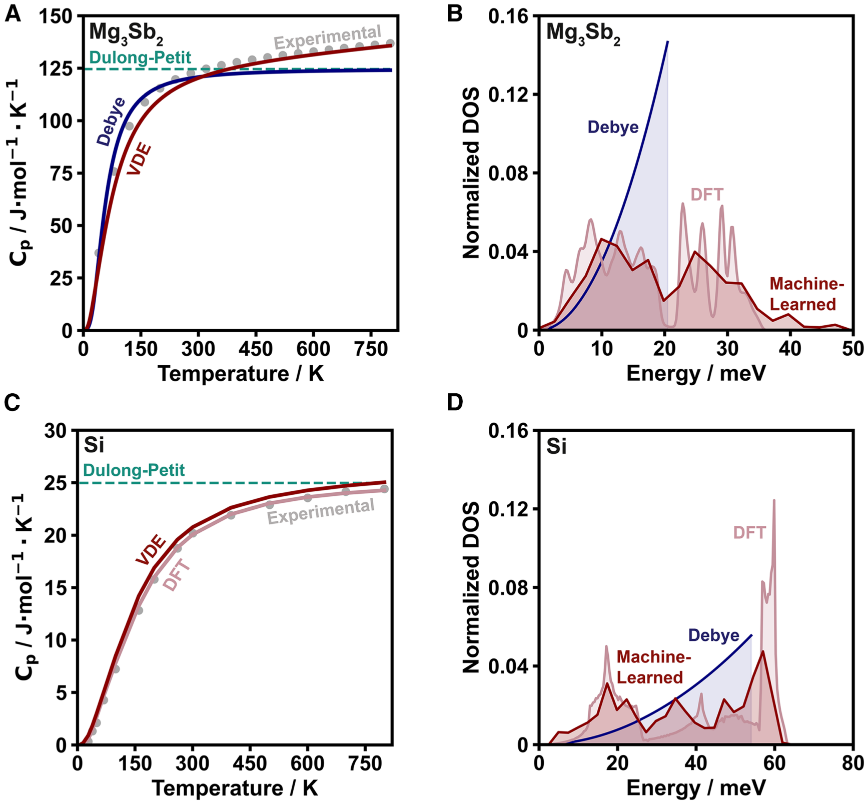

Figure 4. Temperature-dependent heat capacity and phonon density of states of Mg_3Sb_2 and Si

(A) Heat capacity estimations of Mg_3Sb_2 as a function of temperature with the Dulong-Petit (teal dotted line), Debye (blue), and VDE (red) models compared with experimental values[24] (gray points).

(B) Normalized phonon density of states estimates as a function of phonon energy $(E = \hbar\omega)$ for Mg_3Sb_2 using the Debye model (blue), the ALIGNN algorithm from Gurunathan et al.[35] (red), and using DFT with phonopy[49,50] (pink), previously reported.[24]

(C) Heat capacity estimations of Si as a function of temperature with the Dulong-Petit (teal dotted line), DFT (pink), and VDE (red) models compared with experimental values[30] (gray points).

(D) Normalized phonon density of states estimates as a function of phonon energy for Si using the Debye model (blue), the ALIGNN algorithm from Gurunathan et al.[35] (red), and using DFT with phonopy[49,50] (pink).

captures more spectral features than the Debye model (Figure 4B). In addition to using the VDE model for Si, heat capacity was also estimated using DFT-determined phonon density of states and thermal expansion coefficient in Equation 8 (Figures 4C and 4D; Note S3). While the DFT-based estimation of heat capacity is somewhat closer to experimental values, the VDE model using a machine-learned phonon density of states estimate and harmonic approximation of the thermal expansion coefficient is also in good agreement. Thus, although more accurate estimates of phonon density of states and material properties may be incorporated into heat capacity models (e.g., Equation 3), this study highlights the capability of incorporating machine-learned inputs into physics-based models to provide better heat capacity estimates than analytical models alone can provide.

To test its applicability across a wide temperature range, $LiCoO_2$, an archetype battery cathode material,[51] and alumina (Al_2O_3), a common high-temperature ceramic,[52] were investigated (Figure 5; Note S4). For both materials, the VDE model more closely follows experimentally determined heat capacity values than the Debye model and maintains its accuracy even at the highest temperatures (above 2,000 K in the case of alumina). Although the Dulong-Petit and Debye models capture the high-temperature limit of C_V, the full contributions to C_p are captured in the VDE model, displaying the strength of the VDE model in accounting for dilation contributions at high temperature. At low temperature, the more detailed vibrational density of states contributes to closer predictions of experimental heat capacity. This is especially important considering that $LiCoO_2$ is primarily used in batteries around room temperature where the heat capacity has a strong temperature dependence.

Because the ALIGNN model relies on the atomic structure to estimate the phonon density of states, it is appropriate to question what the effect of structural complexity may be on VDE model estimates. For this assessment, amorphous silicon (a-Si), a well-known MOF material known as ZIF-8,[59] and the polymer polyvinyl chloride (PVC) were investigated (Figure 6; Notes S4 and S6). Given that the ALIGNN machine-learned phonon density of states estimation was trained on relatively small unit cell crystalline structures, it is not surprising that the VDE model closely follows experimental values of a material like $LiCoO_2$ (Figure 5A). Interestingly, however, a-Si (Figure 6A), which is far outside this training in terms of its number of atoms and non-crystalline nature,[60] is still reasonably predicted by the VDE model, especially considering the discontinuity between the low-temperature and high-temperature data.[61,62] In the cases of ZIF-8 and PVC, the heat capacity is somewhat overestimated by the VDE model compared with experimental values (Figures 6B and 6C) resulting from an overestimation of the low-frequency (<120 meV) vibrational density of states. Recognizing that there are vibrational modes in ZIF-8 and PVC at energies higher than 120 meV (Note S6), the apparent normalized density of states from the ALIGNN model is clearly overestimated. Still, the Debye and Dulong-Petit models fail drastically in describing the temperature dependence and magnitude of heat capacity in ZIF-8 and PVC up to ~300 K.

The high-frequency vibrations in ZIF-8 are better handled by the MACE force field model, but given that the heat capacity is underestimated (see VDE* in Figure 6B), this indicates that the frequencies of the vibrational modes are overestimated on average (Note S6). For the case of PVC, however, the MACE

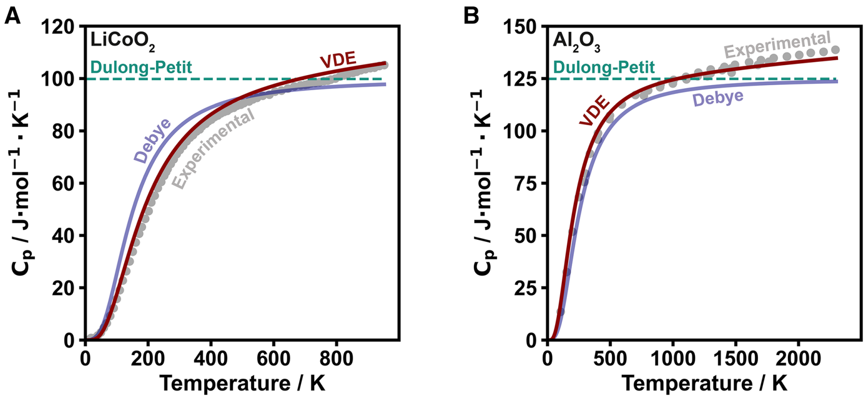

Figure 5. Heat capacity estimations of $LiCoO_2$ and Al_2O_3

(A) Heat capacity of $LiCoO_2$ as a function of temperature with the Dulong-Petit (teal dotted line), Debye (blue), and VDE (red) models compared with experimental values[53,54] (gray points).

(B) Heat capacity of Al_2O_3 as a function of temperature with the Dulong-Petit (teal dotted line), Debye (blue), and VDE (red) models compared with experimental values (gray points).[55–58]

model provides an exceptional estimation for the vibrational modes, providing a much-improved estimate for the heat capacity (see VDE* in Figure 6C). Thus, while the VDE model using the ALIGNN estimate of the phonon density of states provides the least complicated assessment of heat capacity, the use of machine-learned force fields can be used without much additional complexity.

While the 38 test materials (Figure 2) demonstrate that the VDE model performs well for structures within the scope of the ALIGNN model training, the specific example materials (Figures 5 and 6; Notes S4–S7) illustrate that the VDE model may even be applied to materials outside that scope. Further improvements may still be attained by developing machine-learned models for the phonon density of states that explicitly consider large unit cells and complex crystals, as well as amorphous materials. Nevertheless, the VDE model represents the possible advantage of incorporating machine-learned results into physics-based models instead of using machine learning alone[65] and provides benchmarking for different machine-learned models. Further, the dilation term from phonon pressure theory excels in this analysis. As thermodynamic assessments and transport models for engineering devices are increasingly important for the development of next-generation technologies, accessible estimates of heat capacity play a foundational role.

HEAT CAPACITY DURING PHASE TRANSFORMATIONS

Phase transformations are ubiquitous in engineering materials. Although many phase transformations are the result of a change in the thermodynamic free energy landscape with temperature or pressure, changes in elemental composition also initiate phase transformations. For example, battery materials used for the anode and cathode often undergo phase transformations during charge/discharge cycling due to changing local concentrations of the mobile ion (e.g., Li^+). Reported changes to the thermoelectric properties of materials undergoing phase transformations have led to controversial claims of increased thermoelectric figure of merit.[19] Other specific phase transitions, such as spin-crossover in MOF materials[66] or those in chalcogenide memory materials,[67] are also gaining increasing attention with respect to the material's thermal properties. More generally, characterizing and understanding the impact of phase transformations on thermal properties are foundational to engineering thermal management in modern materials systems.

Phase transformations contribute to heat capacity when they store thermal energy by maintaining equilibrium in the system. The total heat capacity of a multi-phase system is the sum of the intrinsic heat capacity $C_{p\phi}$ of the phases present (e.g., Equation 3) and the contribution from the enthalpy of transformation ΔH as (Note S9)

$$C_p = C_{p\phi} + \Delta H\left(\frac{\partial\phi}{\partial T}\right)_p \quad \text{(Equation 10)}$$

when changes in equilibrium (characterized by the order parameter ϕ) are able to respond "instantly" to changes in temperature. More generally, when ϕ does not respond instantly to changes in temperature, the relation[68]

$$C_p = C_{p\phi} + \Delta H\frac{(\partial\phi/\partial t)_p}{(\partial T/\partial t)_p} \quad \text{(Equation 11)}$$

can be used, which considers the respective changes of ϕ and T with time t. Nevertheless, Equation 10 provides a valuable starting point, as $(\partial\phi/\partial T)_p$ can be estimated directly from the equilibrium phase diagram using the inverse lever rule (Figures 7A and 7B). Microscopically, the additional contribution to the heat capacity arises from the additional degree(s) of freedom facilitated by having a dynamic interface between the equilibrium phases. This is to say that thermal energy is stored in the fluctuations in ϕ at equilibrium.

For materials where phase transformation contributions to the heat capacity are observed experimentally, Equation 10 is reasonably expected to explain the temperature dependence of the heat capacity. Here, an estimation of the heat capacity of Cu_2Se from ~2–575 K, through the $\alpha \rightarrow \beta$ phase transition, was achieved using the VDE model (Equation 8) as $C_{p\phi}$ in Equation 10 (Figure 7C; Notes S4 and S9). The $(\partial\phi/\partial T)_p$ term was estimated using the phase diagram of Cu_2Se,[19,69–71] and ΔH was considered to be temperature independent with a value of 30 $J{\cdot}g^{-1}$.[69] Despite the additional complexity contributed by

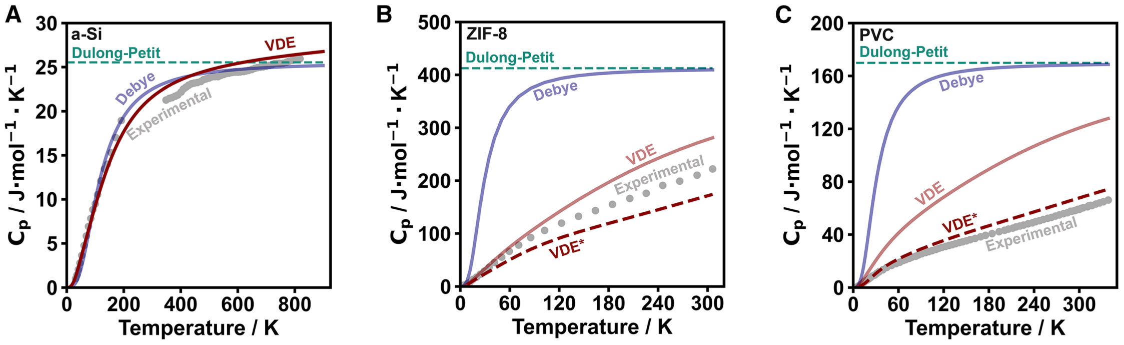

Figure 6. Heat capacity estimations of a-Si, ZIF-8, and PVC

(A) Heat capacity of a-Si as a function of temperature with the Dulong-Petit (teal dotted line), Debye (blue), and VDE (red) models compared with experimental values[61,62] (gray points).

(B) Heat capacity of ZIF-8 as a function of temperature with the Dulong-Petit model (teal dotted line), Debye model (blue), VDE model using ALIGNN (pink), and VDE model using MACE (red dotted, Note S6). Models are compared with experimental values for ZIF-8[63] (gray points).

(C) Heat capacity of PVC as a function of temperature with the Dulong-Petit model (teal dotted line), Debye model (blue), VDE model using ALIGNN (pink), and VDE model using MACE (red dotted, Note S6). Models are compared with experimental values for PVC[64] (gray points).

the phase transformation region, there is excellent agreement of the heat capacity estimation with experimental values across the full temperature range (Figure 7C).

It should be emphasized that an accurate characterization of the phase diagram (in the vicinity of the phase transformation of interest) is a requisite step for the characterization of the heat capacity using Equation 10. This is because the specific values of $(\partial\phi/\partial T)_p$ will depend on the exact composition and the phase diagram. The time considerations of Equation 11 suggest that the apparent heat capacity at a given temperature could be somewhat less than that predicted by Equation 10. For example, thermal lag introduced inherently by the heating/cooling rate of experimental characterization techniques may affect measured heat capacity values at a given temperature. Consequently, a detailed study of transformation kinetics (as a function of time and temperature) may be necessary to explain phase transformation contributions to heat capacity measurements in some materials. Previous efforts[75,76] to characterize phase transformation contributions to heat capacity from a kinetics perspective underline the importance of transformation "speed" but seemingly overlook the fundamental importance of composition and equilibrium phase relations in describing the heat capacity of multi-phase systems.

In the thermodynamic relation between thermal conductivity and thermal diffusivity (Equation 1), it is the total heat capacity (including phase transformation contributions) that should be used. Thus, characterizing heat capacity through phase transformations is vital to assessing thermal conductivity in these temperature regions. Concurrently, understanding the timescales of transformation kinetics, as well as measurement rates and thermal lag, is necessary for proper analysis of heat capacity data. Especially as considerations of thermal management in electrochemical devices, like solid-state batteries, become increasingly prominent, the ability to characterize/estimate heat and temperature evolution throughout dynamic processes will be beneficial.

OUTLOOK

Accurate characterization of material thermal properties is a fundamental step toward systems-level design capabilities. The importance of heat capacity, both for defining the thermodynamic state of a material and for estimating thermal conductivity, means that it should be thoroughly studied in its own right. State-of-the-art thermodynamics modeling, such as CALPHAD, requires estimates of heat capacity for predicting thermodynamic phase relations. Combined with high-throughput first-principles structure calculations, the computational design of materials is expected to rapidly accelerate technological innovation. Accurate estimates of thermal conductivity, especially in complex systems undergoing phase transformations, will be essential to device design. For example, heat capacity estimations in the design of battery architectures and the optimization of charging rates could aid in the effort to mitigate material degradation and improve battery safety. Likewise, accurate estimations of thermoelectric performance are essential for identifying materials with promising real-world applications.

In totality, the VDE model provides a straightforward estimate for heat capacity at temperatures relevant for engineering design with DFT-level accuracy. Including the dilation term to C_p using the thermal expansion coefficient determined from phonon pressure theory allows for a description of heat capacity based solely on the structural and elastic properties of a solid. Estimates fall within ~7% of experimental values from 300 to 600 K and are at least 29% more accurate than the Debye model on average. When DFT-based phonon density of states inputs are used in the VDE model, estimates fall within ~2% of experimental values. Thus, the VDE model serves as both a high-throughput metric for heat capacity values and as an excellent starting point for more detailed heat capacity analysis. By also demonstrating how to include the contribution from phase transformations into a heat capacity model, this work provides a hierarchical perspective for assessing the heat capacity of complex materials. Toward this end, future work to incorporate

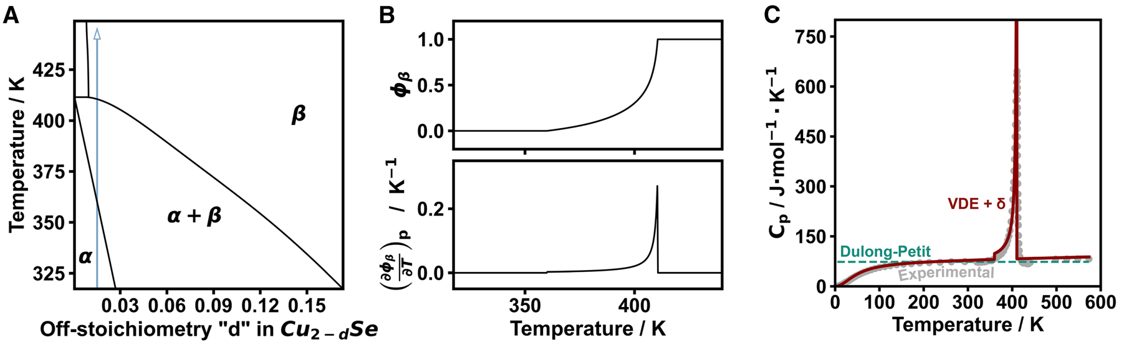

Figure 7. Heat capacity of Cu_2Se through a phase transition estimated using the VDE model and the equilibrium phase diagram

(A) A portion of the Cu-Se phase diagram depicting the $\alpha \rightarrow \beta$ phase transition of $Cu_{2-d}Se$.[69–71]

(B) The equilibrium phase fraction ϕ_β of the high-temperature β phase and its temperature derivative $(\partial\phi_\beta/\partial T)_p$ are shown as a function of temperature for $Cu_{1.985}Se$ (corresponding to the blue arrow in A).[19]

(C) Heat capacity estimations of Cu_2Se with the Dulong-Petit model (teal dotted line) and the VDE model using ALIGNN with the added phase change contribution denoted by δ, where $\delta = \Delta H(\partial\phi_\beta/\partial T)_p$ (red). Models are compared with experimental values[72–74] (gray points). The discontinuity in the VDE C_p curve at the onset and conclusion of the phase transition is due to the numerical aspect of calculating $(\partial\phi_\beta/\partial T)_p$ without consideration of thermal broadening (Note S8). In effect, going from the α to $\alpha + \beta$ region happens in a stepwise manner, leading to the apparent discontinuity.

machine-learning results into physics-based models may lead to further improvements in estimations of heat capacity and other thermophysical properties needed for modern materials design.

DATA AND CODE AVAILABILITY

The data and code necessary to reproduce the findings of this study are available on Github (https://github.com/AgneLab/Heat-Capacity) and in the supplemental information found online at https://doi.org/10.1016/j.joule.2025.102054.

ACKNOWLEDGMENTS

E.J.C. and M.T.A. would like to acknowledge Research Advanced Computing Services (RACS) at the University of Oregon for providing computing resources that have contributed to the research results reported within this publication (https://racs.uoregon.edu). J.B. and J.G. would like to acknowledge the Gauss Centre for Supercomputing e.V. (https://www.gauss-centre.eu) for funding workflow-related developments by providing generous computing time on the GCS Supercomputer SuperMUC-NG at Leibniz Supercomputing Centre (http://www.lrz.de) (project pn73da). J.G. was supported by MultiBonds ERC Grant (grant agreement no. 101161771) funded by the European Union. Views and opinions expressed are, however, those of the author (s) only and do not necessarily reflect those of the European Union or the European Research Council Executive Agency. Neither the European Union nor the granting authority can be held responsible for them.

AUTHOR CONTRIBUTIONS

Conceptualization, M.T.A.; methodology, E.J.C. and M.T.A.; VDE model coding, E.J.C.; coding support, Z.M.J., R.G., J.G., and M.T.A.; writing – original draft, E.J.C. and M.T.A.; writing – review and editing, E.J.C. and all authors; compiling material information, E.J.C., Z.M.J., A.L., J.B., and M.T.A.; DFT and MACE calculations, J.B., E.J.C., and J.G.; additional resources, R.G., J. G., and M.T.A.; supervision, M.T.A..

DECLARATION OF INTERESTS

The authors declare no competing interests.

SUPPLEMENTAL INFORMATION

Supplemental information can be found online at https://doi.org/10.1016/j.joule.2025.102054.

REFERENCES

1. Meng, Y., Pu, J., and Pei, Q. (2021). Electrocaloric cooling over high device temperature span. Joule *5*, 780–793. https://doi.org/10.1016/j.joule.2020.12.018.
2. Wang, Y., Wu, X., Yu, M., Shen, X., Wang, S., Li, H., Zhang, Z., and Liu, W. (2024). Thermoelectric cyclic-thermal regulation: A new operational mode of thermoelectric materials with high energy efficiency. Joule *8*, 3201–3216. https://doi.org/10.1016/j.joule.2024.08.002.
3. Clarke, D.R., Oechsner, M., and Padture, N.P. (2012). Thermal-barrier coatings for more efficient gas-turbine engines. MRS Bull. *37*, 891–898. https://doi.org/10.1557/mrs.2012.232.
4. Snyder, G.J. (2004). Application of the compatibility factor to the design of segmented and cascaded thermoelectric generators. Appl. Phys. Lett. *84*, 2436–2438. https://doi.org/10.1063/1.1689396.
5. Agne, M.T., Böger, T., Bernges, T., and Zeier, W.G. (2022). Importance of thermal transport for the design of solid-state battery materials. PRX Energy *1*, 031002. https://doi.org/10.1103/PRXEnergy.1.031002.
6. Feng, X., Ren, D., He, X., and Ouyang, M. (2020). Mitigating thermal runaway of lithium-ion batteries. Joule *4*, 743–770. https://doi.org/10.1016/j.joule.2020.02.010.
7. Rui, X., Ren, D., Liu, X., Wang, X., Wang, K., Lu, Y., Li, L., Wang, P., Zhu, G., Mao, Y., et al. (2023). Distinct thermal runaway mechanisms of sulfide-based all-solid-state batteries. Energy Environ. Sci. *16*, 3552–3563. https://doi.org/10.1039/D3EE00084B.
8. Bates, A.M., Preger, Y., Torres-Castro, L., Harrison, K.L., Harris, S.J., and Hewson, J. (2022). Are solid-state batteries safer than lithium-ion batteries? Joule *6*, 742–755. https://doi.org/10.1016/j.joule.2022.02.007.
9. Kong, J., Chung, S.W., and Skadron, K. (2012). Recent thermal management techniques for microprocessors. ACM Comput. Surv. *44*, 1–42. https://doi.org/10.1145/2187671.2187675.
10. Diaconu, B.M., Cruceru, M., and Anghelescu, L. (2023). A critical review on heat transfer enhancement techniques in latent heat storage systems based on phase change materials. passive and active techniques, system

designs and optimization. J. Energy Storage *61*, 106830. https://doi.org/10.1016/j.est.2023.106830.

11. Schleich, W.P., Ranade, K.S., Anton, C., Arndt, M., Aspelmeyer, M., Bayer, M., Berg, G., Calarco, T., Fuchs, H., Giacobino, E., et al. (2016). Quantum technology: from research to application. Appl. Phys. B *122*, 130. https://doi.org/10.1007/s00340-016-6353-8.
12. Salomão, R., Oliveira, K., Fernandes, L., Tiba, P., and Prado, U. (2022). Porous refractory ceramics for high-temperature thermal insulation - part 2: The technology behind energy saving. Interceram. – Int. Ceram. Rev. *71*, 38–50. https://doi.org/10.1007/s42411-022-0483-2.
13. Lan, D., and Green, M.A. (2022). Combatting temperature and reverse-bias challenges facing perovskite solar cells. Joule *6*, 1782–1797. https://doi.org/10.1016/j.joule.2022.06.014.
14. Park, S., Kim, T., Yoon, S., Koh, C.W., Woo, H.Y., and Son, H.J. (2020). Progress in materials, solution processes, and long-term stability for large-area organic photovoltaics. Adv. Mater. *32*, e2002217. https://doi.org/10.1002/adma.202002217.
15. Rosen, P.F., Dickson, M.S., Calvin, J.J., Ross, N.L., Friščić, T., Navrotsky, A., and Woodfield, B.F. (2020). Thermodynamic evidence of structural transformations in CO2 -loaded metal–organic framework Zn(Melm)2 from heat capacity measurements. J. Am. Chem. Soc. *142*, 4833–4841. https://doi.org/10.1021/jacs.9b13883.
16. Snyder, G.J., and Toberer, E.S. (2008). Complex thermoelectric materials. Nat. Mater. *7*, 105–114. https://doi.org/10.1038/nmat2090.
17. Grimvall, G. (1999). Thermophysical properties of materials (Elsevier).
18. Aftab, W., Usman, A., Shi, J., Yuan, K., Qin, M., and Zou, R. (2021). Phase change material-integrated latent heat storage systems for sustainable energy solutions. Energy Environ. Sci. *14*, 4268–4291. https://doi.org/10.1039/D1EE00527H.
19. Agne, M.T., Voorhees, P.W., and Snyder, G.J. (2019). Phase transformation contributions to heat capacity and impact on thermal diffusivity, thermal conductivity, and thermoelectric performance. Adv. Mater. *31*, e1902980. https://doi.org/10.1002/adma.201902980.
20. Ritz, E.T., Li, S.J., and Benedek, N.A. (2019). Thermal expansion in insulating solids from first principles. J. Appl. Phys. *126*, 171102. https://doi.org/10.1063/1.5125779.
21. Blaine, R.L. (2018). In search of thermal effusivity reference materials. J. Therm. Anal. Calorim. *132*, 1419–1422. https://doi.org/10.1007/s10973-018-7020-6.
22. Agne, M.T., and Barsoum, M.W. (2016). Enthalpy of formation and thermodynamic parameters of the max phase v2alc. J. Alloys Compd. *665*, 218–224. https://doi.org/10.1016/j.jallcom.2015.12.204.
23. Olson, G.B., and Liu, Z.K. (2023). Genomic materials design: Calculation of phase dynamics. Calphad *82*, 102590. https://doi.org/10.1016/j.calphad.2023.102590.
24. Agne, M.T., Imasato, K., Anand, S., Lee, K., Bux, S.K., Zevalkink, A., Rettie, A.J.E., Chung, D.Y., Kanatzidis, M.G., and Snyder, G.J. (2018). Heat capacity of mg3sb2, mg3bi2, and their alloys at high temperature. Mater. Today Phys. *6*, 83–88. https://doi.org/10.1016/j.mtphys.2018.10.001.
25. Agne, M.T., Hanus, R., and Snyder, G.J. (2018). Minimum thermal conductivity in the context of diffuson -mediated thermal transport. Energy Environ. Sci. *11*, 609–616. https://doi.org/10.1039/C7EE03256K.
26. Bernges, T., Peterlechner, M., Wilde, G., Agne, M.T., and Zeier, W.G. (2023). Analytical model for two-channel phonon transport engineering. Mater. Today Phys. *35*, 101107. https://doi.org/10.1016/j.mtphys.2023.101107.
27. Chen, Z., Zhang, X., Lin, S., Chen, L., and Pei, Y. (2018). Rationalizing phonon dispersion for lattice thermal conductivity of solids. Natl. Sci. Rev. *5*, 888–894. https://doi.org/10.1093/nsr/nwy097.
28. Moon, J., Zella, L., and Lindsay, L. (2024). Collective nature of phonon energies beyond harmonic oscillators. Comp. Mater. Today *4*, 100023. https://doi.org/10.1016/j.commt.2024.100023.
29. Borup, K.A., De Boor, J., Wang, H., Drymiotis, F., Gascoin, F., Shi, X., Chen, L., Fedorov, M.I., Müller, E., Iversen, B.B., and Snyder, G.J. (2015). Measuring thermoelectric transport properties of materials. Energy Environ. Sci. *8*, 423–435. https://doi.org/10.1039/C4EE01320D.
30. Porter, L.J., Yip, S., Yamaguchi, M., Kaburaki, H., and Tang, M. (1997). Empirical bond-order potential description of thermodynamic properties of crystalline silicon. J. Appl. Phys. *81*, 96–106. https://doi.org/10.1063/1.364102.
31. Kittel, C. (2005). Introduction to Solid State Physics, Eight edition. (John Wiley & Sons, Inc).
32. Ridier, K., Zhang, Y., Piedrahita-Bello, M., Quintero, C.M., Salmon, L., Molnár, G., Bergaud, C., and Bousseksou, A. (2020). Heat capacity and thermal damping properties of spin-crossover molecules: A new look at an old topic. Adv. Mater. *32*, e2000987. https://doi.org/10.1002/adma.202000987.
33. Batatia, I., Benner, P., Chiang, Y., Elena, A.M., Kovács, D.P., Riebesell, J., Advincula, X.R., Asta, M., Avaylon, M., Baldwin, W.J., et al. (2024). A foundation model for atomistic materials chemistry. Preprint at arXiv. https://doi.org/10.48550/arXiv.2401.00096.
34. Yang, H., Hu, C., Zhou, Y., Liu, X., Shi, Y., Li, J., Li, G., Chen, Z., Chen, S., Zeni, C., et al. (2024). Mattersim: A deep learning atomistic model across elements, temperatures and pressures. Preprint at arXiv. https://doi.org/10.48550/arXiv.2405.04967.
35. Gurunathan, R., Choudhary, K., and Tavazza, F. (2023). Rapid prediction of phonon structure and properties using the atomistic line graph neural network (alignn). Phys. Rev. Mater. *7*, 023803. https://doi.org/10.1103/PhysRevMaterials.7.023803.
36. Choudhary, K., Garrity, K.F., Reid, A.C.E., DeCost, B., Biacchi, A.J., Hight Walker, A.R., Trautt, Z., Hattrick-Simpers, J., Kusne, A.G., Centrone, A., et al. (2020). The joint automated repository for various integrated simulations (jarvis) for data-driven materials design. Computational Materials *6*, 173. https://doi.org/10.1038/s41524-020-00440-1.
37. Choudhary, K., and Tavazza, F. (2019). Convergence and machine learning predictions of monkhorst-pack k-points and plane-wave cut-off in high-throughput dft calculations. Comp. Mater. Sci. *161*, 300–308. https://doi.org/10.1016/j.commatsci.2019.02.006.
38. Chen, Z., Andrejevic, N., Smidt, T., Ding, Z., Xu, Q., Chi, Y.T., Nguyen, Q.T., Alatas, A., Kong, J., and Li, M. (2021). Direct prediction of phonon density of states with euclidean neural networks. Adv. Sci. (Weinh) *8*, e2004214. https://doi.org/10.1002/advs.202004214.
39. Okabe, R., Chotrattanapituk, A., Boonkird, A., Andrejevic, N., Fu, X., Jaakkola, T.S., Song, Q., Nguyen, T., Drucker, N., Mu, S., et al. (2024). Virtual node graph neural network for full phonon prediction. Nat. Comput. Sci. *4*, 522–531. https://doi.org/10.1038/s43588-024-00661-0.
40. Kong, S., Ricci, F., Guevarra, D., Neaton, J.B., Gomes, C.P., and Gregoire, J.M. (2022). Density of states prediction for materials discovery via contrastive learning from probabilistic embeddings. Nat. Commun. *13*, 949. https://doi.org/10.1038/s41467-022-28543-x.
41. Choudhary, K., DeCost, B., Major, L., Butler, K., Thiyagalingam, J., and Tavazza, F. (2023). Unified graph neural network force-field for the periodic table: solid state applications. Digit. Discov. *2*, 346–355. https://doi.org/10.1039/D2DD00096B.
42. Grimme, S., Antony, J., Ehrlich, S., and Krieg, H. (2010). A consistent and accurate ab initio parametrization of density functional dispersion correction (dft-d) for the 94 elements h-pu. J. Chem. Phys. *132*, 154104. https://doi.org/10.1063/1.3382344.
43. Jain, A., Ong, S.P., Hautier, G., Chen, W., Richards, W.D., Dacek, S., Cholia, S., Gunter, D., Skinner, D., Ceder, G., and Persson, K.A. (2013). Commentary: The materials project: A materials genome approach to accelerating materials innovation. APL Mater. *1*, 011002. https://doi.org/10.1063/1.4812323.
44. Agne, M.T., Anand, S., and Snyder, G.J. (2022). Inherent anharmonicity of harmonic solids. Research (Wash D. C) *2022*, 9786705. https://doi.org/10.34133/2022/9786705.

45. Chen, Z., Liu, W., Shan, B., and Pei, Y. (2024). Analytical approach to structural chemistry origins of mechanical, acoustical and thermal properties. Natl. Sci. Rev. *11*, nwae269. https://doi.org/10.1093/nsr/nwae269.
46. Allison, T.C. (2013). NIST-JANAF Thermochemical Tables. https://janaf.nist.gov/.
47. Imasato, K., Kang, S.D., Ohno, S., and Snyder, G.J. (2018). Band engineering in mg 3 sb 2 by alloying with mg 3 bi 2 for enhanced thermoelectric performance. Mater. Horiz. *5*, 59–64. https://doi.org/10.1039/C7MH00865A.
48. Imasato, K., Wood, M., Anand, S., Kuo, J.J., and Snyder, G.J. (2022). Understanding the high thermoelectric performance of mg 3 Sb 2 -Mg3 Bi 2 alloys. Adv. Energy Sustain. Res. *3*, 2100208. https://doi.org/10.1002/aesr.202100208.
49. Togo, A. (2023). First-principles Phonon Calculations with Phonopy and Phono3py. J. Phys. Soc. Jpn. *92*, 012001. https://doi.org/10.7566/JPSJ.92.012001.
50. Togo, A., Chaput, L., Tadano, T., and Tanaka, I. (2023). Implementation strategies in phonopy and phono3py. J. Phys. Condens. Matter *35*, 353001. https://doi.org/10.1088/1361-648X/acd831.
51. Liu, Q., Su, X., Lei, D., Qin, Y., Wen, J., Guo, F., Wu, Y.A., Rong, Y., Kou, R., Xiao, X., et al. (2018). Approaching the capacity limit of lithium cobalt oxide in lithium ion batteries via lanthanum and aluminium doping. Nat. Energy *3*, 936–943. https://doi.org/10.1038/s41560-018-0180-6.
52. Wang, X., Zhong, Y., and Hu, Q. (2025). A review of al2o3-based eutectic ceramics for high-temperature structural materials. J. Mater. Sci. Technol. *214*, 214–232. https://doi.org/10.1016/j.jmst.2024.07.012.
53. Gotcu-Freis, P., Cupid, D.M., Rohde, M., and Seifert, H.J. (2015). New experimental heat capacity and enthalpy of formation of lithium cobalt oxide. J. Chem. Thermodyn. *84*, 118–127. https://doi.org/10.1016/j.jct.2014.12.007.
54. Kawaji, H., Takematsu, M., Tojo, T., Atake, T., Hirano, A., and Kanno, R. (2002). Low temperature heat capacity and thermodynamic functions of licoo2. Journal of Thermal Analysis and Calorimetry *68*, 833–839. https://doi.org/10.1023/A:1016169917912.
55. Huang, L.F., Lu, X.Z., Tennessen, E., and Rondinelli, J.M. (2016). An efficient ab-initio quasiharmonic approach for the thermodynamics of solids. Comp. Mater. Sci. *120*, 84–93. https://doi.org/10.1016/j.commatsci.2016.04.012.
56. Munro, R.G. (1997). Evaluated material properties for a sintered alpha-alumina. J. Am. Ceram. Soc. *80*, 1919–1928. https://doi.org/10.1111/j.1151-2916.1997.tb03074.x.
57. Schauer, A. (1965). Thermal expansion, grueneisen parameter, and temperature dependence of lattice vibration frequencies of aluminum oxide. Can. J. Phys. *43*, 523–531. https://doi.org/10.1139/p65-049.
58. Chase, M. (1998). NIST-JANAF Thermochemical Tables, Fourth Edition (American Institute of Physics), p. 1.
59. Lai, Z. (2018). Development of zif-8 membranes: opportunities and challenges for commercial applications. Curr. Opin. Chem. Eng. *20*, 78–85. https://doi.org/10.1016/j.coche.2018.03.002.
60. Deringer, V.L., Bernstein, N., Bartók, A.P., Cliffe, M.J., Kerber, R.N., Marbella, L.E., Grey, C.P., Elliott, S.R., and Csányi, G. (2018). Realistic atomistic structure of amorphous silicon from machine-learning-driven molecular dynamics. J. Phys. Chem. Lett. *9*, 2879–2885. https://doi.org/10.1021/acs.jpclett.8b00902.
61. Tsang, K.H., Kui, H.W., and Chik, K.P. (1993). Calorimetric studies of the heat capacity and relaxation of amorphous si prepared by electron beam evaporation. J. Appl. Phys. *74*, 4932–4935. https://doi.org/10.1063/1.354329.
62. Queen, D.R., Liu, X., Karel, J., Metcalf, T.H., and Hellman, F. (2013). Excess specific heat in evaporated amorphous silicon. Phys. Rev. Lett. *110*, 135901. https://doi.org/10.1103/PhysRevLett.110.135901.
63. Rosen, P.F., Calvin, J.J., Dickson, M.S., Katsenis, A.D., Friščić, T., Navrotsky, A., Ross, N.L., Kolesnikov, A.I., and Woodfield, B.F. (2019). Heat capacity and thermodynamic functions of crystalline forms of the metal-organic framework zinc 2-methylimidazolate, zn(meim)2. J. Chem. Thermodyn. *136*, 160–169. https://doi.org/10.1016/j.jct.2019.05.008.
64. Chang, S.S. (1977). Heat capacity and thermodynamic properties of poly (vinyl chloride). J. Res. Natl. Bur. Stand. (1977) *82*, 9–18. https://doi.org/10.6028/jres.082.002.
65. Kauwe, S.K., Graser, J., Vazquez, A., and Sparks, T.D. (2018). Machine learning prediction of heat capacity for solid inorganics. Integr. Mater. Manuf. Innov. *7*, 43–51. https://doi.org/10.1007/s40192-018-0108-9.
66. Davenport, A.M., Marshall, C.R., Nishiguchi, T., Kadota, K., Andreeva, A. B., Horike, S., and Brozek, C.K. (2024). Size-dependent spin crossover and bond flexibility in metal–organic framework nanoparticles. J. Am. Chem. Soc. *146*, 23692–23698. https://doi.org/10.1021/jacs.4c08883.
67. Zhang, W., Mazzarello, R., Wuttig, M., and Ma, E. (2019). Designing crystallization in phase-change materials for universal memory and neuro-inspired computing. Nat. Rev. Mater. *4*, 150–168. https://doi.org/10.1038/s41578-018-0076-x.
68. Prigogine, I., and Defay, R. (1954). Chemical Thermodynamics (Longmans, Green and Co Ltd).
69. Kang, S.D., Danilkin, S.A., Aydemir, U., Avdeev, M., Studer, A., and Snyder, G.J. (2016). Apparent critical phenomena in the superionic phase transition of cu 2- x se. New J. Phys. *18*, 013024. https://doi.org/10.1088/1367-2630/18/1/013024.
70. Ishikawa, T., and Miyatani, S.y. (1977). Electronic and ionic conduction in Cu 2-δ Se, Cu 2-δ S and Cu 2-δ (se, s). J. Phys. Soc. Jpn. *42*, 159–167. https://doi.org/10.1143/JPSJ.42.159.
71. Vučić, Z., Milat, O., Horvatić, V., and Ogorelec, Z. (1981). Composition-induced phase-transition splitting in cuprous selenide. Phys. Rev. B *24*, 5398–5401. https://doi.org/10.1103/PhysRevB.24.5398.
72. Liu, H., Shi, X., Xu, F., Zhang, L., Zhang, W., Chen, L., Li, Q., Uher, C., Day, T., and Snyder, G.J. (2012). Copper ion liquid-like thermoelectrics. Nat. Mater. *11*, 422–425. https://doi.org/10.1038/nmat3273.
73. Liu, H., Yang, J., Shi, X., Danilkin, S.A., Yu, D., Wang, C., Zhang, W., and Chen, L. (2016). Reduction of thermal conductivity by low energy multi-einstein optic modes. J. Materiomics *2*, 187–195. https://doi.org/10.1016/j.jmat.2016.05.006.
74. Brown, D.R., Heijl, R., Borup, K.A., Iversen, B.B., Palmqvist, A., and Snyder, G.J. (2016). Relating phase transition heat capacity to thermal conductivity and effusivity in cu 2 se. Physica Rapid. Research. Ltrs. *10*, 618–621. https://doi.org/10.1002/pssr.201600160.
75. Chen, H., Yue, Z., Ren, D., Zeng, H., Wei, T., Zhao, K., Yang, R., Qiu, P., Chen, L., and Shi, X. (2019). Thermal conductivity during phase transitions. Adv. Mater. *31*, e1806518. https://doi.org/10.1002/adma.201806518.
76. Qiu, X., Qiu, P., Yue, Z., Chen, H., Deng, T., Xiao, J., Ren, D., Zhou, Z., Chen, L., and Shi, X. (2022). Phase transition behaviors and thermoelectric properties of CuAgTe1–xSex near 400 k. ACS Appl. Mater. Interfaces *14*, 1015–1023. https://doi.org/10.1021/acsami.1c20333.

Joule, Volume *9*

Supplemental information

Heat capacity estimation of complex materials for energy technologies

Elana J. Cope, Joana Bustamante, Zöe M. Johnson, Alicia Lancaster, Ramya Gurunathan, Janine George, and Matthias T. Agne

Supplemental Note 1:

The VDE model code, related documents, and examples are provided at https://github.com/AgneLab/Heat-Capacity/, and example code can also be found below.

To run the VDE model, follow the following steps.

1. Ensure that Python is installed. Install alignn, pandas, numpy, matplotlib, scipy, pymatgen using pip installer.
2. Obtain a csv file with energy in meV and phonon density of states. This can be done by running the ALIGNN model code (https://github.com/usnistgov/alignn) or MACE (see below) on a provided CIF file using the following command:

run ALIGNN_DOS_Generate.py --model_name jv_pdos_alignn --file_format cif --file_path "./Compound.cif" --out_file "Compound.csv"

3. Obtain the following information for the compound: number of atoms per unit cell, density (gram per cubic cm), volume of cell (cubic Angstrom), bulk modulus (GPa), shear modulus (Gpa), molecular weight (g per mole). These can often be obtained from materials project[1] and loaded into the code via a csv file with inputs or using an API (https://api.materialsproject.org/docs). The number of atoms and the volume must be either both for the conventional cell or both for the primitive cell—do not mix and match.
4. If there is not a value for the electronic density of states at the Fermi level, just input 0 into the function for g_Ef. Otherwise, the electronic density of states at the Fermi level must be obtained using our function, which normalizes it into units of states per mole per cubic meter. The function takes the materials project ID and uses an API to obtain the full density of states, normalizes it, and finds the value at the Fermi level. Do not use the raw value of electronic density of states from Materials Project as the units will be incorrect.
5. Run the VDE model function with the energy and density of states values (e_raw_meV, g_raw), bulk modulus, shear modulus, density, molecular weight, and number of atoms from step 3 (B_vrh, G_vrh, Density, Molec_Wt, N_atom, V_cell). Follow step for the electronic density of states at the Fermi level (g_Ef), and define a temperature range (Temp).

To calculate the density of states using the MACE-MP-0-3b model, the phonon density of states with MACE-MP-0-3b[2] and D3 dispersion[3] correction can be computed. To perform the finite difference method, a Phonopy-based workflow[4,5] can be used, as implemented in atomate2.[6]

The code is shown below, with an example function that runs the model, saves the output into a csv, and plots the outputs.

```
######################################################################

import csv

import numpy as np

import matplotlib.pyplot as plt

from scipy.constants import hbar, k, e, pi

import pandas as pd
```

```
from matplotlib import rcParams, cm
from matplotlib.ticker import MaxNLocator
import os
from pymatgen.ext.matproj import MPRester
from collections import Counter
from pymatgen.core.periodic_table import Element  # To get atomic number
import time
import random

#figure settings
rcParams.update({'figure.autolayout': True}) #keeps the axis labels from getting cut off
# These commands modify default figure properties to make them more legible
plt.rcParams['axes.linewidth']    = 2.5
plt.rcParams['lines.linewidth']   = 1.5
plt.rcParams['font.sans-serif']   = 'Arial'
plt.rcParams['font.weight']       = 'bold'
plt.rcParams['axes.labelweight']  = 'bold'
plt.rcParams['axes.titleweight']  =    'bold'
plt.rcParams['axes.labelsize']    = 16
plt.rcParams['font.size'] = 18
plt.rcParams['axes.titlesize']    = 16
plt.rcParams['xtick.labelsize']   = 16
plt.rcParams['ytick.labelsize']  =  16
#plt.rcParams['axes.grid']        = True
plt.rcParams['xtick.major.size']  = 5
plt.rcParams['xtick.major.width'] = 2.5
plt.rcParams['ytick.major.size']  = 5
plt.rcParams['ytick.major.width'] = 2.5
plt.rcParams['lines.markersize']  = 12
plt.rcParams['xtick.minor.visible'] = False
plt.rcParams['ytick.minor.visible'] = False

#%%
```

```
def Cp_model(e_raw_meV, g_raw, B_vrh, G_vrh, Density, Molec_Wt, N_atom, V_cell, g_Ef, Temp):

    DF = pd.DataFrame(columns=['meV','DOS'])
    DF['meV']=pd.Series(e_raw_meV)
    DF['DOS']=pd.Series(g_raw)

    # delete all indices with negative energy using a boolean mask
    mask = DF.values[:, 0] >= 0 # energy in column 0
    pos_dataset = DF.values[mask]

    # replace any negative DOS values with 0
    pos_dataset[pos_dataset[:, 1] < 0, 1] = 0

    e_pos = pos_dataset[:,0]/6.241506363094e+21 # filtered energies converted from meV to J
    g_pos= pos_dataset[:,1] #filtered DOS (negative = 0)

    # normalize the DOS
    g_norm = g_pos/np.trapz(g_pos, e_pos) #normalized with e_pos (in J)

    #check to make sure DOS is normalized to 1. The printed value should equal 1 if the normalization
is correct.
    print(np.trapz(g_norm, e_pos))

    #handle division by zero
    epsilon = 1e-10 #constant to avoid dividing by 0
    Temp = np.array(Temp, dtype=float)
    Temp[Temp < epsilon] = epsilon

    Num_Density = N_atom/(V_cell * 1e-30) # number density in atom/(m^3)
    Vm = (Molec_Wt/1000)/(Density*1000)  # molar volume in m^3/mol

    #Compute Cv values:
    Cv_vib = [] #vibrational constant volume heat capcity defined
    for T in Temp:
```

```
        X = e_pos / (k * T)

        # Handle division by zero
        denominator = (np.exp(X) - 1)
        denominator[denominator < epsilon] = epsilon

        xx = (g_norm * (X**2) * np.exp(X)) / denominator**2 #stuff inside the integral
        integral_T = (k * 3 * Num_Density * np.trapz(xx, e_pos)) #compute the integral for every value
of T

        Cv_vib.append(integral_T)
    Cv_vib = np.array(Cv_vib)

    Cv_elec = ((pi**2) / 3) * (k**2) * g_Ef * Temp #electronic term for Cv
    Cv_total = Cv_vib + Cv_elec #total Cv from vib + elec

    #convert bulk and shear modulus values to Pa = (J/m^3)
    G_vrh_Pa = G_vrh*1e9
    B_vrh_Pa = B_vrh*1e9

    #Calculate thermal expansion coefficient and dilation terms
    Alpha =  (27 * Cv_total)/((20 * G_vrh_Pa) + (6 * B_vrh_Pa))
    Dilation = ((Alpha**2)*B_vrh_Pa*Temp)

    Cp = (Cv_total + Dilation)*Vm
    return Cp


def Plot(Temp, Cp, formula):
    #### plot Cp
    fig = plt.figure()
    ax = plt.subplot(1,1,1)
```

```
    plt.ylabel('$\mathrm{C_{p}}$ / J$\cdot$mol$^{-1}\cdot$K$^{-1}$')
    plt.xlabel('Temperature / K')

    #set range of axes
    xmin=0
    xmax=max(Temp)
    plt.xlim(xmin, xmax)
    ymin=0
    ymax=max(Cp)
    plt.ylim(ymin, ymax)

    #make square graph
    ax.set_aspect((xmax-xmin)/(ymax-ymin))
    #make five tick marks on each axis
    plt.gca().xaxis.set_major_locator(MaxNLocator(integer=True, nbins=6))
    plt.gca().yaxis.set_major_locator(MaxNLocator(integer=True, nbins=6))
    plt.plot(Temp, Cp, '-', color='darkred', linestyle='-', linewidth=3, label=f'{formula}')
    plt.legend()
    plt.savefig(f'Cp_vs_T_{formula}.png', dpi=800)
    plt.show()



def get_eDOS(material_id):
    API_KEY = "your API key here"
    # Initialize MPRester with your API key
    with MPRester(API_KEY) as mpr:

        dos = mpr.get_dos_by_material_id(material_id) #units= states / eV
        if dos is None:
            dos = np.nan
            dos.energies= np.nan #to avoid dividing by 0

        total_density = sum(dos.densities.values()) # Sum over both spins, if present, units = states / eV
```

```
    min_index = np.argmin(abs(dos.energies - dos.efermi))
    dos_energies = dos.energies * 1.602177e-19 #convert eV to J, units = J

    dos_integral = np.trapz(total_density, dos_energies)
    dos_norm = total_density/dos_integral # J^-1
    #check to make sure DOS is normalized to 1
    print('check if normalized (dos_norm/dos_energies:')
    print(np.trapz(dos_norm, dos_energies)) #units = J^-1 * J = unitless

    structure = mpr.get_structure_by_material_id(material_id)
    # Count the occurrences of each element in the unit cell
    element_counts = Counter(site.specie.symbol for site in structure)
    total_electrons = 0
    for element, count in element_counts.items():
        # Get the atomic number (total number of electrons) for the element
        atomic_number = Element(element).Z

        # Multiply the atomic number by the number of atoms in the unit cell
        total_electrons += atomic_number * count

    cell_volume = structure.volume #in cubic Angstrom
    charge_density = total_electrons/(cell_volume * 1e-30) #unit = states / m^3
    dos_norm_charge = charge_density * dos_norm # unit = states * J^-1 * m^-3
    eDOS_at_Fermi = dos_norm_charge[min_index] # unit = states * J^-1 * m^-3
    return eDOS_at_Fermi

##### Example usage
data_input = 'HeatCapacity_DataEntry_ForVDE.csv'
df = pd.read_csv(data_input, header=1)
for i, row in df.iterrows():
    # Initialize an empty list to store DataFrames for each material

    formula = df['Formula'][i]
    ID = df['Material_ID'][i]
```

```
N_atom = df['Num_Atoms'][i]
Density = df['Density_g_per_cubcm'][i]
V_cell = df['Vol_Cell_cubAngstrom'][i]
B_vrh = float(df['B_vrh_Gpa'][i])
G_vrh = float(df['G_vrh_Gpa'][i])
Molec_Wt = df['Molecular_Weight_g_per_mol'][i]

#For electronic density of states, either enter in value manually, use API, or enter '0'
#g_Ef = df['Elec_DOS_eF'][i]
#g_Ef = 0
g_Ef = get_eDOS(df['Material_ID'][i])



#get the DOS for that material
DOSdf = pd.read_csv(df['Formula'][i]+".csv", header=0)
e_raw_meV = DOSdf['meV']
g_raw = DOSdf['DOS']

Temps = np.arange(5, 600) #define temperatures that should be run

# Call the Cp_model function
Cp = Cp_model(e_raw_meV, g_raw, B_vrh, G_vrh, Density, Molec_Wt, N_atom, V_cell, g_Ef, Temps)

result_df = pd.DataFrame({
   'Temp':Temps,
   'Cp': Cp,
})

# Save the result_df DataFrame to a CSV file for the current material
result_filename = f'Cp_output_{formula}.csv'
result_df.to_csv(result_filename, index=False)

Plot(Temps, Cp, formula)
```

Supplemental Note 2:

Table S1. *Inputs used for the VDE model, obtained from the Materials Project Database.*[1]

Formula	*Material's Project ID*	*Number of atoms*	*Density* $(g \cdot cm^{-3})$	*Unit cell volume* (Å^3)	*Electronic DOS at Fermi level* $(states \cdot J^{-1} \cdot m^{-3})$	*VRH bulk modulus* (GPa)	*VRH shear modulus* (GPa)	*Molecular weight* $(g \cdot mol^{-1})$
Si	mp-149	8	2.31	161.32	0	89	62	28.085
KI	mp-22898	8	3.1	355.63	0	11	6	166.003
NaI	mp-23268	8	3.73	266.76	0	15	8	149.894
KBr	mp-23251	8	2.76	286.07	0	13	7	119.00
NaBr	mp-22916	8	3.29	207.8	0	19	11	102.89
KCl	mp-23193	8	2	248.12	0	16	9	74.55
NaCl	mp-22862	8	2.22	174.5	0	24	14	58.44
KF	mp-463	8	2.58	149.62	0	30	17	58.096
NaF	mp-682	8	2.92	95.56	0	46	30	41.988
CaO	mp-2605	8	3.35	111.08	0	106	74	56.08
MgO	mp-1265	8	3.63	73.77	0	151	119	40.305
TiC	mp-631	8	4.89	81.26	7.52E+46	251	172	63.91
CsCl	mp-22865	2	3.93	71.15	0	15	7	168.36
Fe	mp-13	2	7.9	23.47	4.54E+47	207	68	55.845
TiO2	mp-2657	6	4.24	62.61	0	209	107	79.866
CrN	mp-2132	8	5.96	73.61	2.90E+47	102	32	66.003
$MgAl_2O_3$	mp-3536	56	3.58	527.59	0	183	98	142.27
$AlLiO_2$	mp-8001	12	3.44	95.39	0	143	97	65.92
Al_2BeO_4	mp-3081	28	3.71	227.11	0	215	147	126.973
B_2Ti	mp-1145	3	4.51	25.6	9.63E+46	254	257	69.489
$CMgO_3$	mp-5348	30	3.02	277.82	0	103	56	84.3139
B_2Mg	mp-763	3	2.67	28.6	1.03E+47	147	114	45.93
Co	mp-102	4	9.03	43.37	1.22E+47	201	120	58.933
LiF	mp-1138	8	2.53	68.09	0	70	50	25.99
MoS_2	mp-1434	9	4.67	170.76	0	25	16	160.07
FeS_2*-pyrite*	mp-226	12	5.07	157.19	0	146	135	119.98
PbI_2	mp-567503	6	5.7	268.73	0	1	2	460.809
Li_2O_3Ti	mp-2931	48	3.46	420.99	0	110	72	109.76
Li_2O	mp-1960	12	1.97	100.79	0	79	68	29.9
Li_3N	mp-2251	4	1.33	43.5	0	52	34	34.83
SiO_2 *- quartz*	mp-6930	9	2.63	113.71	0	34	46	60.08
$ZnSO_4$	mp-5126	24	3.93	272.6	0	71	33	161.47
C_3Al_4	mp-1591	21	2.99	239.71	0	159	113	143.96
Ni	mp-23	4	9.29	41.97	1.60E+47	174	92	58.6934
Mg_2O_4Si	mp-2895	28	3.24	288.81	0	119	75	140.69
LiCl	mp-22905	8	2.14	131.43	0	32	21	42.47
Al_2O_5Si*-Andalusite*	mp-4753	32	3.16	340.97	0	143	89	162.043
Hf	mp-103	2	13.31	44.53	7.06E+47	108	57	178.49

Table S2. *Model outputs at 100K. Outputs for vibrational, electronic, dilation terms and total heat capacity from the VDE model are shown, as well as Dulong-Petit (DP) model outputs, Debye model outputs, and experimental[7] heat capacity values for temperature = 100K. All values are reported in* $J \cdot mol^{-1} \cdot K^{-1}$.

Formula	$VDE\ C_V{}^{vib}$	$VDE\ C_V{}^{elec}$	$VDE\ C_V{}^{total}$	VDE Dilation	$VDE\ C_p$	$DP\ C_p$	$Debye\ C_p$	$Exp.\ C_p$
Si	8.55	0	8.55	0.01	8.56	24.97	6.07	7.27
KI	42.18	0	42.18	0.77	42.95	49.89	45.92	45
NaI	40.17	0	40.17	0.7	40.87	49.92	44.67	43.03
KBr	41.73	0	41.73	0.81	42.53	49.94	44.08	43.15
NaBr	38.88	0	38.88	0.6	39.48	49.87	40.84	40.17
KCl	39.63	0	39.63	0.65	40.28	49.78	39.27	39.2
NaCl	35.15	0	35.15	0.46	35.61	49.99	33.36	34.93
KF	31.56	0	31.56	0.36	31.92	49.87	31.39	31.61
NaF	3.34	0	3.34	0	3.34	49.86	20.71	22.84
CaO	18.44	0	18.44	0.04	18.48	49.94	11.47	14.72
MgO	10.35	0	10.35	0.01	10.36	49.87	5.12	7.8
TiC	12.61	0.06	12.67	0.01	12.68	53.29	5.37	7.34
CsCl	40.9	0	40.9	0.81	41.71	49.88	45.62	44.09
Fe	21.34	0.2	21.54	0.15	21.68	24.95	11.39	12.1
TiO2	16.09	0	16.09	0.02	16.11	74.77	11.64	18.5
CrN	16.78	0.2	16.98	0.12	17.1	49.85	27.32	9.99
$MgAl_2O_3$	24.95	0	24.95	0.02	24.97	174.71	22.59	22.67
$AlLiO_2$	16.47	0	16.47	0.02	16.49	99.85	10.87	17.97
Al_2BeO_4	21.42	0	21.42	0.01	21.43	174.77	12.11	16.37
B_2Ti	16.85	0.09	16.94	0.01	16.95	74.79	3.31	7.52
$CMgO_3$	30.65	0	30.65	0.08	30.74	124.87	25.5	24.68
B_2Mg	16.2	0.11	16.31	0.02	16.33	74.74	5.48	11.17
Co	15.43	0.05	15.48	0.04	15.52	24.93	7.95	13.91
LiF	17.18	0	17.18	0.07	17.26	49.99	9.04	12.82
MoS_2	29.09	0	29.09	0.2	29.3	74.83	58.36	24.05
FeS_2-pyrite	30.53	0	30.53	0.03	30.57	74.83	13.57	18.71
PbI_2	65.92	0	65.92	1.85	67.77	74.76	73.71	69.79
Li_2O_3Ti	31.7	0	31.7	0.06	31.76	149.81	25.96	31.14
Li_2O	12.33	0	12.33	0.02	12.34	74.85	6.62	10.45
Li_3N	21.82	0	21.82	0.07	21.89	99.74	16.03	22.2
SiO_2 - quartz	13.84	0	13.84	0.02	13.86	74.89	21.17	15.69
$ZnSO_4$	47.06	0	47.06	0.24	47.3	149.83	69.14	47.59
C_3Al_4	33.44	0	33.44	0.03	33.47	174.71	17.8	29.7
Ni	15.86	0.06	15.92	0.06	15.98	24.94	9.91	13.63
Mg_2O_4Si	37.77	0	37.77	0.06	37.83	174.37	30.2	32.32
LiCl	30.15	0	30.15	0.29	30.44	50.03	24.7	36.31
Al_2O_5Si- Andalusite	32.61	0	32.61	0.03	32.64	199.33	27.86	30.32
Hf	21.92	0.59	22.51	0.09	22.61	24.95	18.86	20.58

Table S3. *Model outputs at 200K. Outputs for vibrational, electronic, dilation terms and total heat capacity from the VDE model are shown, as well as Dulong-Petit (DP) model outputs, Debye model outputs, and experimental[7] heat capacity values for temperature = 200K. All values are reported in* $J \cdot mol^{-1} \cdot K^{-1}$.

Formula	$VDE\ C_V{}^{vib}$	$VDE\ C_V{}^{elec}$	$VDE\ C_V{}^{total}$	VDE Dilation	$VDE\ C_p$	$DP\ C_p$	$Debye\ C_p$	$Exp.\ C_p$
Si	16.8	0	16.8	0.1	16.89	24.97	16	15.64
KI	46.4	0	46.4	1.86	48.26	49.89	48.86	50.52
NaI	46.12	0	46.12	1.85	47.97	49.92	48.53	49.85
KBr	46.8	0	46.8	2.03	48.83	49.94	48.38	49.87
NaBr	45.16	0	45.16	1.62	46.78	49.87	47.36	48.74
KCl	46.54	0	46.54	1.78	48.32	49.78	46.81	48.44
NaCl	45.1	0	45.1	1.5	46.6	49.99	44.87	46.87
KF	43.29	0	43.29	1.35	44.64	49.87	44.03	45.19
NaF	12.5	0	12.5	0.1	12.6	49.86	38.71	40.81
CaO	36.65	0	36.65	0.28	36.92	49.94	31.28	33.68
MgO	28.58	0	28.58	0.15	28.73	49.87	21.67	26.68
TiC	29.69	0.12	29.81	0.1	29.92	53.29	22.95	14.92
CsCl	46.28	0	46.28	2.07	48.35	49.88	48.76	50.13
Fe	23.71	0.4	24.11	0.37	24.48	24.95	19.97	21.59
TiO2	41.14	0	41.14	0.24	41.38	74.77	39.73	42.01
CrN	33.74	0.4	34.14	1	35.14	49.85	42.24	29.13
$MgAl_2O_3$	82.35	0	82.35	0.49	82.83	174.71	85.18	78.07
$AlLiO_2$	49.74	0	49.74	0.34	50.09	99.85	44.72	48
Al_2BeO_4	75.47	0	75.47	0.29	75.76	174.77	61.16	66.43
B_2Ti	37.7	0.19	37.88	0.08	37.96	74.79	19.71	28.13
$CMgO_3$	66.89	0	66.89	0.8	67.69	124.87	74.59	57.57
B_2Mg	36.83	0.22	37.05	0.17	37.22	74.74	27.02	34.39
Co	21.62	0.1	21.72	0.16	21.88	24.93	17.68	22.23
LiF	35.31	0	35.31	0.61	35.93	49.99	28.37	32.8
MoS_2	54.88	0	54.88	1.45	56.33	74.83	70.14	51.84
FeS_2-pyrite	53.45	0	53.45	0.2	53.65	74.83	42.52	49.06
PbI_2	70.03	0	70.03	4.18	74.21	74.76	74.5	75.4
Li_2O_3Ti	83.45	0	83.45	0.8	84.25	149.81	83.47	82.66
Li_2O	38.67	0	38.67	0.34	39.01	74.85	30.05	37.2
Li_3N	56.9	0	56.9	0.95	57.85	99.74	53.77	55.42
SiO_2 - quartz	36.33	0	36.33	0.23	36.56	74.89	50.81	32.64
$ZnSO_4$	86.9	0	86.9	1.61	88.51	149.83	120.36	78.05
C_3Al_4	89.55	0	89.55	0.37	89.92	174.71	75.63	82.1
Ni	21.41	0.13	21.53	0.22	21.76	24.94	19.08	22.47
Mg_2O_4Si	91.65	0	91.65	0.68	92.34	174.37	97.14	86.14
LiCl	42.65	0	42.65	1.14	43.79	50.03	41.06	43.35
Al_2O_5Si- Andalusite	94.65	0	94.65	0.52	95.18	199.33	100.81	84.61
Hf	23.76	1.19	24.95	0.23	25.18	24.95	23.19	24.42

Table S4. *Model outputs at 300K. Outputs for vibrational, electronic, dilation terms and total heat capacity from the VDE model are shown, as well as Dulong-Petit (DP) model outputs, Debye model outputs, and experimental[7] heat capacity values for temperature = 300K. All values are reported in* $J \cdot mol^{-1} \cdot K^{-1}$.

Formula	$VDE\ C_V{}^{vib}$	$VDE\ C_V{}^{elec}$	$VDE\ C_V{}^{total}$	VDE Dilation	$VDE\ C_p$	$DP\ C_p$	$Debye\ C_p$	$Exp.\ C_p$
Si	20.55	0	20.55	0.21	20.76	24.97	20.27	20.05
KI	47.58	0	47.58	2.94	50.52	49.89	49.43	52.81
NaI	47.71	0	47.71	2.97	50.69	49.92	49.29	52.26
KBr	48.16	0	48.16	3.22	51.38	49.94	49.24	52.3
NaBr	47.02	0	47.02	2.63	49.65	49.87	48.73	51.44
KCl	48.14	0	48.14	2.86	50.99	49.78	48.43	51.33
NaCl	47.6	0	47.6	2.51	50.11	49.99	47.62	50.54
KF	46.53	0	46.53	2.33	48.87	49.87	47.14	49.02
NaF	23.1	0	23.1	0.49	23.59	49.86	44.41	46.92
CaO	43.06	0	43.06	0.57	43.63	49.94	40.09	42.24
MgO	37.92	0	37.92	0.4	38.32	49.87	33.16	37.24
TiC	39.39	0.18	39.58	0.27	39.85	53.29	35.26	33.94
CsCl	47.82	0	47.82	3.31	51.12	49.88	49.38	52.47
Fe	24.3	0.6	24.9	0.59	25.49	24.95	22.54	25.14
TiO2	55.18	0	55.18	0.64	55.82	74.77	55.25	55.29
CrN	40.92	0.6	41.52	2.22	43.73	49.85	46.25	51.39
$MgAl_2O_3$	119.37	0	119.37	1.53	120.91	174.71	123.46	116.53
$AlLiO_2$	70.22	0	70.22	1.03	71.25	99.85	67.46	68.13
Al_2BeO_4	113.14	0	113.14	0.98	114.12	174.77	103.16	105.94
B_2Ti	51.33	0.28	51.61	0.22	51.83	74.79	37.45	44.52
$CMgO_3$	88.32	0	88.32	2.08	90.4	124.87	97.91	76.5
B_2Mg	50.51	0.33	50.85	0.48	51.33	74.74	44.93	47.87
Co	23.26	0.15	23.41	0.28	23.69	24.93	21.26	23.98
LiF	42.36	0	42.36	1.33	43.68	49.99	38.2	41.94
MoS_2	64.47	0	64.47	3	67.47	74.83	72.69	63.68
FeS_2-pyrite	62.95	0	62.95	0.42	63.36	74.83	57.22	62.29
PbI_2	71.32	0	71.32	6.5	77.82	74.76	74.65	77.61
Li_2O_3Ti	111.93	0	111.93	2.15	114.08	149.81	113.41	110.75
Li_2O	53.94	0	53.94	0.98	54.93	74.85	47.68	54.38
Li_3N	75.38	0	75.38	2.51	77.89	99.74	74.25	75.54
SiO_2 - quartz	50.65	0	50.65	0.66	51.31	74.89	62.53	44.77
$ZnSO_4$	110.19	0	110.19	3.89	114.09	149.83	135.59	99.37
C_3Al_4	123.81	0	123.81	1.07	124.88	174.71	115.92	117.3
Ni	23.09	0.19	23.28	0.39	23.67	24.94	22.06	26.02
Mg_2O_4Si	123.33	0	123.33	1.86	125.19	174.37	131.99	119.15
LiCl	46.27	0	46.27	2.02	48.28	50.03	45.73	48.1
Al_2O_5Si- Andalusite	134.92	0	134.92	1.6	136.51	199.33	143.58	123.32
Hf	24.28	1.78	26.06	0.37	26.43	24.95	24.14	25.71

Table S5. *Model outputs at 400K. Outputs for vibrational, electronic, dilation terms and total heat capacity from the VDE model are shown, as well as Dulong-Petit (DP) model outputs, Debye model outputs, and experimental*[7] *heat capacity values for temperature = 400K. All values are reported in* $J \cdot mol^{-1} \cdot K^{-1}$.

Formula	$VDE\ C_V{}^{vib}$	$VDE\ C_V{}^{elec}$	$VDE\ C_V{}^{total}$	VDE Dilation	VDE C_p	DP C_p	Debye C_p	Exp. C_p
Si	22.28	0	22.28	0.34	22.62	24.97	22.16	22.14
KI	48.11	0	48.11	4.01	52.12	49.89	49.63	53.93
NaI	48.39	0	48.39	4.08	52.46	49.92	49.57	53.81
KBr	48.74	0	48.74	4.39	53.14	49.94	49.55	53.81
NaBr	47.93	0	47.93	3.65	51.57	49.87	49.23	53.45
KCl	48.74	0	48.74	3.9	52.65	49.78	49.01	53.14
NaCl	48.58	0	48.58	3.49	52.07	49.99	48.64	52.35
KF	47.85	0	47.85	3.29	51.14	49.87	48.31	51.05
NaF	30.98	0	30.98	1.17	32.14	49.86	46.68	49.6
CaO	45.8	0	45.8	0.87	46.67	49.94	44.02	46.63
MgO	42.46	0	42.46	0.66	43.12	49.87	39.3	42.56
TiC	44.48	0.25	44.73	0.46	45.19	53.29	41.87	40.69
CsCl	48.5	0	48.5	4.54	53.04	49.88	49.6	54.69
Fe	24.54	0.81	25.34	0.81	26.15	24.95	23.55	27.39
TiO2	62.39	0	62.39	1.09	63.49	74.77	62.76	62.84
CrN	44.25	0.81	45.05	3.48	48.53	49.85	47.78	49.08
$MgAl_2O_3$	139.32	0	139.32	2.79	142.11	174.71	142.81	137.99
$AlLiO_2$	81.04	0	81.04	1.83	82.87	99.85	79.44	81.55
Al_2BeO_4	134.57	0	134.57	1.85	136.43	174.77	128.04	130.28
B_2Ti	59.29	0.37	59.66	0.39	60.04	74.79	49.42	54.89
$CMgO_3$	100.64	0	100.64	3.61	104.24	124.87	108.57	89.86
B_2Mg	58.61	0.44	59.05	0.87	59.92	74.74	55.38	54.39
Co	23.91	0.2	24.11	0.4	24.51	24.93	22.77	24.83
LiF	45.4	0	45.4	2.03	47.43	49.99	42.81	46.54
MoS_2	68.58	0	68.58	4.53	73.11	74.83	73.62	68.91
FeS_2-pyrite	67.38	0	67.38	0.64	68.02	74.83	64.1	68.85
PbI_2	71.93	0	71.93	8.82	80.75	74.76	74.7	78.86
Li_2O_3Ti	126.16	0	126.16	3.65	129.81	149.81	127.58	127.36
Li_2O	61.73	0	61.73	1.72	63.45	74.85	57.46	64
Li_3N	84.54	0	84.54	4.2	88.74	99.74	84.1	87.1
SiO_2 - quartz	58.88	0	58.88	1.19	60.07	74.89	67.55	53.43
$ZnSO_4$	123.53	0	123.53	6.52	130.05	149.83	141.57	116.02
C_3Al_4	142.12	0	142.12	1.88	144	174.71	137.49	138.51
Ni	23.81	0.25	24.07	0.56	24.62	24.94	23.26	28.49
Mg_2O_4Si	140.93	0	140.93	3.24	144.17	174.37	148.49	137.56
LiCl	47.77	0	47.77	2.86	50.63	50.03	47.55	50.97
Al_2O_5Si- Andalusite	157.44	0	157.44	2.9	160.33	199.33	164.81	149.57
Hf	24.51	2.37	26.88	0.53	27.42	24.95	24.49	26.75

Table S6. *Model outputs at 500K. Outputs for vibrational, electronic, dilation terms and total heat capacity from the VDE model are shown, as well as Dulong-Petit (DP) model outputs, Debye model outputs, and experimental[7] heat capacity values for temperature = 500K. All values are reported in* $J \cdot mol^{-1} \cdot K^{-1}$.

Formula	$VDE\ C_V{}^{vib}$	$VDE\ C_V{}^{elec}$	$VDE\ C_V{}^{total}$	VDE Dilation	$VDE\ C_p$	$DP\ C_p$	$Debye\ C_p$	$Exp.\ C_p$
Si	23.18	0	23.18	0.46	23.64	24.97	23.12	23.33
KI	48.4	0	48.4	5.07	53.47	49.89	49.73	55.36
NaI	48.73	0	48.73	5.17	53.9	49.92	49.69	55.02
KBr	49.04	0	49.04	5.56	54.6	49.94	49.69	55.25
NaBr	48.45	0	48.45	4.66	53.11	49.87	49.46	54.85
KCl	49.03	0	49.03	4.94	53.97	49.78	49.29	54.75
NaCl	49.06	0	49.06	4.45	53.51	49.99	49.12	53.94
KF	48.51	0	48.51	4.23	52.74	49.87	48.86	52.72
NaF	36.21	0	36.21	1.99	38.2	49.86	47.79	51.26
CaO	47.2	0	47.2	1.15	48.35	49.94	46.03	48.98
MgO	44.88	0	44.88	0.92	45.8	49.87	42.7	45.54
TiC	47.3	0.31	47.61	0.65	48.26	53.29	45.54	45.17
CsCl	48.87	0	48.87	5.76	54.63	49.88	49.7	56.9
Fe	24.66	1.01	25.67	1.04	26.7	24.95	24.04	29.7
TiO2	66.36	0	66.36	1.55	67.91	74.77	66.74	67.2
CrN	46	1.01	47.01	4.73	51.74	49.85	48.51	49.75
$MgAl_2O_3$	150.51	0	150.51	4.07	154.58	174.71	153.26	149.83
$AlLiO_2$	87.05	0	87.05	2.63	89.68	99.85	86.04	88.41
Al_2BeO_4	146.99	0	146.99	2.77	149.76	174.77	142.54	145.25
B_2Ti	63.98	0.47	64.45	0.56	65.01	74.79	56.89	61.67
$CMgO_3$	107.93	0	107.93	5.19	113.12	124.87	114.07	100.06
B_2Mg	63.44	0.56	64	1.28	65.27	74.74	61.42	58.41
Co	24.23	0.25	24.48	0.52	24.99	24.93	23.51	26.53
LiF	46.94	0	46.94	2.71	49.66	49.99	45.21	49.33
MoS_2	70.67	0	70.67	6.01	76.68	74.83	74.05	71.76
FeS_2-pyrite	69.73	0	69.73	0.86	70.58	74.83	67.69	72.04
PbI_2	72.28	0	72.28	11.13	83.41	74.76	74.72	80.39
Li_2O_3Ti	133.85	0	133.85	5.13	138.99	149.81	135.01	135.65
Li_2O	65.99	0	65.99	2.46	68.44	74.85	62.98	69.58
Li_3N	89.48	0	89.48	5.89	95.36	99.74	89.3	96.94
SiO_2 - quartz	63.73	0	63.73	1.74	65.48	74.89	70.07	59.64
$ZnSO_4$	131.42	0	131.42	9.22	140.64	149.83	144.47	131.21
C_3Al_4	152.35	0	152.35	2.7	155.06	174.71	149.45	150.89
Ni	24.19	0.32	24.5	0.72	25.23	24.94	23.84	31.05
Mg_2O_4Si	151.16	0	151.16	4.66	155.82	174.37	157.13	148.7
LiCl	48.53	0	48.53	3.7	52.22	50.03	48.42	53.34
Al_2O_5Si- Andalusite	170.42	0	170.42	4.24	174.66	199.33	176.18	165.37
Hf	24.63	2.97	27.6	0.7	28.3	24.95	24.65	27.69

Table S7. *Model outputs at 600K. Outputs for vibrational, electronic, dilation terms and total heat capacity from the VDE model are shown, as well as Dulong-Petit (DP) model outputs, Debye model outputs, and experimental*[7] *heat capacity values for temperature = 600K. All values are reported in* $J \cdot mol^{-1} \cdot K^{-1}$.

Formula	$VDE\ C_V{}^{vib}$	$VDE\ C_V{}^{elec}$	$VDE\ C_V{}^{total}$	VDE Dilation	$VDE\ C_p$	$DP\ C_p$	$Debye\ C_p$	$Exp.\ C_p$
Si	23.7	0	23.7	0.57	24.27	24.97	23.66	24.15
KI	48.57	0	48.57	6.13	54.69	49.89	49.78	57.3
NaI	48.93	0	48.93	6.25	55.18	49.92	49.76	56.23
KBr	49.22	0	49.22	6.72	55.94	49.94	49.77	56.36
NaBr	48.77	0	48.77	5.67	54.44	49.87	49.58	56.07
KCl	49.19	0	49.19	5.96	55.16	49.78	49.44	56.32
NaCl	49.34	0	49.34	5.4	54.74	49.99	49.38	55.48
KF	48.89	0	48.89	5.15	54.04	49.87	49.17	54.27
NaF	39.67	0	39.67	2.87	42.54	49.86	48.41	52.68
CaO	48	0	48	1.43	49.42	49.94	47.18	50.48
MgO	46.3	0	46.3	1.18	47.48	49.87	44.73	47.43
TiC	48.99	0.37	49.36	0.84	50.19	53.29	47.73	47.65
CsCl	49.09	0	49.09	6.98	56.06	49.88	49.75	59.1
Fe	24.73	1.21	25.94	1.27	27.21	24.95	24.32	32.05
TiO2	68.73	0	68.73	1.99	70.72	74.77	69.06	69.93
CrN	47.03	1.21	48.23	5.98	54.21	49.85	48.92	50.42
$MgAl_2O_3$	157.23	0	157.23	5.33	162.56	174.71	159.4	157.89
$AlLiO_2$	90.65	0	90.65	3.43	94.07	99.85	89.95	92.69
Al_2BeO_4	154.61	0	154.61	3.67	158.28	174.77	151.42	154.97
B_2Ti	66.89	0.56	67.45	0.74	68.19	74.79	61.64	66.17
$CMgO_3$	112.47	0	112.47	6.76	119.23	124.87	117.22	109.04
B_2Mg	66.46	0.67	67.13	1.68	68.81	74.74	65.1	61.38
Co	24.41	0.3	24.71	0.63	25.34	24.93	23.94	28.2
LiF	47.82	0	47.82	3.38	51.2	49.99	46.6	51.59
MoS_2	71.85	0	71.85	7.46	79.31	74.83	74.29	73.6
FeS_2-pyrite	71.1	0	71.1	1.07	72.17	74.83	69.77	74.31
PbI_2	72.48	0	72.48	13.43	85.92	74.76	74.74	83.71
Li_2O_3Ti	138.39	0	138.39	6.59	144.97	149.81	139.3	141.4
Li_2O	68.5	0	68.5	3.18	71.68	74.85	66.3	73.82
Li_3N	92.38	0	92.38	7.53	99.92	99.74	92.32	106.43
SiO_2 - quartz	66.74	0	66.74	2.3	69.04	74.89	71.49	64.42
$ZnSO_4$	136.33	0	136.33	11.91	148.24	149.83	146.08	137.44
C_3Al_4	158.49	0	158.49	3.51	162.01	174.71	156.58	159.2
Ni	24.4	0.38	24.78	0.89	25.67	24.94	24.17	34.85
Mg_2O_4Si	157.47	0	157.47	6.06	163.54	174.37	162.13	156.43
LiCl	48.96	0	48.96	4.51	53.48	50.03	48.91	55.59
Al_2O_5Si- Andalusite	178.36	0	178.36	5.58	183.93	199.33	182.83	174.52
Hf	24.71	3.56	28.27	0.88	29.15	24.95	24.74	28.58

Heat capacity estimations and their comparisons to experimental values for the 38 materials (input parameters shown in Table S1) using the Dulong-Petit, Debye, and VDE models at 100, 200, 300, 400, 500, and 600 K are shown in Figures S1 and S2. Model outputs at higher temperatures generally had better agreement with experimental values. Across all temperatures, the VDE model had lower mean percent error values than the Debye and Dulong-petit models.

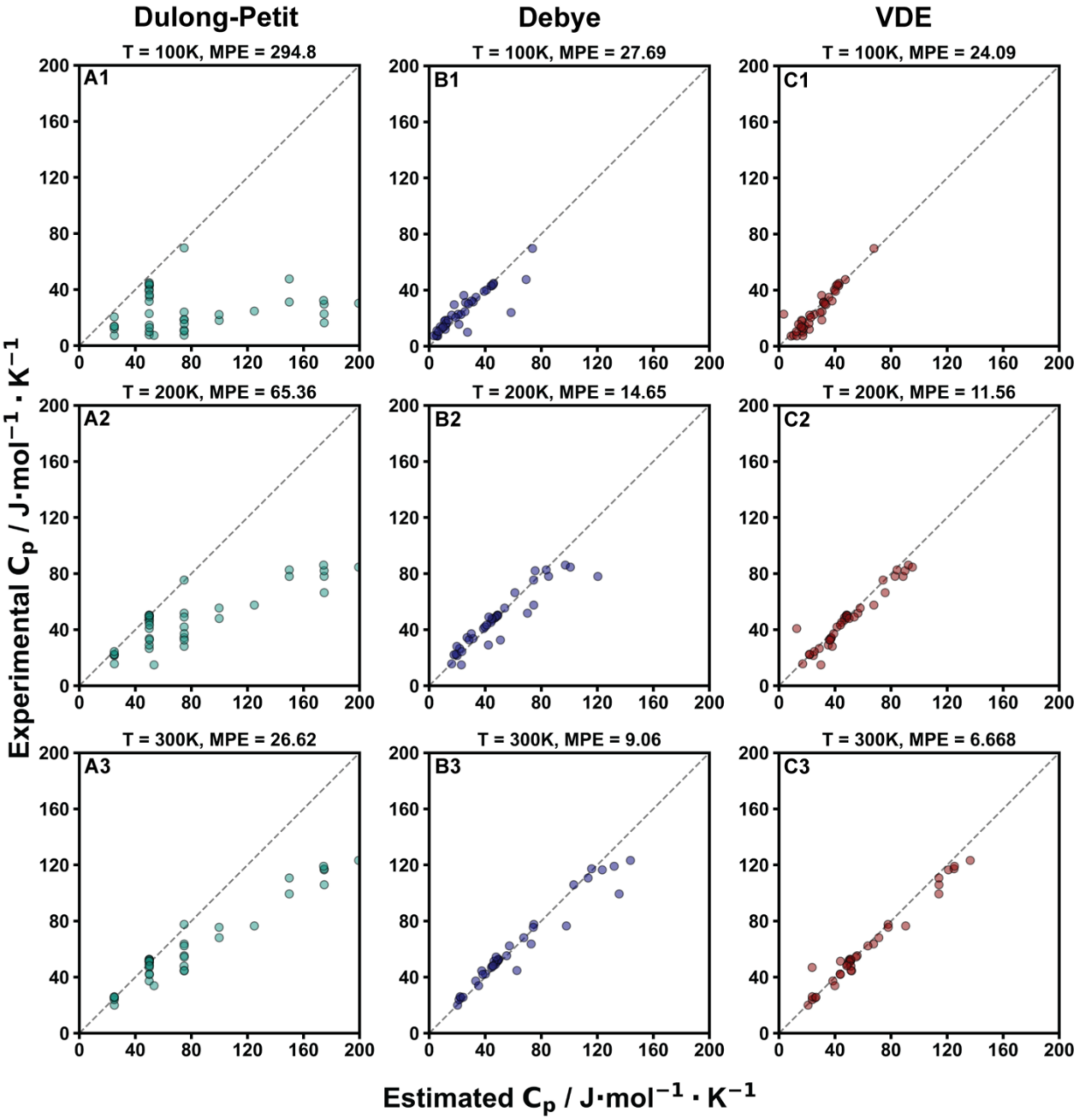

Figure S1. *Comparison of heat capacity estimates with experimental values for 38 diverse materials at* ***(1)*** *100 K,* ***(2)*** *200K, and* ***(3)*** *300K.* ***(a)*** *The Dulong-Petit estimate is found to significantly overestimate heat capacity values with MPE ranging from 26.62% to 249.8%.* ***(b)*** *The Debye model estimate is found to be significantly better than the Dulong-Petit values with MPE from 9.1% to 27.7%. However,* ***(c)*** *the VDE model estimate is found to be the most accurate, on average, with MPE values from 6.7% to 24.1%.*

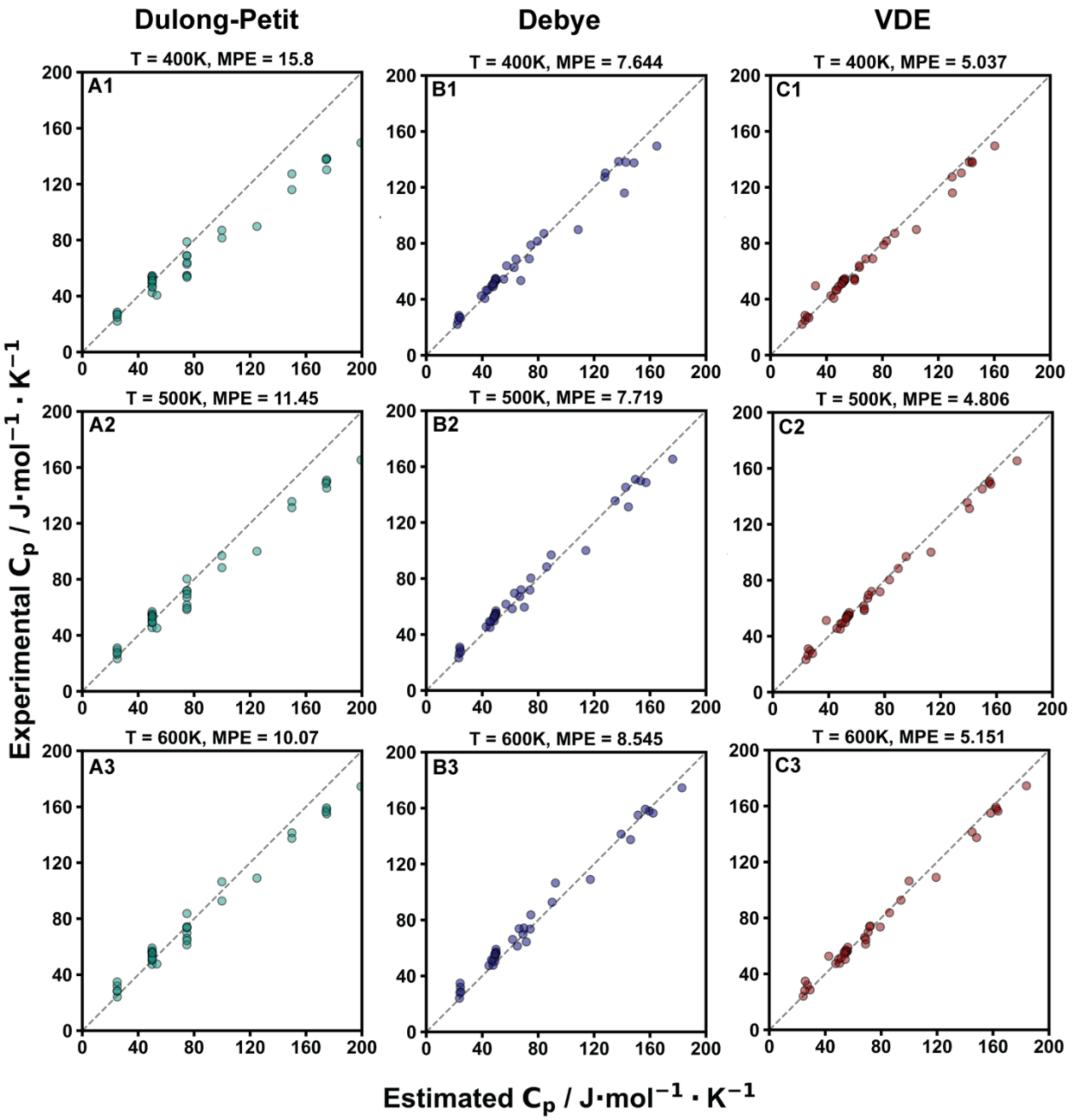

Figure S2. *Comparison of heat capacity estimates with experimental values for 38 diverse materials at* ***(1)*** *400 K,* ***(2)*** *500K, and* ***(3)*** *600K.* ***(a)*** *The Dulong-Petit estimate is found to significantly overestimate heat capacity values with MPE ranging from 10.1% to 15.8%.* ***(b)*** *The Debye model estimate is found to be significantly better than the Dulong-Petit values with MPE from 7.6% to 8.5%. However,* ***(c)*** *the VDE model estimate is found to be the most accurate, on average, with MPE values between 4.8% to 5.1%.*

Mean percent error estimations as a function of temperature are shown in Figure S3, with the whole range of the error in the Dulong-Petit estimation. The Dulong-Petit model has mean percent error values approaching almost 300% at 100 K, illustrating the model's drastic inaccuracy at lower temperatures due to its constant approximation of the phonon density of states.

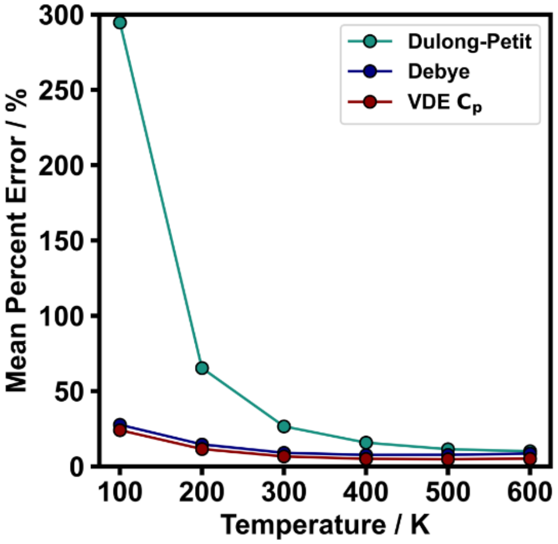

Figure S3. *Temperature dependence of mean percent error with the whole range of Dulong-Petit mean percent error shown. The same analysis of experimental constant-pressure heat capacity values and their corresponding estimated heat capacity values (as shown in Figure S1-S2) was conducted as a function of temperature for the Dulong-Petit (green), Debye (blue) and the VDE (red) models.*

Mean percent error estimations as a function of temperature are shown in Figure S4, with both the error from the VDE model's total C_{V} estimation (e.g. vibrational + electronic terms) and its C_{p} estimation (vibrational + electronic + dilation terms). The mean percent error of $C_{\mathrm{p}}$is lower than that of C_{V}, which converges to the Dulong-Petit estimation at higher temperatures. This illustrates the importance of the dilation term at higher temperatures for accurate heat capacity estimations.

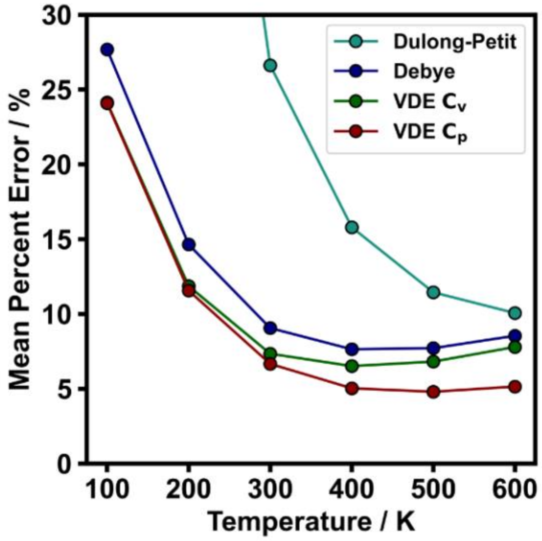

Figure S4. *Temperature dependence of mean percent error with the constant-volume heat capacity also shown. The same analysis of experimental constant-pressure heat capacity values and their corresponding estimated heat capacity values (as shown in Figure S1-S2) was conducted as a function of temperature for the Dulong-Petit (green), Debye (blue) and the VDE (red) models. The constant-volume heat capacity has higher mean percent-error at high temperatures than the constant-pressure heat capacity.*

Supplemental Note 3:

Structural optimizations and phonon computations were performed with DFT as implemented in VASP with plane wave basis sets.[8–11] For the electronic optimization, strict convergence energy and forces criteria EDIFF = $1E^{-7}$ and EDIFFG= -0.001 or $1E^{-5}$ per cell, respectively were used. The projector augmented-wave method with a plane wave cutoff between 520 eV and 700 eV and the Perdew-Burke-Ernzerhof (PBE) functional was used.[12–14]

Structural optimization was started from the structures provided (mp-id) in the Materials Project Database[1] (Table S8) using all allowed degrees of freedom. To ensure the same k-point mesh in the structural optimizations, a Γ-centered grid with a maximum separation of $0.12Å^{-1}$ was employed. Harmonic vibrational frequencies and normalized phonon eigenvectors were computed using the finite displacement method as implemented in PHONOPY[4,5,15] imposing individual atomic displacements of 0.01 Å. To ensure free imaginary modes dispersion curves, supercells greater than 17 Å per lattice was used (supercell lattice convergence was tested to ensure the reliability of our Grüneisen calculations). For most of the cases, the static runs were done at gamma point. Due to challenges with convergence and/or imaginary modes in phonon dispersion curves with some materials run through the VDE model, only 25 of the 38 materials used in the VDE analysis were investigated using DFT (Table S8).

For the Grüneisen parameter calculations, two additional relaxations were also performed under fixed-volume conditions: $0.997^3 \times V_0$ (contracted volume) and $1.003^3 \times V_0$ (expanded volume), where V_0 corresponds to the ground-state volume. Subsequently, harmonic phonon calculations were conducted following the same setup of the ground-state calculations. To obtain the mode-specific Grüneisen parameter and average Grüneisen parameter, PHONOPY and classes from pymatgen were used.

Furthermore, the non-analytical correction by Gonze et al., as implemented in PHONOPY , was applied to Si (mp-7000) and LiF (mp-1138) to ensure free imaginary modes dispersion curves.[16] For the remaining structures, the effect of this correction on the Grüneisen parameter was found to be negligible.

The thermal expansion coefficient α was calculated using the average of the N mode Grüneisen parameters γ_i as

$$\gamma = \frac{1}{N}\sum\gamma_i$$

in the relation

$$\alpha = \frac{C_V\gamma}{B},$$

where C_V is the sum of the vibrational and electronic terms obtained from the VDE model with the DFT-generated phonon density of states as an input, and C_p uses the above thermal expansion coefficient in the dilation term. Heat capacity estimations and their comparisons to experimental values (the same values as compared to previously with the other models in Supplemental Note 2) for the 25 materials (input parameters shown in Tables S1 and S8) using DFT are shown in Figures S5.

***Table S8**. Average Grüneisen parameter for the set of materials. For those structures, harmonic phonons were computed at gamma point.*

Formula	Material's Project ID	Average Grüneisen parameter
LiF	mp-1138	1.81
TiB_2	mp-1145	1.35
MgO	mp-1265	1.48
Si	mp-149	1.07
Al_4C_3	mp-1591	1.31
Li2O	mp-1960	1.42
FeS_2	mp-226	1.54
NaCl	mp-22862	2.31
KI	mp-22898	2.15
LiCl	mp-22905	2.86
NaBr	mp-22916	2.23
KCl	mp-23193	1.82
KBr	mp-23251	1.93
NaI	mp-23268	2.63
CaO	mp-2605	1.72
Mg_2SiO_4	mp-2895	1.34
$MgAl_2O_4$	mp-3536	1.28
KF	mp-463	2.47
Al_2SiO_5	mp-4753	1.66
TiC	mp-631	1.70
NaF	mp-682	1.93
SiO_2	mp-7000	1.06
MgB_2	mp-763	1.50
$LiAlO_2$	mp-8001	1.65
CsCl	mp-22865	2.84

*SiO_2 mp-7000 replaced mp-6930 from previous analyses

To access the full dataset of DFT-computed force constants, vibrational density of states, and Grüneisen parameters, please visit https://doi.org/10.5281/zenodo.15057994.

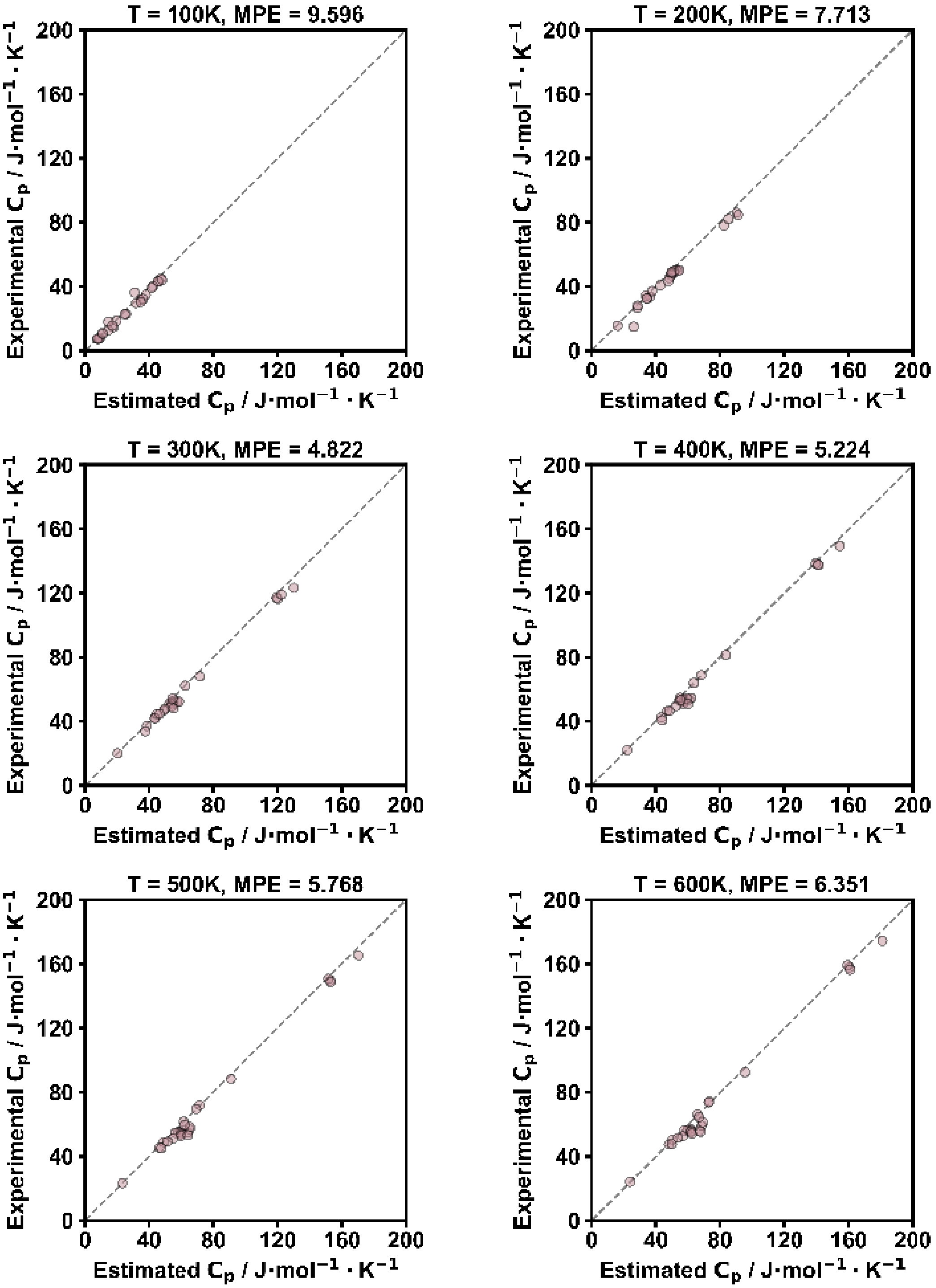

Figure S5. Comparison of DFT heat capacity estimates with experimental values for 25 diverse materials at 100 - 600K. Mean-percent errors (MPE) are shown along with the temperature at the top of each graph.

To compare the impact of the DFT-generated dilation term to the VDE model's dilation term, and to see the effectiveness of the DFT-generated phonon density of states in the VDE model, estimations of C_p were also generated using the DFT phonon density of states estimations, but the VDE model's dilation and electronic terms. Previously, C_v (vibrational and electronic components) were run through the VDE model, but the DFT-generated Grüneisen parameters were used for the thermal expansion coefficient in the dilation component instead of the estimation from phonon pressure theory (see Eq. 9 in the main text). A comparison of the mean percent error of the DFT heat capacity estimations can be seen in Figure S6, where no dilation is used (C_v alone), DFT dilation is used (with Grüneisen methodology described above), and with VDE dilation (from phonon pressure theory). All mean percent errors are relative to the same experimental dataset as described in the main text.

Upon inspection of Figure S6, one can see that the VDE dilation term reduces the mean percent error significantly from the estimate without (DFT-C_v) at high temperatures, but the DFT-determined dilation actually increases the mean percent error of the estimation at high temperatures. This indicates that the VDE model's dilation from phonon pressure theory brings the heat capacity estimations closer to experimental heat capacity values at higher temperatures, whereas the DFT-determined dilation has the opposite effect.

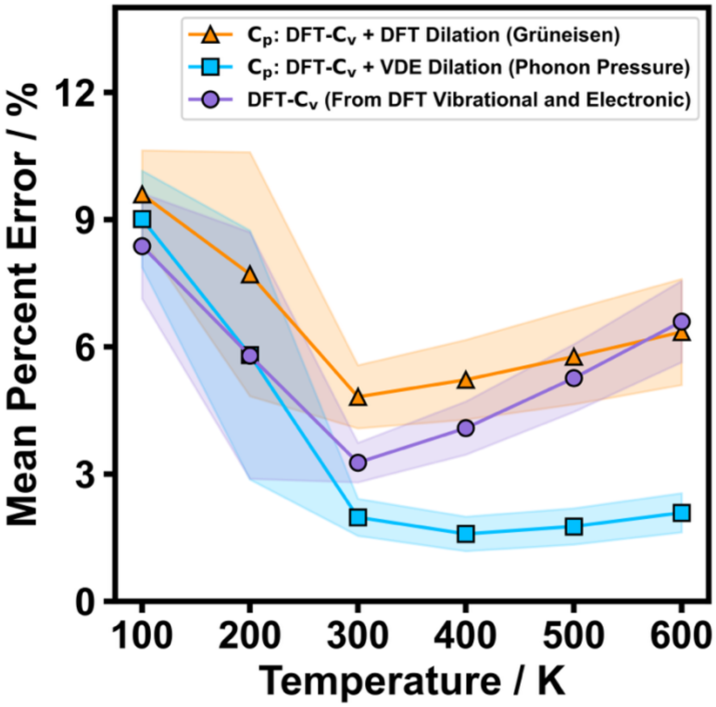

Figure S6. *Temperature dependence of mean percent error with the constant-volume heat capacity also shown. The same type of analysis of experimental constant-pressure heat capacity values and their corresponding estimated heat capacity values (as shown in Figure S3-S4) was conducted as a function of temperature for the DFT* C_v *values (purple circles), DFT* C_p *with a DFT-determined dilation term (orange triangles), and DFT* C_p *with the VDE dilation term (blue squares).*

Supplemental Note 4:

Table S9. *Inputs used for the VDE model for example materials, obtained from the Materials Project Database,*[1] *except for ZIF-8,*[17–20] *a-Si,*[21–24] *PVC,*[25–27] *and the elastic properties of Al_2O_3.*[28]

Formula	*Material's Project ID*	*Number of atoms*	*Density ($g \cdot cm^{-3}$)*	*Unit cell volume ($Å^3$)*	*Electronic DOS at Fermi level (states $\cdot J^{-1} \cdot m^{-3}$)*	*VRH bulk modulus (GPa)*	*VRH shear modulus (GPa)*	*Molecular weight ($g \cdot mol^{-1}$)*	*Thermal expansion coefficient (K^{-1})*
Mg_3Sb_2	mp-2646	5	4.0	131.2	0	42	18	316.44	calculated
Si	mp-149	8	2.3	161.3	0	89	62	28.09	calculated
$LiCoO_2$	mp-22526	12	5.1	95.2	0	135	69.9	97.87	calculated
$Zn(MeIm)_2$ -SOD (ZIF-8)		102	1.0	2474.7	0	6.52	Not used	229.60	180×10^{-6}
Cu_2Se	mp-16366	12	7.0	195.3	0	84	6	202.06	calculated
a-Si		512	2.2	10601.6	0	75	35	28.09	calculated
PVC		24	1.5	250.5	0	4.7	Not used	62.50	31.5×10^{-6}
Al_2O_3	mp-1143	30	3.9	262.3	0	254	163	101.96	calculated

Supplemental Note 5:

A comparison of the VDE model predictions of C_V (e.g. vibrational + electronic terms) and its C_p (vibrational + electronic + dilation terms) for Mg_3Sb_2 is shown in Figure S7. The C_V values converge to the Dulong-Petit estimation, whereas inclusion of the dilation term in the C_p estimation brings the VDE model's prediction significantly closer to experimental heat capacity values.

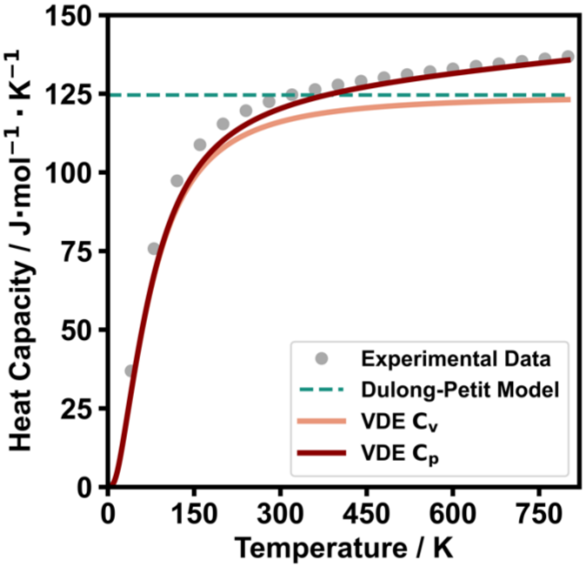

Figure S7. *Temperature dependent heat capacity of Mg_3Sb_2 with both constant volume (orange) and constant pressure (red) outputs from the VDE model. Estimations of Mg_3Sb_2 are compared to the Dulong-Petit model (teal dotted line) and to experimental values (gray points).*

Supplemental Note 6:

The phonon density of states output from the ALIGNN and MACE-MP-0-3b models are shown for ZIF-8 (Figure S8) and PVC (Figure S9). Density of states values for ZIF-8 are also compared to that reported by Hegde et al.[29] in Figure S8. Each trace was normalized such that the vibrational density of states below 120 meV integrates to 1. In ZIF-8, the full experimental phonon density of states integrates to 2.2 and the density of states for MACE-MP-0-3b integrates to 1.8 in comparison. Thus, only approximately ~45% of the vibrational modes in ZIF-8 fall within the range of energies used in the ALIGNN[30] training. Similarly in PVC, the density of states for MACE-MP-0-3b integrates to 2.2 in comparison.

In ZIF-8, an experimental value[19] for the thermal expansion coefficient of 180e-6 K^{-1} was used, as ZIF-8 is unavailable on materials project. Similarly in the case of PVC, an experimental value[24] for the thermal expansion coefficient of 31.5e-6 K^{-1} was used. As the vibrations of the hydrogen atoms are very localized, Gaussian smearing was used instead of the tetrahedron smearing method when computing the phonon densities of states with the MACE model.

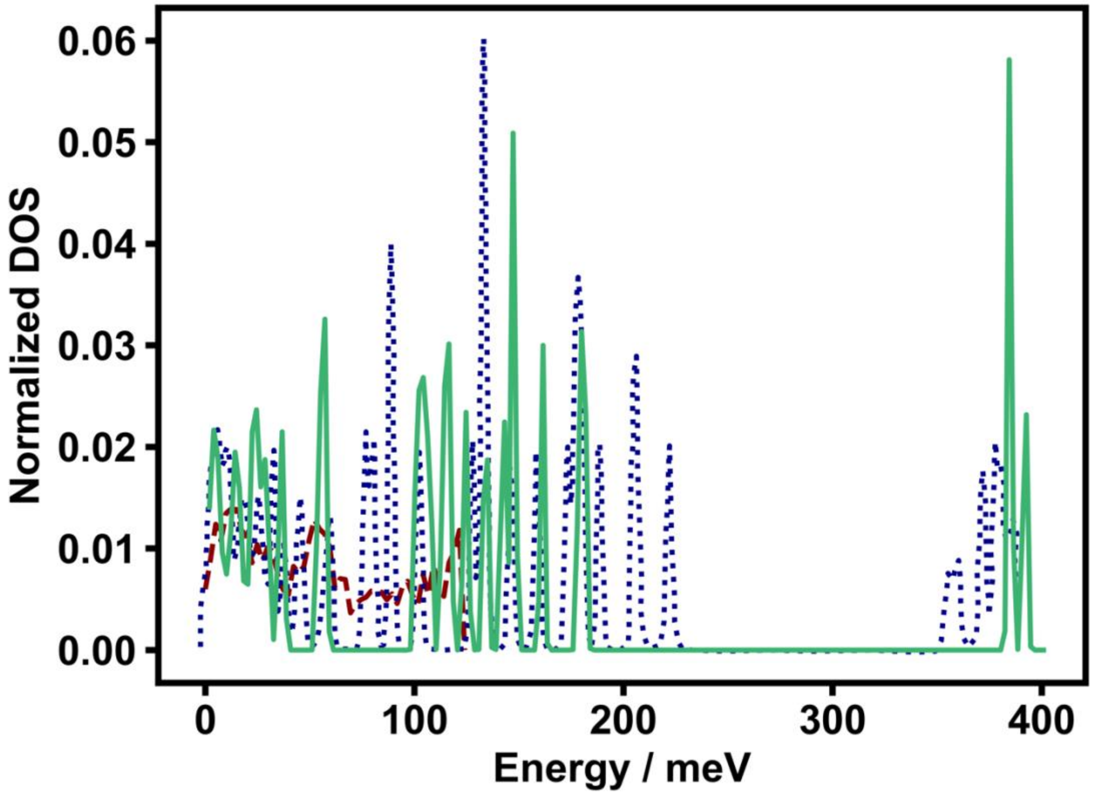

Figure S8. *Phonon density of states of ZIF-8 with the ALIGNN output (red dashed), the* MACE-MP-0-3b (green solid) *and that reported by Hegde et al.,*[29] *(blue dotted) normalized to the same scale, such that the integral of the blue dotted curve = 2.2 and red dashed curve = 1.8.*

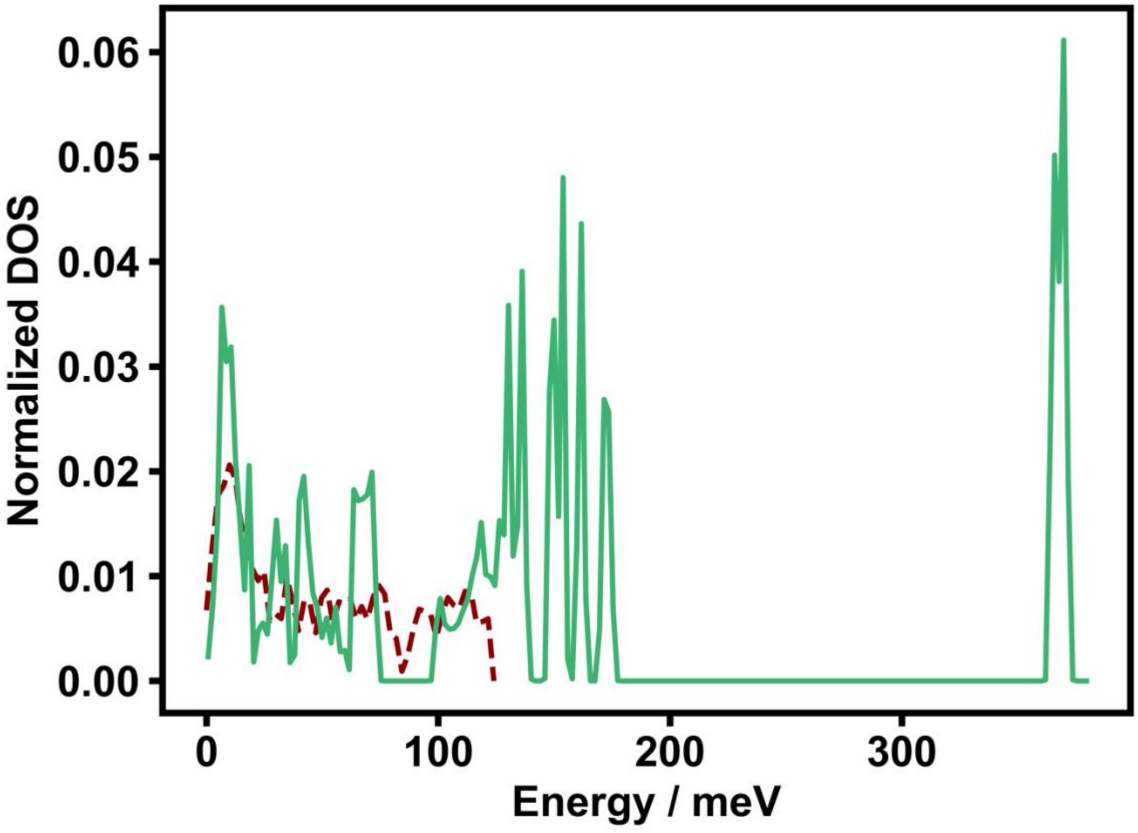

Figure S9. Phonon density of states of PVC with the ALIGNN output (red dashed) and the MACE-MP-0-3b (green solid) *normalized to the same scale such that the integral of the red dashed curve = 2.2.*

Supplemental Note 7:

The effect of different phonon density of states estimations on VDE heat capacity outputs is compared for the example materials, similar to the analysis shown in Figure 3 of the main text. The ALIGNN model, the MACE model (with the estimation procedures as mentioned in Supplemental Note 6), and the Debye model were each used as phonon density of states inputs into the VDE model, and the resulting VDE heat capacity estimations are shown in Figure S10.

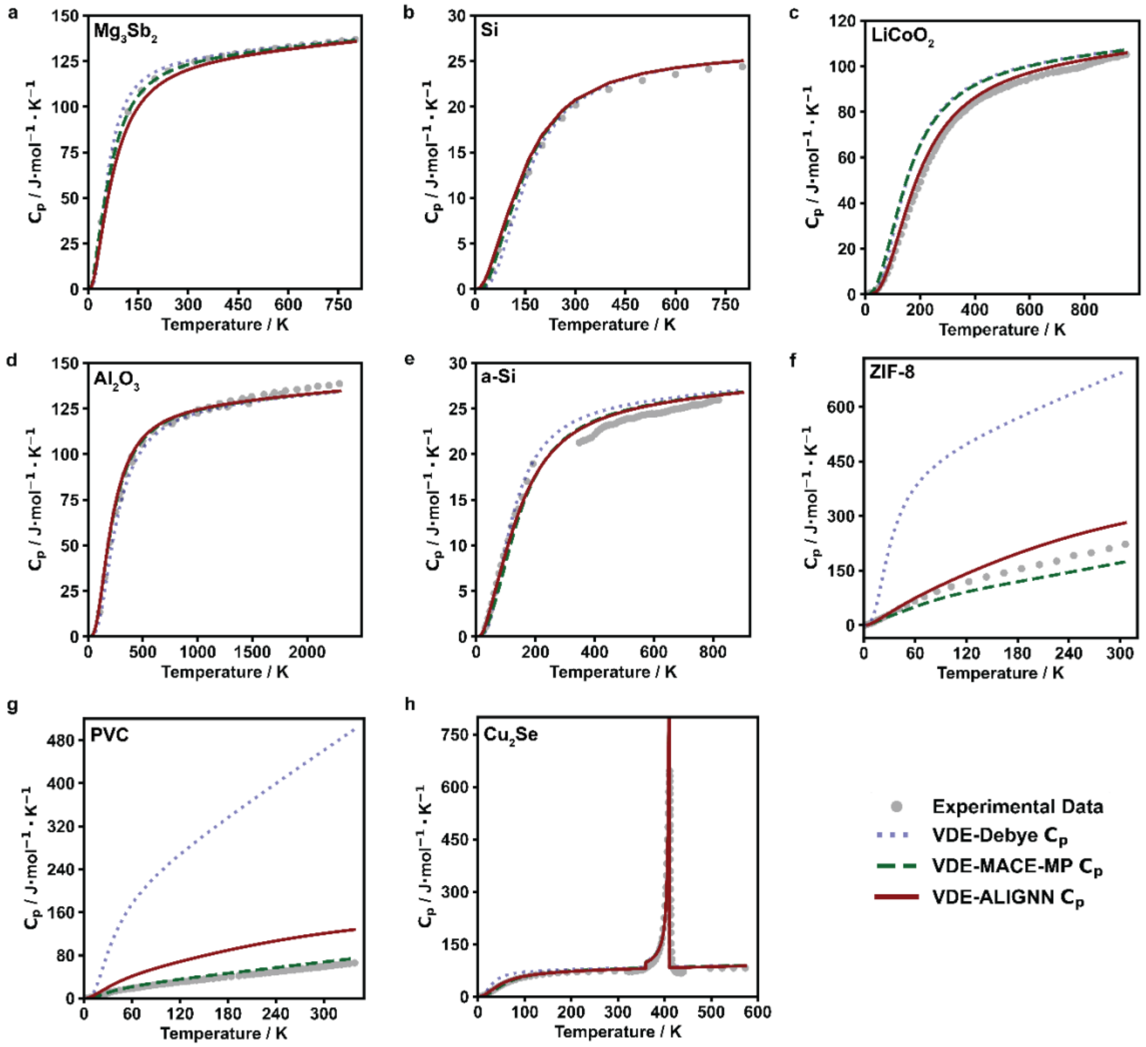

Figure S10. *Temperature dependent heat capacity estimates from the VDE model with density of states from the ALIGNN model (red line), the MACE-MP-0-3b model*[2] *with D3 dispersion (green dashed line), and Debye model (blue dotted line) for* **(a)** *Mg_3Sb_2,* **(b)** *Si,* **(c)** *$LiCoO_2$,* **(d)** *Al_2O_3,* **(e)** *a-Si,* **(f)** *ZIF-8,* **(g)** *PVC, and* **(h)** *Cu_2Se. Experimental values for Mg_3Sb_2,*[31] *Si,*[32] *$LiCoO_2$,*[33,34] *Al_2O_3,*[35–38] *a-Si,*[39,40] *ZIF-8,*[41] *PVC,*[42] *and Cu_2Se*[43–45] *are shown as gray points.*

For several of the materials (e.g. Mg3Sb2, Si, Al2O3, and Cu2Se), the three different phonon density of states estimates provide comparable heat capacity estimates, indicating that the dilation term used in the VDE model greatly contributes to the accuracy in the VDE model in those cases (when

comparing to the Debye estimates in Figures 4 and 5 of the main text, for example). However, in other cases such as that of LiCoO2, ZIF-8, a-Si, and PVC, one or the other of the machine learned models provides the most accurate heat capacity estimation when using the VDE model. This indicates that in some cases, a rough DOS estimation is acceptable when used in conjunction with a reliable dilation term; however, one cannot necessarily trust that the Debye model would be sufficient for every material that one would be interested in. In some cases, it might be useful to save computing time with the simplest estimate, but for not significantly more time and computing resources, the machine learned density of states estimates could more reliably predict heat capacity values for a wider range of materials.

Supplemental Note 8:

Thermal broadening is a technique used to smooth discontinuities resulting from piecewise functions of the phase fraction of a given material used in the phase differential for the heat capacity estimation, as in Eq. 10 in the main text. The discontinuous increase in the VDE estimation at the onset of the phase transformation is an artifact of the algorithms used to estimate ϕ_β carried through the calculations, resulting in the shift of a derivative over a linear to a non-linear region.[46] Thermal broadening algorithms have been used in previous works to alleviate this in heat capacity estimates.

Thermal broadening was applied to the VDE heat capacity estimation for Cu_2Se. The algorithm applied is a rolling average over a 3 K temperature range. The algorithm was applied three times, and the resulting trace (Figure S11) is a smoother trace than the original discontinuity.

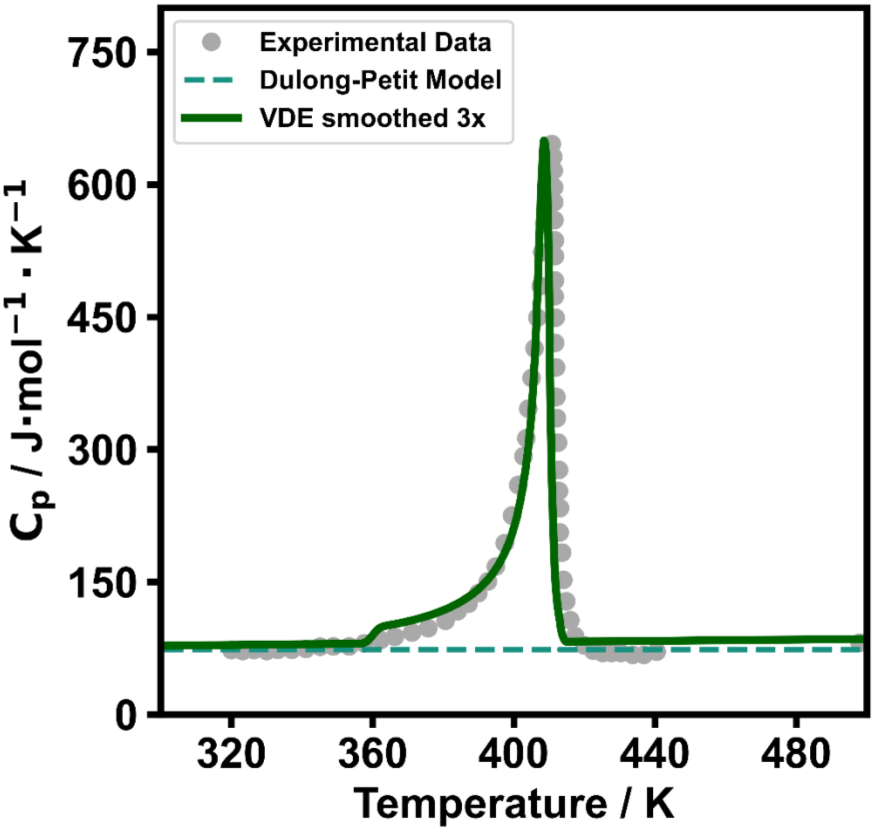

Figure S11. *Heat capacity estimations of Cu_2Se with the Dulong-Petit model (teal dotted line) and the VDE model with the added phase change contribution run through the thermal broadening algorithm three times (green). Models are compared to experimental values*[43–45] *(gray points).*

Supplemental Note 9:

The heat capacity across a multiphase system is described by the contribution of intrinsic heat capacity $C_{\mathrm{p}\phi}$ at temperature T and pressure p of the phases present in the relation

$$C_{\mathrm{p}} = \left(\frac{\partial H}{\partial T}\right)_{\mathrm{p}} = C_{\mathrm{p}\phi} + \Delta H \left(\frac{\partial \phi}{\partial T}\right)_{\mathrm{p}}$$

where ΔH represents the enthalpy associated with the phase transition. For a two-phase system with phases α and β, the total heat capacity can be described by each phase's contribution and the fraction ϕ of each phase in the relation[46]

$$C_{\mathrm{p}\phi} = \phi_{\alpha}(C_{\mathrm{V}}^{\alpha} + \Gamma_{\alpha}T) + \phi_{\beta}\left(C_{\mathrm{V}}^{\beta} + \Gamma_{\beta}T\right),$$

where $\phi_{\alpha} + \phi_{\beta} = 1$ and the dilation terms of each phase are represented by $\Gamma = \mathrm{B}\alpha_{\mathrm{V}}^{2}$. In many materials, each phase has approximately the same number of atoms/volume such that their high temperature heat capacities attain similar values. Thus, approximating C_{V} using a singular phase of the material is often sufficient.[46] As shown throughout this work, C_{V} is the largest component of C_{p}, and the dilation term only accounts for a small percentage. Due to similar expansion of phases, it is also sufficient to approximate the dilation terms as one phase as well. Therefore, in practice, $C_{\mathrm{p}\phi}$ has a negligible dependence on $\phi(T)$. As a result, we have approximated $C_{\mathrm{p}\phi}$ for Cu_2Se using only the material information for the low temperature α phase as

$$C_{\mathrm{p}\phi} \approx (C_{\mathrm{V}}^{\alpha} + \Gamma_{\alpha}T),$$

which is an approximation that holds even when the material is in the β phase, above the phase transition region from 410-660 K.

Supplemental Note 10:

As a test of how the ALIGNN phonon density of states output responds to slight volume changes in the same structure, a test of Si (mp-149) was run. Lattice parameter scaling of the structure was set to 1.0 (original scaling), 0.99, and 1.01, and the ALIGNN phonon density of states was calculated. The output shows a slight change in the phonon density of states from the original structure to the scaled structures. However, the outputs from 0.99 and 1.01 were identical, indicating that these changes in the lattice parameter scaling does impact the phonon density of states, but that the direction of scaling (e.g. slightly larger or slightly smaller) makes little difference at this scale.

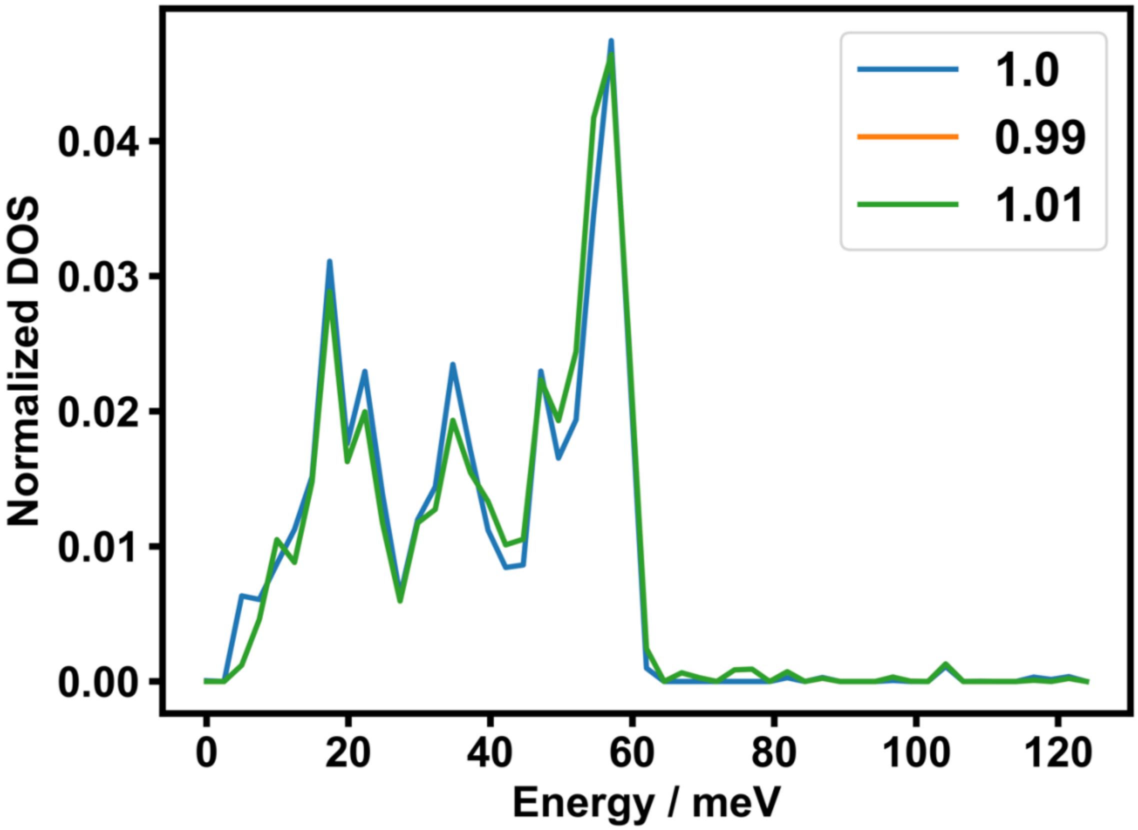

Figure S12. *Phonon density of states estimations from the ALIGNN model of Si at the original lattice parameter scaling (blue), 0.99 scaling (orange, hidden by the green trace which directly overlaps), and 1.01 (green).*

References

1. Jain, A., Ong, S.P., Hautier, G., Chen, W., Richards, W.D., Dacek, S., Cholia, S., Gunter, D., Skinner, D., Ceder, G., et al. (2013). Commentary: The Materials Project: A materials genome approach to accelerating materials innovation. APL Mater. *1*, 011002. https://doi.org/10.1063/1.4812323.

2. Batatia, I., Benner, P., Chiang, Y., Elena, A.M., Kovács, D.P., Riebesell, J., Advincula, X.R., Asta, M., Avaylon, M., Baldwin, W.J., et al. (2024). A foundation model for atomistic materials chemistry. Preprint at arXiv, https://doi.org/10.48550/arXiv.2401.00096 https://doi.org/10.48550/arXiv.2401.00096.

3. Grimme, S., Antony, J., Ehrlich, S., and Krieg, H. (2010). A consistent and accurate *ab initio* parametrization of density functional dispersion correction (DFT-D) for the 94 elements H-Pu. J. Chem. Phys. *132*, 154104. https://doi.org/10.1063/1.3382344.

4. Togo, A. (2023). First-principles Phonon Calculations with Phonopy and Phono3py. J. Phys. Soc. Jpn. *92*, 012001. https://doi.org/10.7566/JPSJ.92.012001.

5. Togo, A., Chaput, L., Tadano, T., and Tanaka, I. (2023). Implementation strategies in phonopy and phono3py. J. Phys. Condens. Matter *35*, 353001. https://doi.org/10.1088/1361-648X/acd831.

6. Ganose, A., Sahasrabuddhe, H., Asta, M., Beck, K., Biswas, T., Bonkowski, A., Bustamante, J., Chen, X., Chiang, Y., Chrzan, D., et al. (2025). Atomate2: Modular workflows for materials science. Preprint, https://doi.org/10.26434/chemrxiv-2025-tcr5h https://doi.org/10.26434/chemrxiv-2025-tcr5h.

7. Thomas C. Allison (2013). NIST-JANAF Thermochemical Tables - SRD 13. Version 1.0.2 ([object Object]). https://doi.org/10.18434/T42S31 https://doi.org/10.18434/T42S31.

8. Kresse, G., and Furthmüller, J. (1996). Efficient iterative schemes for ab initio total-energy calculations using a plane-wave basis set. Phys. Rev. B *54*, 11169–11186. https://doi.org/10.1103/PhysRevB.54.11169.

9. Kresse, G., and Furthmüller, J. (1996). Efficiency of ab-initio total energy calculations for metals and semiconductors using a plane-wave basis set. Comput. Mater. Sci. *6*, 15–50. https://doi.org/10.1016/0927-0256(96)00008-0.

10. Kresse, G., and Hafner, J. (1993). *Ab initio* molecular dynamics for liquid metals. Phys. Rev. B *47*, 558–561. https://doi.org/10.1103/PhysRevB.47.558.

11. Kresse, G., and Hafner, J. (1994). *Ab initio* molecular-dynamics simulation of the liquid-metal–amorphous-semiconductor transition in germanium. Phys. Rev. B *49*, 14251–14269. https://doi.org/10.1103/PhysRevB.49.14251.

12. Blöchl, P.E. (1994). Projector augmented-wave method. Phys. Rev. B *50*, 17953–17979. https://doi.org/10.1103/PhysRevB.50.17953.

13. Kresse, G., and Joubert, D. (1999). From ultrasoft pseudopotentials to the projector augmented-wave method. Phys. Rev. B *59*, 1758–1775. https://doi.org/10.1103/PhysRevB.59.1758.

14. Perdew, J.P., Burke, K., and Ernzerhof, M. (1996). Generalized Gradient Approximation Made Simple. Phys. Rev. Lett. *77*, 3865–3868. https://doi.org/10.1103/PhysRevLett.77.3865.

15. Togo, A., and Tanaka, I. (2015). First principles phonon calculations in materials science. Scr. Mater. *108*, 1–5. https://doi.org/10.1016/j.scriptamat.2015.07.021.

16. Gonze, X., and Lee, C. (1997). Dynamical matrices, Born effective charges, dielectric permittivity tensors, and interatomic force constants from density-functional perturbation theory. Phys. Rev. B *55*, 10355–10368. https://doi.org/10.1103/PhysRevB.55.10355.

17. Lewis, D.W., Ruiz-Salvador, A.R., Gómez, A., Rodriguez-Albelo, L.M., Coudert, F.-X., Slater, B., Cheetham, A.K., and Mellot-Draznieks, C. (2009). Zeolitic imidazole frameworks: structural and energetics trends compared with their zeolite analogues. CrystEngComm *11*, 2272–2276. https://doi.org/10.1039/B912997A.

18. Tan, J.C., Bennett, T.D., and Cheetham, A.K. (2010). Chemical structure, network topology, and porosity effects on the mechanical properties of Zeolitic Imidazolate Frameworks. Proc. Natl. Acad. Sci. *107*, 9938–9943. https://doi.org/10.1073/pnas.1003205107.

19. Chester, A.M., Castillo-Blas, C., Sajzew, R., Rodrigues, B.P., Lampronti, G.I., Sapnik, A.F., Robertson, G.P., Mazaj, M., Irving, D.J.M., Wondraczek, L., et al. (2024). Loading and thermal behaviour of ZIF-8 metal–organic framework-inorganic glass composites. Dalton Trans. *53*, 10655–10665. https://doi.org/10.1039/D4DT00894D.

20. Chapman, K.W., Halder, G.J., and Chupas, P.J. (2009). Pressure-Induced Amorphization and Porosity Modification in a Metal—Organic Framework. J. Am. Chem. Soc. *131*, 17546–17547. https://doi.org/10.1021/ja908415z.

21. Deringer, V.L., Bernstein, N., Bartók, A.P., Cliffe, M.J., Kerber, R.N., Marbella, L.E., Grey, C.P., Elliott, S.R., and Csányi, G. (2018). Realistic Atomistic Structure of Amorphous Silicon from Machine-Learning-Driven Molecular Dynamics. J. Phys. Chem. Lett. *9*, 2879–2885. https://doi.org/10.1021/acs.jpclett.8b00902.

22. Ivashchenko, V.I., Turchi, P.E.A., and Shevchenko, V.I. (2007). Simulations of the mechanical properties of crystalline, nanocrystalline, and amorphous SiC and Si. Phys. Rev. B *75*, 085209. https://doi.org/10.1103/PhysRevB.75.085209.

23. Kluge, M.D., and Ray, J.R. (1988). Elastic constants and density of states of a molecular-dynamics model of amorphous silicon. Phys. Rev. B *37*, 4132–4136. https://doi.org/10.1103/PhysRevB.37.4132.

24. Albaret, T., Tanguy, A., Boioli, F., and Rodney, D. (2016). Mapping between atomistic simulations and Eshelby inclusions in the shear deformation of an amorphous silicon model. Phys. Rev. E *93*, 053002. https://doi.org/10.1103/PhysRevE.93.053002.

25. Huan, T.D., and Ramprasad, R. (2020). Polymer Structure Prediction from First Principles. J. Phys. Chem. Lett. *11*, 5823–5829. https://doi.org/10.1021/acs.jpclett.0c01553.

26. PVC Properties Vinidex Pty Ltd. https://www.vinidex.com.au/technical-resources/material-properties/pvc-properties/.

27. Jayanna, H.S., and Subramanyam, S.V. (1992). Thermal expansion of irradiated polyvinyl chloride from 10 K to 340 K. Polym. Bull. *28*, 481–487. https://doi.org/10.1007/BF00297343.

28. Shackelford, J.F., and Doremus, R.H. eds. (2008). Ceramic and Glass Materials: Structure, Properties and Processing (Springer Science+Business Media, LLC) https://doi.org/10.1007/978-0-387-73362-3.

29. Hegde, V.I., Tan, J.-C., Waghmare, U.V., and Cheetham, A.K. (2013). Stacking Faults and Mechanical Behavior beyond the Elastic Limit of an Imidazole-Based Metal Organic Framework: ZIF-8. J. Phys. Chem. Lett. *4*, 3377–3381. https://doi.org/10.1021/jz4016734.

30. Gurunathan, R., Choudhary, K., and Tavazza, F. (2023). Rapid prediction of phonon structure and properties using the atomistic line graph neural network (ALIGNN). Phys. Rev. Mater. *7*, 023803. https://doi.org/10.1103/PhysRevMaterials.7.023803.

31. Agne, M.T., Imasato, K., Anand, S., Lee, K., Bux, S.K., Zevalkink, A., Rettie, A.J.E., Chung, D.Y., Kanatzidis, M.G., and Snyder, G.J. (2018). Heat capacity of Mg3Sb2, Mg3Bi2, and their alloys at high temperature. Mater. Today Phys. *6*, 83–88. https://doi.org/10.1016/j.mtphys.2018.10.001.

32. Porter, L.J., Yip, S., Yamaguchi, M., Kaburaki, H., and Tang, M. (1997). Empirical bond-order potential description of thermodynamic properties of crystalline silicon. J. Appl. Phys. *81*, 96–106. https://doi.org/10.1063/1.364102.

33. Gotcu-Freis, P., Cupid, D.M., Rohde, M., and Seifert, H.J. (2015). New experimental heat capacity and enthalpy of formation of lithium cobalt oxide. J. Chem. Thermodyn. *84*, 118–127. https://doi.org/10.1016/j.jct.2014.12.007.

34. Kawaji, H., Takematsu, M., Tojo, T., Atake, T., Hirano, A., and Kanno, R. (2002). LOW TEMPERATURE HEAT CAPACITY AND THERMODYNAMIC FUNCTIONS OF LiCoO2.

35. Huang, L.-F., Lu, X.-Z., Tennessen, E., and Rondinelli, J.M. (2016). An efficient ab-initio quasiharmonic approach for the thermodynamics of solids. Comput. Mater. Sci. *120*, 84–93. https://doi.org/10.1016/j.commatsci.2016.04.012.

36. Munro, R.G. (1997). Evaluated Material Properties for a Sintered alpha-Alumina. J. Am. Ceram. Soc. *80*, 1919–1928. https://doi.org/10.1111/j.1151-2916.1997.tb03074.x.

37. Schauer, A. (1965). THERMAL EXPANSION, GRUENEISEN PARAMETER, AND TEMPERATURE DEPENDENCE OF LATTICE VIBRATION FREQUENCIES OF ALUMINUM OXIDE. Can. J. Phys. *43*, 523–531. https://doi.org/10.1139/p65-049.

38. Chase, M. (1998). NIST-JANAF Thermochemical Tables, 4th Edition (American Institute of Physics, -1).

39. Tsang, K.H., Kui, H.W., and Chik, K.P. (1993). Calorimetric studies of the heat capacity and relaxation of amorphous Si prepared by electron beam evaporation. J. Appl. Phys. *74*, 4932–4935. https://doi.org/10.1063/1.354329.

40. Queen, D.R., Liu, X., Karel, J., Metcalf, T.H., and Hellman, F. (2013). Excess Specific Heat in Evaporated Amorphous Silicon. Phys. Rev. Lett. *110*, 135901. https://doi.org/10.1103/PhysRevLett.110.135901.

41. Rosen, P.F., Calvin, J.J., Dickson, M.S., Katsenis, A.D., Friščić, T., Navrotsky, A., Ross, N.L., Kolesnikov, A.I., and Woodfield, B.F. (2019). Heat capacity and thermodynamic functions of crystalline forms of the metal-organic framework zinc 2-methylimidazolate, Zn(MeIm)2. J. Chem. Thermodyn. *136*, 160–169. https://doi.org/10.1016/j.jct.2019.05.008.

42. Chang, S.-S. (1977). Heat capacity and thermodynamic properties of poly(vinyl chloride). J. Res. Natl. Bur. Stand. *82*, 9. https://doi.org/10.6028/jres.082.002.

43. Liu, H., Shi, X., Xu, F., Zhang, L., Zhang, W., Chen, L., Li, Q., Uher, C., Day, T., and Snyder, G.J. (2012). Copper ion liquid-like thermoelectrics. Nat. Mater. *11*, 422–425. https://doi.org/10.1038/nmat3273.

44. Liu, H., Yang, J., Shi, X., Danilkin, S.A., Yu, D., Wang, C., Zhang, W., and Chen, L. (2016). Reduction of thermal conductivity by low energy multi-Einstein optic modes. J. Materiomics *2*, 187–195. https://doi.org/10.1016/j.jmat.2016.05.006.

45. Brown, D.R., Heijl, R., Borup, K.A., Iversen, B.B., Palmqvist, A., and Snyder, G.J. (2016). Relating phase transition heat capacity to thermal conductivity and effusivity in Cu_2Se. Phys. Status Solidi RRL – Rapid Res. Lett. *10*, 618–621. https://doi.org/10.1002/pssr.201600160.

46. Agne, M.T., Voorhees, P.W., and Snyder, G.J. (2019). Phase Transformation Contributions to Heat Capacity and Impact on Thermal Diffusivity, Thermal Conductivity, and Thermoelectric Performance. Adv. Mater. *31*, 1902980. https://doi.org/10.1002/adma.201902980.
